# Study of tribenzo[*b*,*d*,*f*]azepine as donor in D–A photocatalysts

**DOI:** 10.3762/bjoc.21.76

**Published:** 2025-05-14

**Authors:** Katy Medrano-Uribe, Jorge Humbrías-Martín, Luca Dell’Amico

**Affiliations:** 1 Department of Chemical Sciences, University of Padova, Via Francesco Marzolo 1, 35131, Padova, Italy

**Keywords:** donor–acceptor system, photocatalyst design, photoredox catalysis, organic photocatalyst

## Abstract

Since the discovery of donor–acceptor (D–A) type molecules in the field of materials science, they have found great applicability in the field of photocatalysis. Most of these compounds are based on complex D–A–D structures or multi-D–A systems, such as 4CzIPN. Whereas these systems have been widely studied and applied as photocatalysts, simpler D–A structures remain less explored. Nevertheless, the simplicity of D–A structures makes them the ideal structures to further understand the structure–property relationship of D–A molecules for optimizing their photocatalytic performance by simpler modification of the different D–A subunits. In particular, D–A structures featuring sulfur-based acceptors and nitrogen donors have gained increasing attention for their use as photoredox catalysts. This study introduces a new family of D–A molecules by exploring various sulfur-based acceptors and nitrogen donors, including a novel tribenzo[*b,d,f*]azepine (TBA) unit and 5*H*-dibenz[*b,f*]azepine (IMD). Our findings demonstrate that these simple D–A structures exhibit promising photocatalytic properties, comparable to those of more complex D–A–D systems.

## Introduction

**Figure 1:**
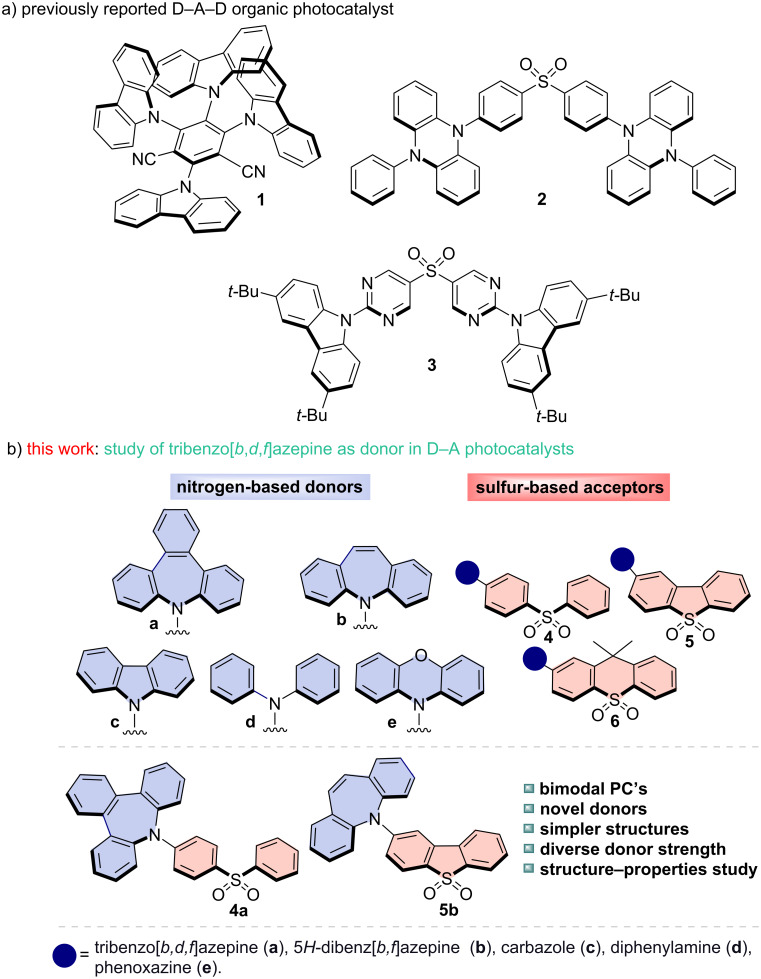
D–A–D organic PCs previously reported and our new D–A bimodal organic PCs.

In recent years, photocatalysis has emerged as a powerful tool for the construction and functionalization of organic molecules and materials. Thus, the scientific community has focused on the design and study of new organic molecules that can be used as photocatalysts, replacing generally more expensive metal-based complexes [1–3]. Furthermore, there is a particular interest in the obtainment of organic molecules with well-balanced redox potentials in the excited state that can act as bimodal photocatalysts, facilitating their use in oxidative and reductive quenching cycles. In this sense, it is crucial to understand the molecule's structure–properties dependence to modulate its optical and photoredox properties [4]. For instance, molecules with donor–acceptor (D–A) structures, classically used as OLED emitters, have gained relevance by finding alternative applications in the field of photocatalysis [5]. In this type of structure, the electron density distribution in the charge transfer (CT) excited state is facilitated by the presence of an electron-rich moiety and an electron-poor part in the same molecule, increasing the lifetime in the excited state. One of the representative classes of molecules demonstrating dual use in materials chemistry and photocatalysis is the carbazolyl dicyanobenzene (CDCB) family. Since the initial report on the synthesis and photoluminescence study of 4CzIPN (**1**, Figure 1a) [6], the scientific community has recognized its potential under photocatalytic manifolds. This interest is attributed to: i) its absorption profile in the visible region, ii) a long lifetime of the excited states, and iii) balanced redox potentials in both the ground and excited states [7]. In 2018, Zeitler and her collaborators conducted an innovative and in-depth study on modulating the photochemical properties of a family of donor–acceptor cyanoarenes [8]. They employed various nitrogen donor molecules attached to diversely substituted acceptor cores. This systematic approach allowed the authors to develop new organic photocatalysts (PCs) with strong reductive or oxidative properties based on the different redox potentials.

Although diverse scaffolds have been reported in the literature, the identification and use of novel PCs with tunable and diverse optical and redox properties can pave the way to uncharted reactivity. In this context, sulfur-based cores, widely used as acceptors in photoelectric materials [9–14], and dyes [15–16] serve as promising structures for constructing and designing novel PCs. These structures show a high electron affinity, stability, and the possibility of tuning their physicochemical properties by substituting the two aromatic rings. In 2018, Sang Kwon and co-workers reported a computational study to design new PCs to be employed in atom transfer radical polymerization (O-ATRP) [17]. Notably, the sulfur-based structure **2** showed excellent performance for this transformation. One year later, the same research group reported its use in a reversible addition-fragmentation chain-transfer (RAFT) polymerization [18]. Moreover, in 2022, Zysman-Colman and collaborators showed that molecule **3**, initially synthesized as a TADF (thermally activated delayed fluorescence) emitter [14], can be used as a PC under electron-transfer (ET) and energy-transfer (EnT) processes (Figure 1a) [19]. All the main reports in the field focused on D–A–D (donor–acceptor–donor) structures. Quite surprisingly, the potential use as PCs of structurally simpler D–A molecules has been largely overlooked.

Aliphatic and aromatic nitrogen donors are widely used in synthesizing fluorescent emitters due to their electron-donating strength. The development of stronger donors to enhance luminescence remains a key area of research [20–22]. Recently, azepine-based analogs, such as tribenzo[*b,d,f*]azepine (TBA, **a**), have been explored due to their photoluminescence properties [23–27]. This antiaromatic core offers unique features, including twisted structures, reduced π–π stacking, and enhanced reverse intersystem crossing rates, becoming a better donor compared to fully planar compounds as carbazole (**c**). Similarly, 5*H*-dibenz[*b,f*]azepine (IMD, **b**) has been incorporated into D–A–D structures, showing interesting photophysical properties compared to common substrates like **c**, diphenylamine (**d**), and phenoxazine (**e**) [28–30]. However, their potential as D-unit in organic PCs remains unexplored. For this reason, studying this avenue could unlock new opportunities for the synthesis and design of more powerful, efficient and versatile organic photocatalysts.

We herein present the design, synthesis and study of a new sulfur-based D–A family using diverse nitrogen donors (Figure 1b). We performed complete photophysical characterization of the diverse D–A molecules to analyze the structure–properties relationships. We further studied their photocatalytic potential as bimodal PCs and demonstrated their potential use in different reductive and oxidative quenching processes.

## Results and Discussion

### Photophysical properties analysis

**Figure 2:**
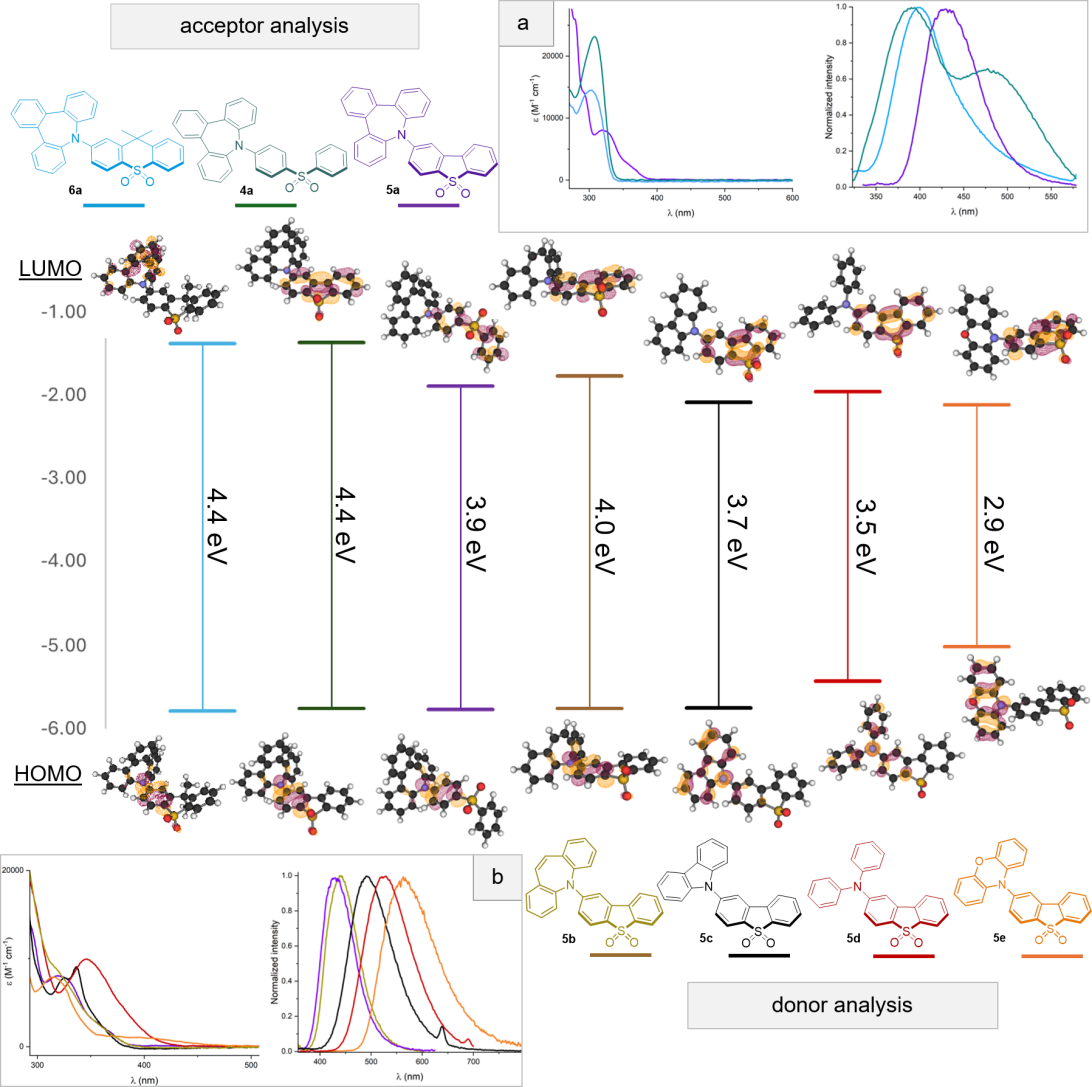
Selected frontier MOs and relative calculated energies of D–A photocatalysts (**4a**,**5a–e**, and **6a**). Absorption and emission profiles of D–A compounds (**4a**,**5a–e**, and **6a**) measured in MeCN.

We started our study with three different sulfur-based acceptors, namely: diphenyl sulfone (**4**), dibenzo[*b*,*d*]thiophene 5,5-dioxide (**5**), and 9,9-dimethyl-9*H*-thioxanthene 10,10-dioxide (**6**). The selection of these scaffolds was aimed at investigating the effect of conjugation and rigidity/flexibility on the presence of the same donor (TBA, **a**). In the case of the D–A compounds **4a** and **6a**, we observed a blue-shifted absorption profile due to the break of the conjugation in sulfur-based acceptors. Compounds **4a** and **6a** presented a similar absorption profile, while molecule **5a** showed a red-shifted spectrum tailing up to the visible region (Figure 2a). The lack of a significant charge transfer (CT) character in scaffolds **4a** and **6a** can be attributed to the absence of a complete conjugated system.

On the other hand, the fluorescence profile showed more differences in the analysis of the three members of the D–A family. Again, **5a** revealed a bathochromic effect compared with the less conjugated scaffolds. Interestingly, molecule **4a**, which has the most flexible acceptor core, exhibited a dual emission (DE) profile (Figure 2a). This behavior may be connected to the phenomenon known as PISP (photoinduced structural planarization), which has been reported for the TBA N-substituted with an electron-withdrawing group [31]. Additionally, it is possible that the mobility of core **4** contributes to this behavior, as evidenced by the observation that the DE is not present in the more rigid structures **5a** and **6a**. In this compound, we did not observe changes in the absorption profile during the solvatochromism analysis (see Supporting Information File 1, Figure S4). The structural characteristics of compound **5a** conferred the biggest value in terms of Stokes shift parameter, indicating an increased excited state's charge transfer (CT) character (Table 1). Similarly, this behavior was observed experimentally in the solvatochromism study of fluorescence using solvents with diverse polarities (see Supporting Information File 1, Figure S9). Indeed, the density functional theory (DFT) calculation performed at WB97XD/Def2TZVP level of theory showed the lowest value for the HOMO–LUMO energy gap in compound **5a** (3.9 eV) as a consequence of the extended π conjugation compared with **4a** and **6a** (4.4 and 4.4 eV, respectively). Interestingly, compound **6a**, which possesses the weakest sulfur-based acceptor, showed an inversion in the LUMO distribution, localizing it in the TBA core – this behavior of the named antiaromatic compound as an acceptor was previously reported (Figure 2) [31].

The dibenzo[*b,d*]thiophene 5,5-dioxide (**5**) was chosen for further investigation because of its red-shifted absorption. From a photochemical perspective, this characteristic can facilitate the use of less energetic light sources. Additionally, we aim to evaluate the unique effect of the TBA donor unit (**a**) compared to other donors. We next synthesized diverse D–A structures employing common nitrogen-based compounds widely used in materials chemistry like carbazole (**c**), diphenylamine (**d**), and phenoxazine (**e**). Furthermore, we wanted to study the diverse or similar properties between the antiaromatic molecules **a** and **b**, in which the main difference is the presence of a third aromatic ring. According to the literature, the presence of the third benzene ring in the TBA (**a**) differentiates the conformations of structures **a** and **b** in the excited state. This results in a consistently planar conformation for donor **b**, while donor **a** can exhibit either a planar or bent conformation, depending on the nature of the substituent, as previously mentioned. This duality between planar and bent shapes is significant, as it contributes to the aromatic character that is acquired in the excited state by structures that are antiaromatic in the ground state, following Baird’s rule. Intrigued by this diverse behavior, we wanted to investigate if the possible structural differences between both compounds (**5a** and **5b**) were important for photocatalytic activity.

Analyzing the diverse absorption profiles, we can observe an increase in the red-shifted behavior related to the donor strength in compounds **5e**, **5d**, and **5c**. In contrast, the azepine-derived compounds are the most blue-shifted (Table 1, entry 5). The same trend is observed in the emission (Figure 2b). The Stokes shift values for the classical nitrogen donors (**c**, **d,** and **e**) demonstrate a more pronounced CT character with respect to **5a** and **5b** (Table 1, entry 8), also corroborated by the theoretical descriptor Δr (Table 1, entry 11) that describes the charge transfer character [32–33]. Moreover, this CT behavior is supported by the DFT studies, which suggested a better spatial separation between the HOMO and LUMO. As expected, the HOMO–LUMO energy gap followed a trend that is dependent on the electron-donating capacity of the nitrogen heterocycles and amine present in compounds **5e** (2.9 eV), **5d** (3.5 eV)**,** and **5c** (3.7 eV). At the same time, **5a** and **5b** showed bigger values (3.9 eV and 4.0 eV, respectively) (Figure 2).

**Table 1:** Summary of the excited- and ground-state photoredox properties.^a^

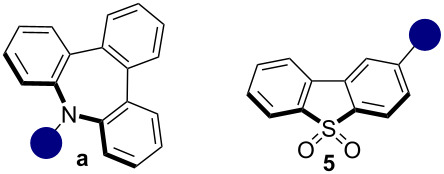								

Entry	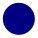 , PC	**4a**	**5a**	**6a**	**5b**	**5c**	**5d**	**5e**

1	*E*_ox_ (V)^a^	1.46	1.41	1.43	1.32	1.42	1.12	0.75
2	E*_ox_ (V)	−2.24	−1.89	−2.27	−1.88	−1.68	−1.78	−1.85
3	*E*_red_ (V)^a^	−2.35	−1.95	−2.4	−1.96	−1.75	−1.86	−1.74
4	E*_red_ (V)	1.35	1.35	1.3	1.24	1.35	1.04	0.86
5	λ_abs_ (nm)	308	320	292	312	336	346	393
6	λ_em_(nm)	400,478	430	398	441	493	525	564
7	*E*_0,0_ (eV)	3.7	3.3	3.7	3.2	3.1	2.9	2.6
8	Stokes shift (nm)	81	110	106	129	157	179	171
9	τ (ns)	0.9^b^	2.2^b^	2.0^b^	0.7	11.6	9.1	4.4^b^
10	QY (%)	12	7	10	6	14	14	16
11	Δr^c^	3.31 Å	2.62 Å	2.82 Å	2.40 Å	3.57 Å	3.52 Å	4.97 Å

^a^All potentials were measured in MeCN. Values are reported in V versus SCE (see Supporting Information File 1). ^b^τ_AvInt_. ^c^The Δr parameter describes the charge transfer character.

The strength of common donors plays a crucial role in influencing quantum yield (QY) measurements. As shown in Table 1, we observe a notable decrease in QY across the PCs **5e**, **5d**, and **5c**, with values of 16%, 14%, and 14%, respectively. The lowest values were obtained for molecules **5a** and **5b** (7% and 6%, each).

Remarkably, compound **5e** demonstrated minimal luminescence in nearly all solvents at room temperature. This behavior has been previously reported and is believed to be due to strong CT stabilization of the first excited state of the molecule [34]. This observation is further supported by the orthogonal D–A conformation calculated using DFT, which indicates a decoupled interaction between the HOMO and the LUMO (Figure 2). Moreover, compound **5e** is the only member of the family in which the HOMO orbital is not delocalized in one of the aromatic rings of the acceptor core.

### Redox properties analysis

We started our analysis by looking at the impact of the diverse sulfur-based cores on the redox properties. Here, we can observe similar *E*_ox_ values ranging from 1.41 V to 1.46 V vs SCE. This behavior is consistent with preserving the same donor core (**a**) within the structure. In contrast, a significant difference was observed for the *E*_red_ values. By adjusting the acceptor strength of the sulfur core, we observed a trend where the D–A structure with the weakest acceptor (**6a**) yielded the most negative value (*E*_red_ = −2.4 vs SCE) (see Supporting Information File 1, Table S1). In contrast, molecule **5a**, which has the strongest acceptor displayed the most positive one (*E*_red_ = −1.9 V vs SCE).

**Figure 3:**
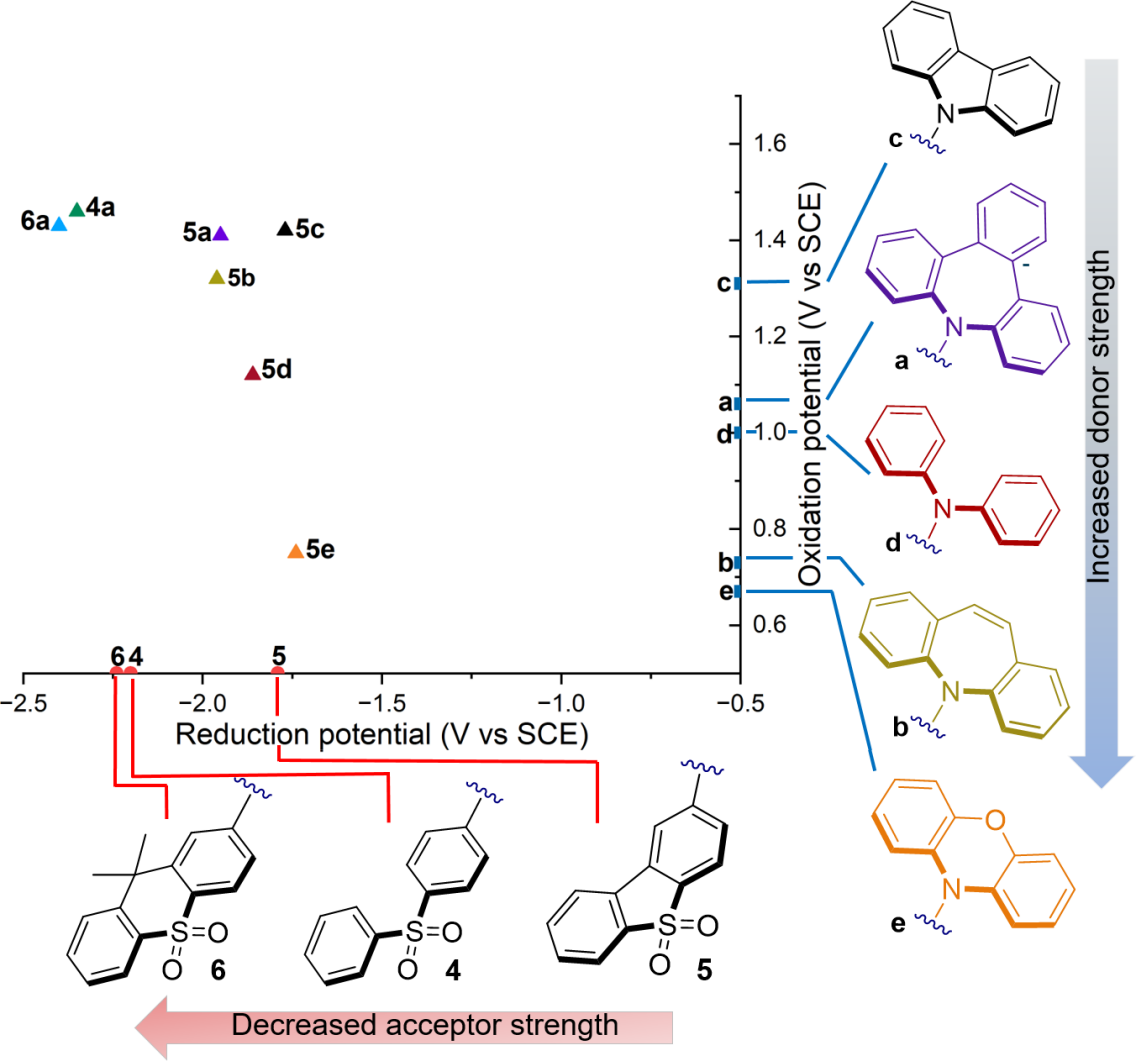
Comparison of the ground state redox potential of the acceptor moieties (**4**–**6**), the donor moieties (**a**–**e**), and D–A compounds (**4a**, **5a–e**, and **6a**).

We next investigated the diverse donors. For D–A molecules **5c**, **5d**, and **5e**, the redox potential calculated for the ground state is slightly more positive than the one measured for the single donor (**c**, **d**, and **e**, respectively). For example, for phenoxazine (**e**) we measured an *E*_ox_ = 0.67 V, while for compound **5e** the *E*_ox_ = 0.75 V vs SCE. In contrast, the azepine cores (**a** and **b**) showed a stronger impact in the *E*_ox_ of the D–A structures. For instance, IMD (**b**) with an oxidation potential of 0.73 V when present in the molecule **5b** resulted in a considerably different *E*_ox_ of 1.32 V (Figure 3).

A molecule that in the excited state exhibits both strong oxidative power (E*_ox_ up to −1.5 V) and strong reductive power (E*_red_ up to 1.5 V) can be classified as a bimodal photocatalyst. This type of molecule is capable of driving both oxidative and reductive reactions, thereby offering significant versatility to achieve photocatalytic transformations. To our delight, molecule **5a** possesses a promising E*_ox_ =−1.89 V vs SCE (Table 1, entries 1 and 2) and a useful *E*_ox_ = 1.41 V. For *E*_red_, **5a** maintains a good balance between the redox potentials in both the ground and excited states, showing values of *E*_red_ = −1.95 V and E*_red_ = 1.35 V vs SCE (Table 1, entries 3 and 4). Comparing it with its analog **5b**, we observe similar redox potentials except for *E*_ox_ and E*_red_ values.

The redox window is more limited for the other members of the D–A family. For example, for molecule **5e** the *E*_ox_ is 0.75 V, which is the lowest value among all family members (Table 1, entry 1). This observation can be explained by the nonexistent electronic coupling between the donor and the acceptor due to the highly twisted structure [35] as shown in the HOMO. As a consequence, the *E*_ox_ of molecule **5e** is similar to the *E*_ox_ of the phenoxazine core, with respect to the rest of the family (**5a**–**d**) that possesses higher *E*_ox_ since their HOMO is localized in both the donor and acceptor.

### Photocatalytic studies and synthetic applications

After establishing structure–property relationships, we aimed to use the synthesized donor–acceptor (D–A) compounds to investigate their photocatalytic activity. We found that most members of the D–A family exhibited promising redox potentials in their excited states, indicating their potential to function as effective bimodal photocatalysts. Additionally, our photophysical characterization provided essential insights into their behavior in the excited state and stability. We initiated the study of the photocatalytic activity of all family members in an oxidative quenching cycle for the dehalogenation of 4-bromobenzonitrile (**7**). Typically, this type of chemical transformation requires highly reducing PCs or the use of UV light [36]. First, we evaluated the photocatalytic performance of molecules **4a**, **5a**, and **6a** (see Supporting Information File 1, Table S3). As we expected, due to the blue-shifted absorption presented in molecules **4a** and **6a**, it was impossible to excite them under visible light (400 nm). Gratifyingly, PC **5a** delivered product **8** with a promising 63% NMR yield.

Next, we compare the photocatalytic behavior of compound **5a** with the other family members utilizing the same dehalogenation manifold. Here, even slightly changes in the redox properties have an influence on the yield of the reaction. The D–A with the azepine analog (**5b**), gave the dehalogenated product **8** in 58% NMR yield (Table 2, entry 2). Quite surprisingly, **5e** showed only traces of **8**, even with an E*_ox_ of −1.85 V (Table 2, entry 5).

**Table 2:** Dehalogenation of 4-bromobenzonitrile (**7**).

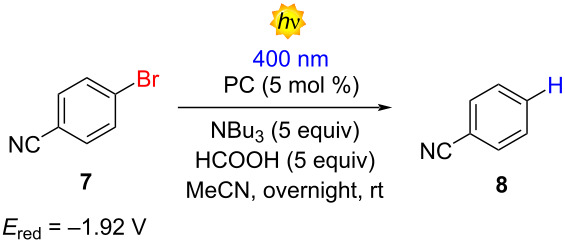		

Entry	PC	^1^H NMR yield^a^ (%)

**1**	**5a**	63
**2**	**5b**	58
**3**	**5c**	56
**4**	**5d**	44
**5**	**5e**	traces

^a^CH_2_Br_2_ as internal standard.

Under the oxidative quenching study, we also evaluated the photocatalytic potential of the new family of D–A compounds in the atom transfer radical addition (ATRA) reaction involving styrene and tosyl chloride (TsCl), as previously reported by Zysman-Colman and co-workers [19]. Compound **5d** showed the best performance with a 27% calculated NMR yield (20%, isolated yield) (Table 3, entry 3), while the azepine derivatives **5a** and **5b** led the transformation at 13 and 8%, respectively (Table 3, entries 1 and 2). However, these results are comparable to those obtained by the same author using the well-established PCs **1** and **3** (Table 3, entries 6 and 7)

**Table 3:** ATRA reaction between tosyl chloride (**9**) and styrene (**10**).

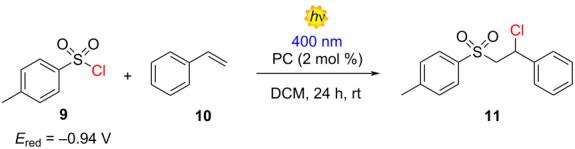		

Entry	PC	Yield^a^ (%)

**1**	**5a**	13
**2**	**5b**	8
**3**	**5c**	27 (20)
**4**	**5d**	12
**5**	**5e**	21
**6** ^b^	**1**	10
**7** ^b^	**3**	16

^a^Yields determined by ^1^H NMR analysis of the crude mixture using CH_2_Br_2_ as internal standard. Isolated yield in parentheses. ^b^Yields reported in reference [19].

Next, we wanted to analyze the use of the PCs in reductive quenching mechanisms. For this purpose, we selected the Giese-type addition between the *N*-Cbz-Pro (**12**, *E*_ox_ = 0.95 V vs SCE) and the dimethyl maleate (**13**), which is a standard benchmark reaction for the evaluation of novel PCs [37]. In this case, we obtained the best result using compound **5a** with a 76% NMR yield (65%, isolated yield) (Table 4, entry 1). Compounds **5b** and **5c**, whose redox potential in the ground and excited state are similar to **5a**, lead to the formation of the **14** in 59% and 65% NMR yield, each (Table 4, entries 2 and 3). Interestingly, compounds **5d** and **5e** showed the worst photocatalytic performances that can be attributed to their inferior E*_red_ (Table 4, entries 4 and 5). Gratifyingly, our PC **5a** showed a better performance in comparison with the D–A–D compound **3** (Table 3, entry 7).

**Table 4:** Giese addition using N-Cbz-Pro (**12**) and dimethyl maleate (**13**).

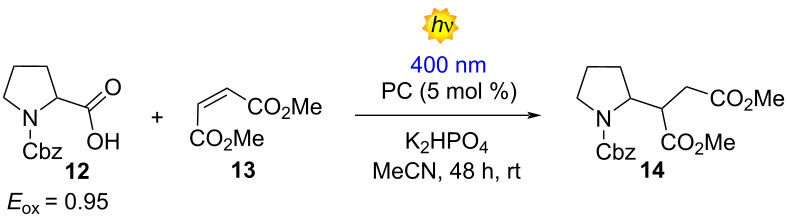		

Entry	PC	Yield^a^ (%)

**1**	**5a**	78 (65)
**2**	**5b**	59
**3**	**5c**	65
**4**	**5d**	43
**5**	**5e**	5
**6** ^b^	**1**	99
**7** ^b^	**3**	64

^a^Yields determined by ^1^H NMR analysis of the crude mixture using CH_2_Br_2_ as internal standard. Isolated yield in parentheses. ^b^Yields reported in reference [19].

Furthermore, we obtained pleasing outcomes when we tried the photocatalyzed reductive pinacol coupling of benzaldehyde (**15**), as reported by Rueping [38]. In this methodology, the reduction of compound **15** is facilitated by reduced photocatalyst (PC) and the interaction of **15** with the radical cation of DIPEA. The best result, again, was attributed to molecule **5a** with 60% isolated yield (Table 5, entry 1). In contrast, molecule **5b** showed the worst performance with 41% NMR yield (Table 5, entry 2). For compounds **5c**–**e**, the NMR yield calculated for product **16** was similar (55–51%), probably due to the comparable reductive properties in both ground and excited states (Table 5, entries 3, 4 and 5). Unfortunately, for this reaction, all the members of the D–A family delivered the product in a lower yield compared with molecules **1** and **3** (Table 5, entries 6 and 7).

**Table 5:** Pinacol coupling of benzaldehyde (**15**).

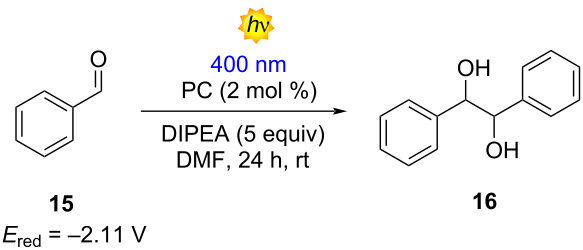		

Entry	PC	Yield^a^ (%)

**1**	**5a**	69 (60)
**2**	**5b**	41
**3**	**5c**	55
**4**	**5d**	51
**5**	**5e**	51
**6** ^b^	**1**	76
**7** ^b^	**3**	80

^a^Yields determined by ^1^H NMR analysis of the crude mixture using CH_2_Br_2_ as internal standard and refer to the combined yield of meso:dl isomers. Isolated yield in parenthesis. ^b^Yields reported in reference [19].

## Conclusion

In conclusion, we explored the potential of tribenzo[*b,d,f*]azepine (TBA) as a donor in donor–acceptor (D–A) organic photocatalysts (PCs). We synthesized a new series of sulfur-based D–A compounds and compared their photophysical and photoredox properties with TBA, its analog 5*H*-dibenz[*b,f*]azepine (IMD), and common nitrogen donors. The excited state redox potentials of these compounds suggest their suitability for challenging photocatalytic reactions through oxidative and reducing quenching cycles. TBA showed a well-balanced redox window, making it a promising candidate for new PC designs. While TBA and IMD displayed similar characteristics, the D–A IMD compound showed a shorter lifetime, which proved unfavorable in photocatalytic tests. The differing excited state conformations (bend vs planar) reported for these azepine analogs did not negatively impact photocatalytic activity, showing similar results in some of the benchmark reactions carried out during this analysis. Our findings suggest that antiaromatic compounds like TBA could replace traditional nitrogen donors in PCs, offering good redox potentials and competitive photophysical properties in addition to the previously reported characteristics like highly twisted structures that can be useful in designing new PCs with TADF behavior. We hope this study inspires the construction of new PCs that could combine azepine derivatives, exemplifying the valuable incorporation of widely used structures in materials chemistry to photocatalysis.

## Supporting Information

File 1 Reactivity studies, general experimental procedures, product isolation and characterization, spectroscopic data for new compounds, and copies of NMR spectra.

## Data Availability

All data that supports the findings of this study is available in the published article and/or the supporting information of this article.
