# Supplementary material for: Study of tribenzo[b,d,f]azepine as donor in D–A photocatalysts
Source: Beilstein J Org Chem. 2025 May 14;21:935–44. doi: 10.3762/bjoc.21.76 (PMC12117207; doi:10.3762/bjoc.21.76)
Supplement: File 1 — Reactivity studies, general experimental procedures, product isolation and characterization, spectroscopic data for new compounds, and copies of NMR spectra. [file Beilstein_J_Org_Chem-21-935-s001.pdf]

BEILSTEIN JOURNAL OF ORGANIC CHEMISTRY

Supporting Information

for

Study of tribenzo[*b*,*d*,*f*]azepine as donor in D–A photocatalysts

Katy Medrano-Uribe, Jorge Humbrías-Martín and Luca Dell'Amico

Beilstein J. Org. Chem. **2025,** *21,* 935–944. doi:10.3762/bjoc.21.76

Reactivity studies, general experimental procedures, product isolation and characterization, spectroscopic data for new compounds, and copies of NMR spectra

License and Terms: This is a supporting information file under the terms of the Creative Commons Attribution License (https://creativecommons.org/licenses/by/4.0). Please note that the reuse, redistribution and reproduction in particular requires that the author(s) and source are credited and that individual graphics may be subject to special legal provisions.
The license is subject to the *Beilstein Journal of Organic Chemistry* terms and conditions: (https://www.beilstein-journals.org/bjoc/terms)

Contents

A. General Information S3

B. Experimental setup for light irradiation Reaction setup with Kessil LED PR160L S5

C. Synthesis of the sulfur-based acceptors and tribenzo[b,d,f]azepine. S6

2-bromodibenzo[*b*,*d*]thiophene (**17**) S6

2-bromodibenzo[*b*,*d*]thiophene 5,5-dioxide (**18**) S6

(4-bromophenyl)(phenyl)sulfane (**19)** S6

1-bromo-4-(phenylsulfonyl)benzene (**20**) S6

9,9-dimethyl-9*H*-thioxanthene (**21**) S6

2-bromo-9,9-dimethyl-9*H*-thioxanthene (**22**) S7

2-bromo-9,9-dimethyl-9*H*-thioxanthene 10,10-dioxide (**23**) S7

tribenzo[*b*,*d*,*f*]azepine (**a**) S7

D. Synthesis of the D–A photocatalysts S8

General procedure A S8

9-(4-(phenylsulfonyl)phenyl)-9*H*-tribenzo[*b*,*d*,*f*]azepine (**4a**) S8

2-(9*H*-tribenzo[*b*,*d*,*f*]azepin-9-yl)dibenzo[*b*,*d*]thiophene 5,5-dioxide (**5a**) S8

9,9-dimethyl-2-(9*H*-tribenzo[*b*,*d*,*f*]azepin-9-yl)-9*H*-thioxanthene 10,10-dioxide (**6a**) S9

2-(5*H*-dibenzo[*b*,*f*]azepin-5-yl)dibenzo[*b*,*d*]thiophene 5,5-dioxide (**5b**) S9

2-(9*H*-carbazol-9-yl)dibenzo[*b*,*d*]thiophene 5,5-dioxide (**5c**) S9

2-(diphenylamino)dibenzo[*b*,*d*]thiophene 5,5-dioxide (**5d**) S10

2-(10*H*-phenoxazin-10-yl)dibenzo[*b*,*d*]thiophene 5,5-dioxide (**5e**) S10

E. Photoredox properties Luminescence quantum yield measurements. S11

Photoredox properties summary S11

9-(4-(phenylsulfonyl)phenyl)-9*H*-tribenzo[*b*,*d*,*f*]azepine (**4a**) S13

2-(9*H*-tribenzo[*b*,*d*,*f*]azepin-9-yl)dibenzo[*b*,*d*]thiophene 5,5-dioxide (**5a**) S16

9,9-dimethyl-2-(9*H*-tribenzo[*b*,*d*,*f*]azepin-9-yl)-9*H*-thioxanthene 10,10-dioxide (**6a**) S19

2-(5H-dibenzo[b,f]azepin-5-yl)dibenzo[b,d]thiophene 5,5-dioxide (**5b**) S22

2-(9*H*-carbazol-9-yl)dibenzo[*b*,*d*]thiophene 5,5-dioxide (**5c**) S25

2-(diphenylamino)dibenzo[*b*,*d*]thiophene 5,5-dioxide (**5d**) S28

2-(10*H*-phenoxazin-10-yl)dibenzo[*b*,*d*]thiophene 5,5-dioxide (**5e**) S31

F. Cyclic voltammetries of donors and acceptors S34
Oxidative quenching cycle study S36
General Procedure B S36
General Procedure C S37
Reductive quenching cycle study S38
General Procedure D S38
General Procedure E S39
I. Theoretical studies S40
J. References S49
K. NMR spectra S51

A. General Information

All reactions were carried out in anhydrous solvents purchased from commercial suppliers over molecular sieves in a sealed bottle which were used without further purification. Chemicals were purchased from commercial sources (Sigma-Aldrich, Fluorochem or TCI) and used without further purification. Organic solvents were purchased from Sigma-Aldrich. Reactions were monitored by thin-layer chromatography on silica gel 60F254, and/or by NMR spectroscopy.

NMR spectra were collected on a Bruker 400 Avance III HD spectrometer equipped with a BBI-z grad probehead, at the denoted spectrometer frequency given in MHz for the specified nucleus. Reported coupling constants and chemicals shifts were based on a first order analysis. The internal reference for ^{1}H NMR was the residual peak of $CDCl_3$ (7.26 ppm). The internal reference for ^{13}C NMR was the residual peak of $CDCl_3$ (77.16 ppm). All coupling constants (J) are reported in Hz with the following abbreviations: s = singlet, d = doublet, dd = double doublet, ddd = double of doublets of doublets, t = triplet, dt = double triplet, q = quadruplet, m = multiplet, br = broad.

Thin-layer chromatography (TLC) analysis was performed on pre-coated Merck TLC plates (silica gel 60G F254, 0.25 mm). Chromatographic purification of the products was accomplished using flash chromatography on silica gel (SiO_2, 0.04–0.0063 mm) purchased from Sigma-Aldrich, with the indicated solvent system according to the standard techniques, or with pre-coated Merck preparative TLC plates (silica gel 60G F254, 20 × 20 cm). Organic solutions were concentrated under reduced pressure on a Büchi rotary evaporator.

Steady-state absorption spectroscopy studies were performed at room temperature on a Varian Cary 50 UV-vis; 10 mm path length Hellma Analytics 100 QS quartz cuvettes were used.

Steady-state emission spectroscopy studies were performed at room temperature on a Varian Cary Eclipse Fluorescence spectrophotometer; 10 mm path length Hellma Analytics 117.100F QS quartz cuvettes were used.

Excited-state lifetimes measurements were performed at room temperature on a FLS1000 UV/Vis/NIR photoluminescence spectrometer (Edinburgh Instruments) at the PanLab facility of the Department of Chemical Sciences, University of Padova, founded by the MIUR-"Dipartimenti di Eccellenza" grant NExuS. The instrument was coupled with a high-speed detector with amplifier, operating in the spectral range: 230–850 nm and with a response time width.

The electrochemical characterizations were carried out in acetonitrile (MeCN)/0.1 M tetrabutylammonium hexafluorophosphate ($TBAPF_6$) at room temperature, on an BASi EC Epsilon potentiostat-galvanostat in a glass cell. All the cyclic voltammograms were recorded with a scan rate of 0.1 V/s. A typical three-electrode cell was employed, which was composed of a glassy carbon (GC) working electrode (3 mm diameter), a platinum wire as counter electrode and an Ag/AgCl electrode as reference electrode. The glass electrochemical cell was kept closed with a stopper annexed to the potentiostat. Oxygen was removed by purging the solvent with high-purity Nitrogen (N_2), introduced from a line into the cell by means of a glass pipe. The potential of ferrocenium/ferrocene (Fc^+/Fc) couple was used as internal reference system to calibrate the potentiostat. All the results were subsequently converted in V vs SCE, in agreement with the value reported in literature [$E_{1/2}(Fc^+/Fc)$ = +0.395 V vs SCE].

The IR compensation implemented within the potentiostat was used, and every effort was made throughout the experiments to minimize the resistance of the solution. The full electrochemical reversibility of the voltammetric wave of ferrocene was taken as an indicator of the absence of uncompensated resistance effects. The GC electrode was polished before any measurement with diamond paste, carefully rinsed with de-ionized water, ethanol, acetone and ultrasonically rinsed with a methanol/ethanol/acetone 1:1:1 (v/v) mixture for 5 minutes. After each series of CV experiments, the electrochemical cell was carefully rinsed with ethanol, acetone and de- ionized water; afterwards, the cell and the magnetic stirrer were sonicated for 5–10 min with acetone.

High-resolution mass spectrometry (HRMS) analyses were performed on MicroTOF-Q Bruker (ESI) and a GC Thermo Scientific Trace 1300 GC unit coupled to an APPI MasCom source mounted on a Thermo Scientific Exactive Plus EMR mass unit (Orbitrap FT-HRMS analyzer) or on a Xevo G2-XS QToF.

The Kessil lamps PR160L (50W) were purchased from Kessil webpage: https://kessil.com/products/science_PR160L.php

B. Experimental setup for light irradiation reaction setup with Kessil LED PR160L

Figure S1 shows the general setup of a batch reaction performed under Kessil LED PR160L light irradiation (400 nm). The reaction mixture is placed at a fixed distance (about 3 cm) from the light source and stirred vigorously. A maximum of three reaction vessels were irradiated at the same time, following the lines of homogeneous irradiance provided by the producer. To maintain a stable reaction temperature, one fan was placed above the irradiated vials. All the reactions were carried out using 100% intensity.

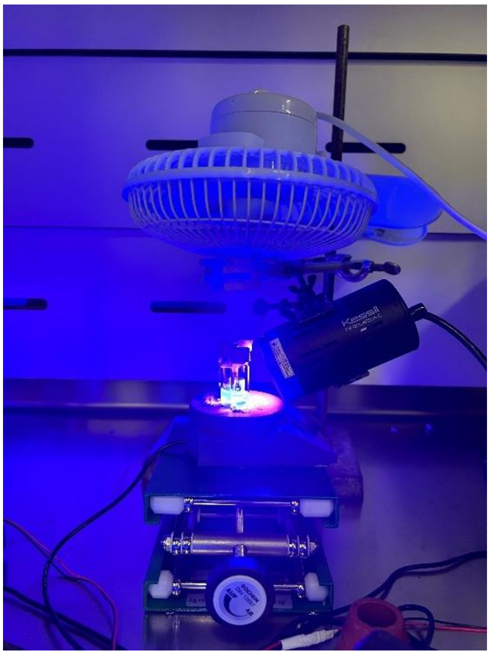

Figure S1: Reaction setup using Kessil lights

C. Synthesis of the sulfur-based acceptors and tribenzo[b,d,f]azepine.

2-Bromodibenzo[*b*,*d*]thiophene (**17**)

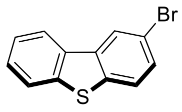

2-Bromodibenzo[*b*,*d*]thiophene (**17**) was synthesized following a reported procedure.[1] The spectral data matched with those reported in the literature.[2] **^{1}H NMR (400 MHz, $CDCl_3$)** δ 8.30 – 8.26 (m, 1H), 8.10 (ddd, *J* = 9.1, 6.3, 3.0 Hz, 1H), 7.91 – 7.83 (m, 1H), 7.72 (dd, *J* = 8.6, 6.4 Hz, 1H), 7.57 (ddd, *J* = 8.5, 3.4, 1.8 Hz, 1H), 7.52 – 7.48 (m, 2H). **^{13}C NMR (101 MHz, $CDCl_3$)** δ 140.01, 138.09, 137.34, 134.43, 129.57, 127.38, 124.67, 124.51, 124.09, 122.89, 121.77, 118.33. **HRMS (ESI+)**: m/z calcd. for $C_{12}H_8BrS$ [MH]+: 262.9525. Found: 262.9520.

2-Bromodibenzo[*b*,*d*]thiophene 5,5-dioxide (**18**)

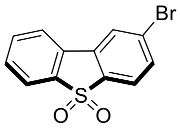

2-Bromodibenzo[*b*,*d*]thiophene 5,5-dioxide (**18**) was synthesized following a reported procedure.[3] The spectral data matched with those reported in the literature.[4]. **^{1}H NMR (400 MHz, $CDCl_3$)** δ 7.94 (s, 1H), 7.89 – 7.81 (m, 1H), 7.81 – 7.72 (m, 1H), 7.71 – 7.63 (m, 3H), 7.56 (dtd, *J* = 16.0, 7.5, 1.0 Hz, 1H). **^{13}C NMR (400 MHz, $CDCl_3$)** δ 138.1, 136.5, 134.1, 133.6, 133.3, 131.1, 130.3, 128.7, 125.0, 123.5, 122.4, 121.8. **HRMS (ESI+)**: m/z calcd. for $C_{12}H_8BrO_2S$ [MH]+: 294.9423. Found: 294.9426.

(4-Bromophenyl)(phenyl)sulfane (**19**)

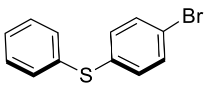

(4-Bromophenyl)(phenyl)sulfane (**19**) was synthesized following a reported procedure.[1] The spectral data matched with those reported in the literature.[5] **^{1}H NMR (400 MHz, $CDCl_3$)** δ 7.48 – 7.39 (m, 2H), 7.39 – 7.25 (m, 5H), 7.22 – 7.14 (m, 2H).**^{13}C NMR (101 MHz, $CDCl_3$)** δ 135.63, 134.95, 132.35, 132.21, 131.68, 129.50, 127.69, 120.99. **HRMS (ESI+)**: m/z calcd. for $C_{12}H_{10}BrS$ [MH]+: 264.9681. Found: 264.9686.

1-Bromo-4-(phenylsulfonyl)benzene (**20**)

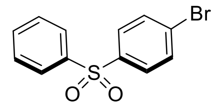

1-Bromo-4-(phenylsulfonyl)benzene (**20**) was synthesized following a reported procedure.[3] The spectral data matched with those reported in the literature.[6] **^{1}H NMR (400 MHz, $CDCl_3$)** δ 7.95 (d, *J* = 7.8 Hz, 2H), 7.83 (d, *J* = 8.2 Hz, 2H), 7.66 (d, *J* = 8.2 Hz, 2H), 7.61 (t, *J* = 7.3 Hz, 1H), 7.54 (t, *J* = 7.5 Hz, 2H**). ^{13}C NMR (101 MHz, $CDCl_3$)** δ 141.18, 140.71, 133.47, 132.62, 129.43, 129.22, 128.48, 127.67. **HRMS (ESI+)**: m/z calcd. for $C_{12}H_{10}BrO_2S$ [MH]+ : 296.9579. Found: 296.9574.

9,9-Dimethyl-9*H*-thioxanthene (**21**)

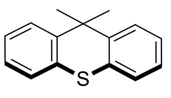

9,9-dimethyl-9*H*-thioxanthene (**21**) was synthesized following a reported procedure.[7] The chemical shifts obtained matched with those reported in the literature.[7] **^{1}H NMR (400 MHz, $CDCl_3$)** δ 7.60 (dd, *J* = 7.9, 1.5 Hz, 2H), 7.52 (dd,

J = 7.6, 1.6 Hz, 2H), 7.33 (td, *J* = 7.6, 1.5 Hz, 2H), 7.26 (td, *J* = 7.5, 1.4 Hz, 2H), 1.76 (s, 6H**).** **^{13}C NMR (101 MHz, $CDCl_3$)** δ 142.35, 132.96, 127.32, 126.69, 126.13, 124.72, 40.37, 25.20. **HRMS (ESI+)**: m/z calcd. for $C_{15}H_{15}S$ [MH]+: 227.0889. Found: 227.0887.

2-Bromo-9,9-dimethyl-9*H*-thioxanthene (**22**)

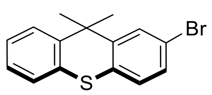

2-Bromo-9,9-dimethyl-9*H*-thioxanthene 10,10-dioxide (**22**) was synthesized following a reported procedure.[8] The compound was obtained in quantitative yield and used without further purification for the next step reaction. **^{1}H NMR (400 MHz, $CDCl_3$)** δ 7.65 (dd, *J* = 5.2, 1.9 Hz, 1H), 7.54 (ddd, *J* = 7.6, 5.5, 1.4 Hz, 1H), 7.46 (ddd, *J* = 7.5, 5.5, 1.5 Hz, 1H), 7.38 – 7.26 (m, 3H), 7.22 (tdd, *J* = 7.4, 5.1, 1.4 Hz, 1H), 1.69 (s, 6H). **^{13}C NMR (101 MHz, $CDCl_3$)** δ 144.52, 141.61, 132.35, 132.17, 128.94, 128.52, 127.94, 127.23, 126.86, 126.25, 126.01, 124.73, 120.49, 41.20, 24.76. **HRMS (ESI+)**: m/z calcd. for $C_{15}H_{14}BrS$ [MH]+: 304.9994. Found: 304.9990.

2-Bromo-9,9-dimethyl-9*H*-thioxanthene 10,10-dioxide (**23**)

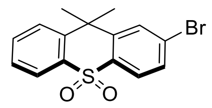

2-Bromo-9,9-dimethyl-9*H*-thioxanthene 10,10-dioxide (**23**) was synthesized following a reported procedure.[3] Purification by FC over silica gel (Hexane/AcOEt 70/30) afforded **23** in 68% yield. **^{1}H NMR (400 MHz, $CDCl_3$)** δ 8.20 (dd, *J* = 7.7, 1.5 Hz, 1H), 8.07 (t, *J* = 8.2 Hz, 1H), 7.90 (dd, *J* = 6.3, 1.7 Hz, 1H), 7.76 (dd, *J* = 8.1, 1.2 Hz, 1H), 7.71 – 7.59 (m, 2H), 7.54 (tt, *J* = 7.6, 2.8 Hz, 1H), 1.91 (s, 6H). **^{13}C NMR (101 MHz, $CDCl_3$)** δ 147.91, 145.22, 136.54, 135.92, 133.11, 130.75, 129.08, 127.84, 127.72, 125.97, 125.66, 124.37, 40.13, 30.37. **HRMS (ESI+)**: m/z calcd. for $C_{15}H_{14}BrO_2S$ [MH]+: 336.9892. Found: 336.9895.

Tribenzo[*b*,*d*,*f*]azepine **(a)**

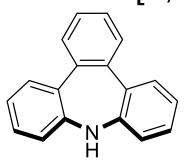

Tribenzo[*b*,*d*,*f*]azepine (**a**) was synthesized following a reported procedure.[9] The spectral data matched with those reported in the literature**.** **^{1}H NMR (400 MHz, $CDCl_3$)** δ 7.53 – 7.47 (m, 4H), 7.44 (dt, *J* = 5.8, 3.5 Hz, 2H), 7.23 (ddd, *J* = 9.3, 6.9, 3.1 Hz, 2H), 7.13 (td, *J* = 7.6, 1.4 Hz, 2H), 6.90 (dd, *J* = 7.8, 1.3 Hz, 2H), 5.20 (s, 1H). **^{13}C NMR (101 MHz, $CDCl_3$)** δ 151.10, 139.49, 132.79, 130.30, 130.24, 128.59, 127.89, 124.26, 119.87. **HRMS (ESI+)**: m/z calcd. for $C_{18}H_{14}N$[MH]+: 244.1121. Found: 244.1118.

D. Synthesis of the D–A photocatalysts

General procedure A

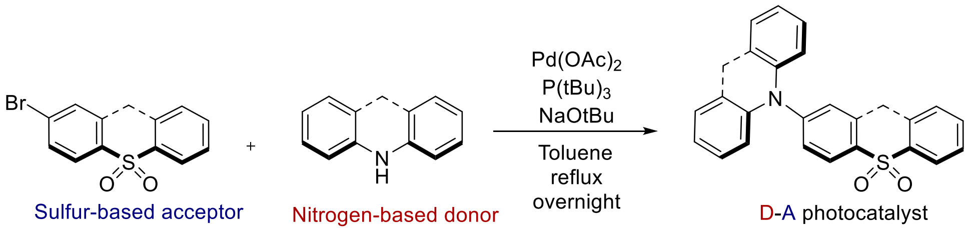

The procedure was modified from the one reported in the literature.[10] A 100 mL two-neck flask with a condenser and a Teflon-coated magnetic stir bar was charged with the sulfur-based acceptor (1 equiv), nitrogen-based donor (1.2–2.0 equiv), $Pd(OAc)_2$ (0.04 equiv), and NaO*t*-Bu (1.5-2.0 equiv). The reaction mixture was purged with Argon for 10 minutes. Then, toluene (0.025 M) was added, followed by the addition of $P(t\text{-}Bu)_3$ (0.08 equiv), and the reaction was purged with Argon 10 more minutes. The reaction was heated until reflux (120 °C, approx.) for 12 h. Upon completion, the reaction mixture was cooled at room temperature and poured into water and extracted with toluene three times . The combined organic layers were washed with water and dried over Na_2SO_4. After filtration and solvent evaporation purified by flash column chromatography (FC) over silica gel to furnish the target product.

9-(4-(Phenylsulfonyl)phenyl)-9*H*-tribenzo[*b*,*d*,*f*]azepine (**4a**)

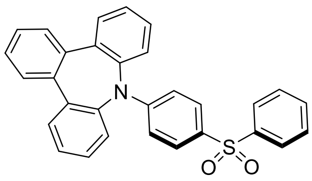

Synthesized following the general procedure starting from 1-bromo-4-(phenylsulfonyl)benzene (38.3 mg, 0.128 mmol, 1 equiv), Tribenzo[*b,d,f*]azepine (46.7 mg, 0.192 mmol, 1.2 equiv), $Pd(OAc)_2$ (1.12 mg ,0.005 mmol, 0.04 equiv), NaO*t*-Bu (18 mg, 0.193 mmol, 1.5 equiv) and $P(t\text{-}Bu)_3$ (2.6 ml, 0.010 mmol, 0.08 equiv). The reaction crude was purified by Flash column chromatography using hexane/EtOAc 9:1 as eluent. Compound **4a** was obtained in 36% yield. **Rf**= 0.2 (hexane/EtOAc, 8:2).**^{1}H NMR (400 MHz, $CDCl_3$)** δ 7.81 (dt, *J* = 7.1, 1.5 Hz, 2H), 7.71 – 7.65 (m, 2H), 7.59 (dd, *J* = 5.8, 3.5 Hz, 2H), 7.53 – 7.37 (m, 13H), 6.55 – 6.50 (m, 2H). **^{13}C NMR (101 MHz, $CDCl_3$)** δ 151.31, 144.52, 143.14, 138.64, 137.02, 132.50, 131.27, 129.61, 129.44, 129.31, 129.14, 128.87, 128.34, 128.28, 128.06, 127.21, 111.74. **HRMS (ESI+)**: m/z calcd. for $C_{30}H_{22}NO_2S$ $[MH]^+$: 460.1366. Found: 460.1364.

2-(9*H*-Tribenzo[*b*,*d*,*f*]azepin-9-yl)dibenzo[*b*,*d*]thiophene 5,5-dioxide (**5a**)

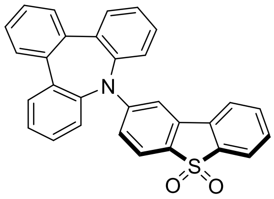

Synthesized following the general procedure starting from 2-bromodibenzo[*b*,*d*]thiophene 5,5-dioxide (160 mg, 0.544 mmol, 1 equiv), Tribenzo[*b,d,f*]azepine (158 mg, 0.652 mmol, 1.2 equiv), $Pd(OAc)_2$ (4 mg ,0.017 mmol, 0.04 equiv), NaO*t*-Bu (62 mg, 0.645 mmol, 1.5 equiv) and $P(t\text{-}Bu)_3$ (8.5 ml, 0.034 mmol, 0.08 equiv). The reaction crude was purified by Flash column chromatography using Hexane/AcOEt 9:1 as eluent. Compound **5a** was obtained in 45% yield. **Rf**= 0.15 (hexane/EtOAc, 8:2). **^{1}H NMR (400 MHz, $CDCl_3$)** δ 7.71 (ddt, *J* = 11.4, 7.4, 1.1 Hz, 3H), 7.63 (dd, *J* = 5.8, 3.4 Hz, 2H), 7.56 – 7.35 (m, 12H), 6.76 (d, *J* = 2.2 Hz, 1H), 6.53 (dd, *J* = 8.7, 2.2 Hz, 1H). **^{13}C NMR (101 MHz, $CDCl_3$)**152.49, 144.55, 139.23, 138.86, 136.95, 133.47, 133.33, 132.00, 131.48, 130.21,

129.71, 129.67, 128.65, 128.57, 128.37, 125.94, 123.32, 121.91, 121.50, 113.14, 103.58. **HRMS (ESI+)**: m/z calcd. for $C_{30}H_{20}NO_2S$ [MH]+ : 458.1209. Found: 458.1214.

9,9-Dimethyl-2-(9*H*-tribenzo[*b*,*d*,*f*]azepin-9-yl)-9*H*-thioxanthene 10,10-dioxide (**6a**)

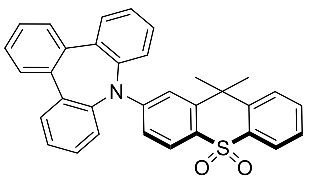

Synthesized following the general procedure starting from 2-bromo-9,9-dimethyl-9*H*-thioxanthene 10,10-dioxide (83.1 mg, 0.246 mmol, 1 equiv), Tribenzo*[b,d,f]*azepine (72 mg, 0.295 mmol, 1.2 equiv), $Pd(OAc)_2$ (2.2 mg ,0.001 mmol, 0.04 equiv), NaO*t*-Bu (35 mg, 0.369 mmol, 1.5 equiv) and P(*t*-Bu)$_3$ (5 ml, 0.002 mmol, 0.08 Equiv). The reaction crude was purified by Flash column chromatography using Hexane/AcOEt 9:1 as eluent. Compound **6a** was obtained in 68% yield. **Rf**= 0.22 (hexane/EtOAc, 8:2). **1H NMR (400 MHz, CDCl3)** δ 8.08 (dd, *J* = 7.7, 1.6 Hz, 1H), 7.72 (dd, *J* = 11.0, 8.2 Hz, 3H), 7.63 – 7.56 (m, 3H), 7.51 – 7.43 (m, 7H), 7.44 – 7.36 (m, 3H), 6.76 (d, *J* = 2.3 Hz, 1H), 6.53 (dd, *J* = 8.8, 2.3 Hz, 1H), 1.54 (s, 6H).**13C NMR (101 MHz, CDCl3)** δ 150.95, 147.13, 145.74, 144.59, 138.70, 137.59, 136.91, 132.19, 131.18, 129.41, 129.33, 128.22, 128.13, 127.17, 125.65, 125.36, 124.76, 123.72, 116.35, 110.38, 107.91, 39.16, 31.04. **HRMS (ESI+)**: m/z calcd. for $C_{33}H_{26}NO_2S$ [MH]+ : 500.1679. Found: 500.1673.

2-(5*H*-Dibenzo[*b*,*f*]azepin-5-yl)dibenzo[*b*,*d*]thiophene 5,5-dioxide (**5b**)

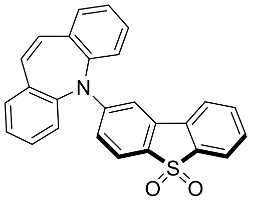

Synthesized following the general procedure starting from 2-bromo-9,9-dimethyl-9*H*-thioxanthene 10,10-dioxide (350 mg, 1.18 mmol, 1 equiv), and commercial 5H-Dibenz[*b,f*]azepine (342 mg, 1.77 mmol, 1.5 equiv), $Pd(OAc)_2$ (10.6 mg ,0.047 mmol, 0.04 equiv), NaO*t*-Bu (170 mg, 1.77 mmol, 1.5 equiv) and P(*t*-Bu)$_3$ (23 ml, 0.019 mmol, 0.08 Equiv). The reaction crude was purified by Flash column chromatography using hexane/EtOAc 75:25 as eluent. Compound **5b** was obtained in 54% yield. **Rf**= 0.16 (hexane/EtOAc, 8:2). **^{1}H NMR (400 MHz, $CDCl_3$)** δ 7.73 (dd, *J* = 7.4, 1.4 Hz, 1H), 7.57 (td, *J* = 7.4, 6.9, 1.7 Hz, 2H), 7.51 (dt, *J* = 7.7, 1.6 Hz, 4H), 7.48 – 7.39 (m, 5H), 7.36 (dd, *J* = 7.2, 1.3 Hz, 1H), 6.88 (s, 2H), 6.53 (d, *J* = 2.2 Hz, 1H), 6.32 (dd, *J* = 8.7, 2.2 Hz, 1H). **^{13}C NMR (101 MHz, $CDCl_3$)** δ 154.34, 142.22, 139.33, 136.44, 134.22, 133.74, 132.10, 131.38, 131.18, 130.87, 130.72, 130.20, 128.67, 126.97, 123.62, 122.44, 121.91, 113.78, 104.30. **HRMS (ESI+)**: m/z calcd. for $C_{26}H_{18}NO_2S$ [MH]+ : 408.1053. Found: 408.1050.

2-(9*H*-Carbazol-9-yl)dibenzo[*b*,*d*]thiophene 5,5-dioxide (**5c**)

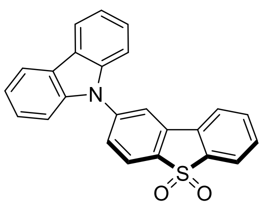

Synthesized following the general procedure starting from 2-bromo-9,9-dimethyl-9*H*-thioxanthene 10,10-dioxide (350 mg, 1.18 mmol, 1 equiv), and commercial carbazole (396 mg, 2.37 mmol, 2 equiv), $Pd(OAc)_2$ (10.6 mg ,0.047 mmol, 0.04 equiv), NaO*t*-Bu (170 mg, 1.77 mmol, 1.5 equiv) and P(*t*-Bu)$_3$ (23 ml, 0.019 mmol, 0.08 Equiv). The reaction crude was purified by Flash column chromatography using hexane/EtOAc 7:3 as eluent. Compound **5c** was obtained in 64 % yield. **Rf**= 0.2 (Hexane:EtOAc, 8:2). **^{1}H NMR (400 MHz, $CDCl_3$)** δ 8.20-8.14 (m, 2H), 8.07 (dd, *J* = 8.1, 1.6 Hz, 1H), 8.02 (d, *J* = 1.7 Hz, 1H), 7.91 (d, *J* = 7.6 Hz, 1H), 7.82 – 7.75 (m, 2H), 7.68 (tt, *J* = 7.6, 1.4 Hz, 1H), 7.61 (tt, *J* = 7.5, 1.3 Hz, 1H), 7.49 – 7.43 (m, 4H), 7.36 (ddt, *J* = 7.8, 5.4, 1.8 Hz, 2H).**^{13}C NMR (101 MHz, $CDCl_3$)** δ 143.53, 140.29, 138.46, 135.92,134.22, 131.22, 130.94, 128.61, 126.63, 124.13, 122.59, 122.02. 121.15, 120.79, 119.92, 109.68. **HRMS (ESI+)**: m/z calcd. for $C_{24}H_{16}NO_2S$ [MH]+ : 382.0896. Found: 382.0899.

2-(Diphenylamino)dibenzo[*b*,*d*]thiophene 5,5-dioxide **5d**

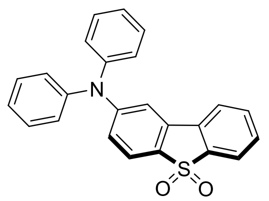

Synthesized following the general procedure starting from 2-bromo-9,9-dimethyl-9*H*-thioxanthene 10,10-dioxide (443 mg, 1.5 mmol, 1 equiv), and commercial diphenylamine (305 mg, 1.8 mmol, 1.2 equiv), $Pd(OAc)_2$ (13.5 mg ,0.06 mmol, 0.04 equiv), NaO*t*-Bu (216 mg, 2.25 mmol, 1.5 equiv) and $P(t\text{-}Bu)_3$ (29.6 ml, 0.12 mmol, 0.08 Equiv). The reaction crude was purified by Flash column chromatography using hexane/EtOAc 7:3 as eluent. Compound **5d** was obtained in 55 % yield. **Rf**= 0.18 (hexane/EtOAc, 8:2). **^{1}H NMR (400 MHz, $CDCl_3$)** δ 7.82 (d, *J* = 7.4 Hz, 1H), 7.62 (d, *J* = 8.5 Hz, 1H), 7.56 (d, *J* = 4.3 Hz, 2H), 7.52 (dq, *J* = 8.2, 4.3, 3.9 Hz, 1H), 7.38 (t, *J* = 7.7 Hz, 4H), 7.33 (t, *J* = 1.7 Hz, 1H), 7.20 (m, 6H), 7.05 (dt, *J* = 8.7, 1.8 Hz, 1H). **^{13}C NMR (101 MHz, $CDCl_3$)** δ 153.40, 146.36, 138.88, 133.55, 133.50, 131.67, 130.41, 130.00, 128.78, 126.13, 125.24, 123.36, 122.07, 121.79, 121.62, 112.52. **HRMS (ESI+)**: m/z calcd. for $C_{24}H_{18}NO_2S$ [MH]+ : 384.1053. Found: 384.1057.

2-(10*H*-Phenoxazin-10-yl)dibenzo[*b*,*d*]thiophene 5,5-dioxide (**5e**)

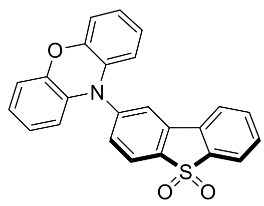

Synthesized following the general procedure starting from 2-bromo-9,9-dimethyl-9*H*-thioxanthene 10,10-dioxide (350 mg, 1.18 mmol, 1 equiv), and commercial phenoxazine (434 mg, 2.37 mmol, 2 equiv), $Pd(OAc)_2$ (10.6 mg ,0.047 mmol, 0.04 equiv), NaO*t*-Bu (170 mg, 1.77 mmol, 1.5 equiv) and $P(t\text{-}Bu)_3$ (23 ml, 0.019 mmol, 0.08 Equiv). The reaction crude was purified by Flash column chromatography using Hexane/AcOEt 8:2 as eluent. Compound **5d** was obtained in 71 % yield. **Rf**= 0.21 (hexane/EtOAc, 8:2). The chemical shifts obtained matched with those reported in the literature.[11] **^{1}H NMR (400 MHz, $CDCl_3$)** δ ^{1}H NMR (400 MHz, $CDCl_3$) δ 8.08 (dd, *J* = 15.6, 8.0 Hz, 1H), 7.89 (d, *J* = 7.5 Hz, 1H), 7.84 – 7.74 (m, 2H), 7.67 (t, *J* = 7.5 Hz, 1H), 7.65 – 7.50 (m, 2H), 6.74 (qd, *J* = 7.9, 4.2 Hz, 4H), 6.69 – 6.58 (m, 2H), 6.02 – 5.98 (m, 2H). **^{13}C NMR (101 MHz, $CDCl_3$)** δ 145.75, 144.78, 144.71, 138.76, 138.00, 136.00, 134.89, 134.08, 133.96, 131.84, 131.34, 125.75, 125.18, 124.21, 123.24, 123.21, 123.07, 122.76, 116.80, 116.71, 114.26, 114.14. **HRMS (ESI+)**: m/z calcd. for $C_{24}H_{16}NO_3S$ [MH]+ : 398.0845. Found: 398.0847.

E. Photoredox properties luminescence quantum yield measurements.

The luminescence quantum yield measurements were performed with quinine sulfate 1 × 10^{-5} M in H_2SO_4 0.5M (Φ = 0.54) for compounds **5a, 5c and 5d**, while for compounds **4a**, **6a**, **5b** and **5e** a solution 5 × 10^{-6} M of the standard was used.

$\Phi_x = \Phi_{st} \times (I_x / I_{st}) \times (f_{st}/f_x) \times (\eta^2{}_x / \eta^2{}_s)$

I is the measured integrated fluorescence emission intensity, f is the absorption factor, η is the refractive index of the solvent and Φ is the quantum yield. The index x denotes the sample and the index *st* denotes the standard. [12]

Redox potential of the excited state

The oxidation potential of the excited state photocatalysts E^*ox was estimated by means of the Rehm–Weller formula:

$E^*{}_{ox} = E_{ox} - E_{0,0}$

$E^*{}_{red} = E_{0,0} + E_{red}$

The oxidation potential E_{ox} and E_{red} were determined by cyclic voltammetry measurements and the excitation energy $E_{0,0}$ was determined from the intersection point between the absorption and the emission profiles.[13]

Photoredox properties summary

Acceptor analysis

PC	λ_{max} abs (nm)	λ_{max} em (nm)	$E_{0,0}$ (eV)	τ(ns)	Stokes shift (nm)	QY (%)	E_{ox} (V)	E^*_{ox} (V)	E_{red} (V)	E^*_{red} (V)
**4a**	308	400 , 478	3.7	τ_{avInt}= 0.9	81	12	1.46	-2.24	-2.35	1.35
**5a**	320	430	3.3	τ_{avInt}= 2.2	110	7	1.41	-1.89	-1.95	1.35
**6a**	292	398	3.7	τ_{avInt}= 2.0	106	10	1.43	-2.27	-2.4	1.3

Table S1: All potentials in V vs SCE. Measurements were performed in MeCN at room temperature.

Donor analysis

PC	λ_{max} abs (nm)	λ_{max} em (nm)	$E_{0,0}$ (eV)	τ(ns)	Stokes shift (nm)	QY (%)	E_{ox} (V)	E^*_{ox} (V)	E_{red} (V)	E^*_{red} (V)
**5a**	320	430	3.3	$\tau_{av=}$2.2	110	7	1.41	-1.89	-1.95	1.35
**5b**	312	441	3.2	0.7	129	6	1.32	-1.88	-1.96	1.24
**5c**	336	493	3.1	11.6	157	14	1.42	-1.68	-1.75	1.35
**5d**	346	525	2.9	9.1	179	14	1.12	-1.78	-1.86	1.04
**5e**	393	564	2.6	3.9	171	16	0.75	-1.85	-1.74	0.86

Table S2: All potentials in V vs SCE. Measurements were performed in MeCN at room temperature.

9-(4-(Phenylsulfonyl)phenyl)-9*H*-tribenzo[*b*,*d*,*f*]azepine (**4a**)

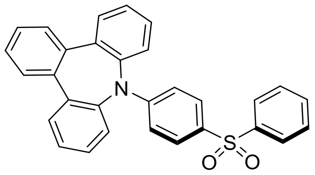

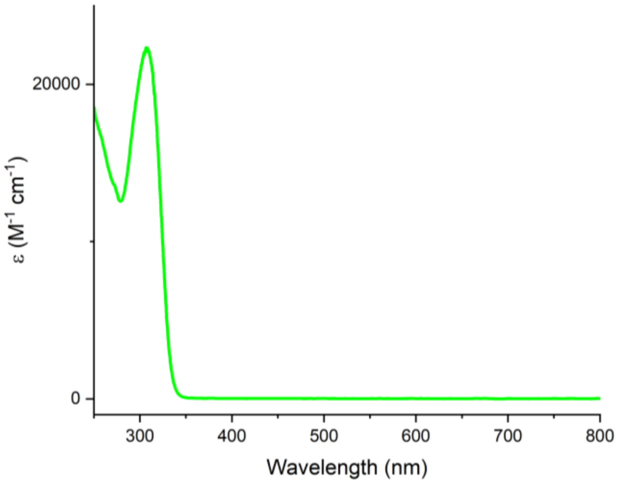

Concentration (mmol/mL)	
(green line)	$1\cdot10^{-4}$
λ peak	308 nm

*Figure S2: UV–vis absorption of **4a** at $1\cdot10^{-4}$ M (green trace). Recorded in MeCN in quartz cuvettes (1 cm optical path).*

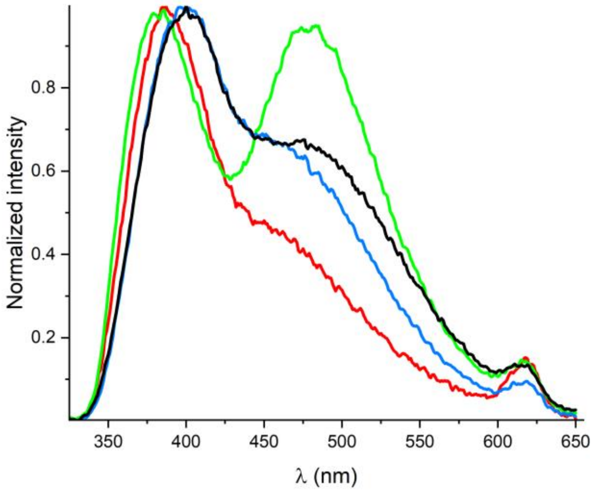

$\lambda_{excitation}$ = 308 nm

	Solvent	Peaks (nm)
(black line)	MeCN	400, 478
(blue line)	DMF	400, 448
(green line)	DCM	383, 477
(red line)	THF	385, 451

*Figure S3: Normalized fluorescence emission of **4a** $1\cdot10^{3}$ M in different solvents. MeCN (black trace); DMF (blue trace); DCM (green trace); THF (red trace).* $\lambda_{excitation}$ = 308 nm.

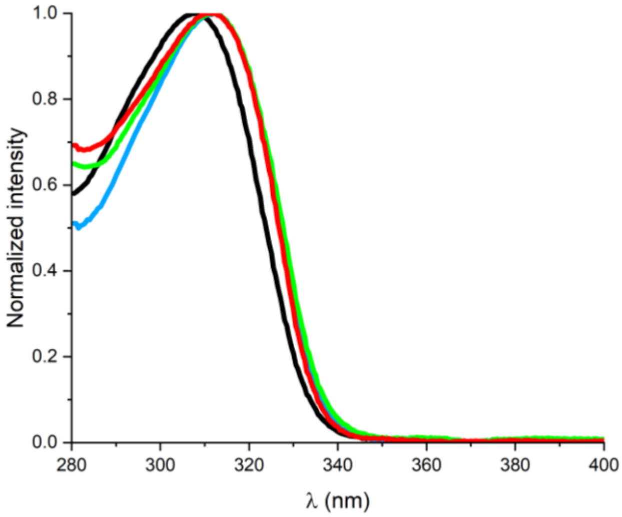

	Solvent	Peak (nm)
(black)	MeCN	319
(blue)	DMF	323
(green)	DCM	323
(red)	THF	323

Figure S4: Normalized absorption profile of ***4a*** *$1{\cdot}10^{-5}$ M in different solvents. MeCN (black trace); DMF (blue trace); DCM (green trace); THF (red trace).*

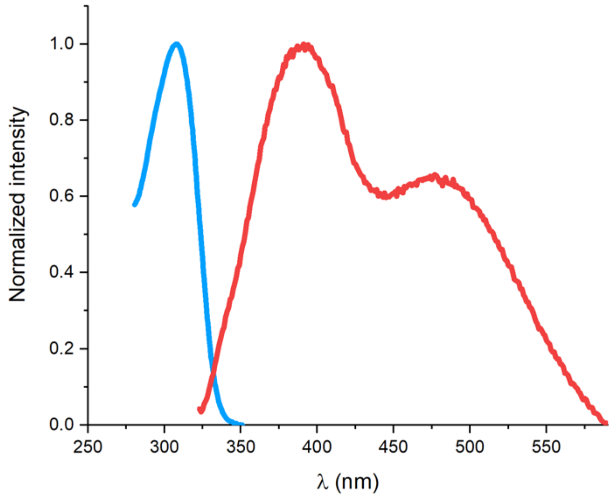

Absorption in MeCN
Emission in MeCN

$\lambda_{excitation}$	=	308 nm
$\lambda_{intersection}$	=	3.7 eV
Stokes shift	=	82 nm

Figure S5: Normalized optical absorption spectrum (blue trace) and emission spectra (red trace) of ***4a****.* $\lambda_{excitation}$ = 308 nm.

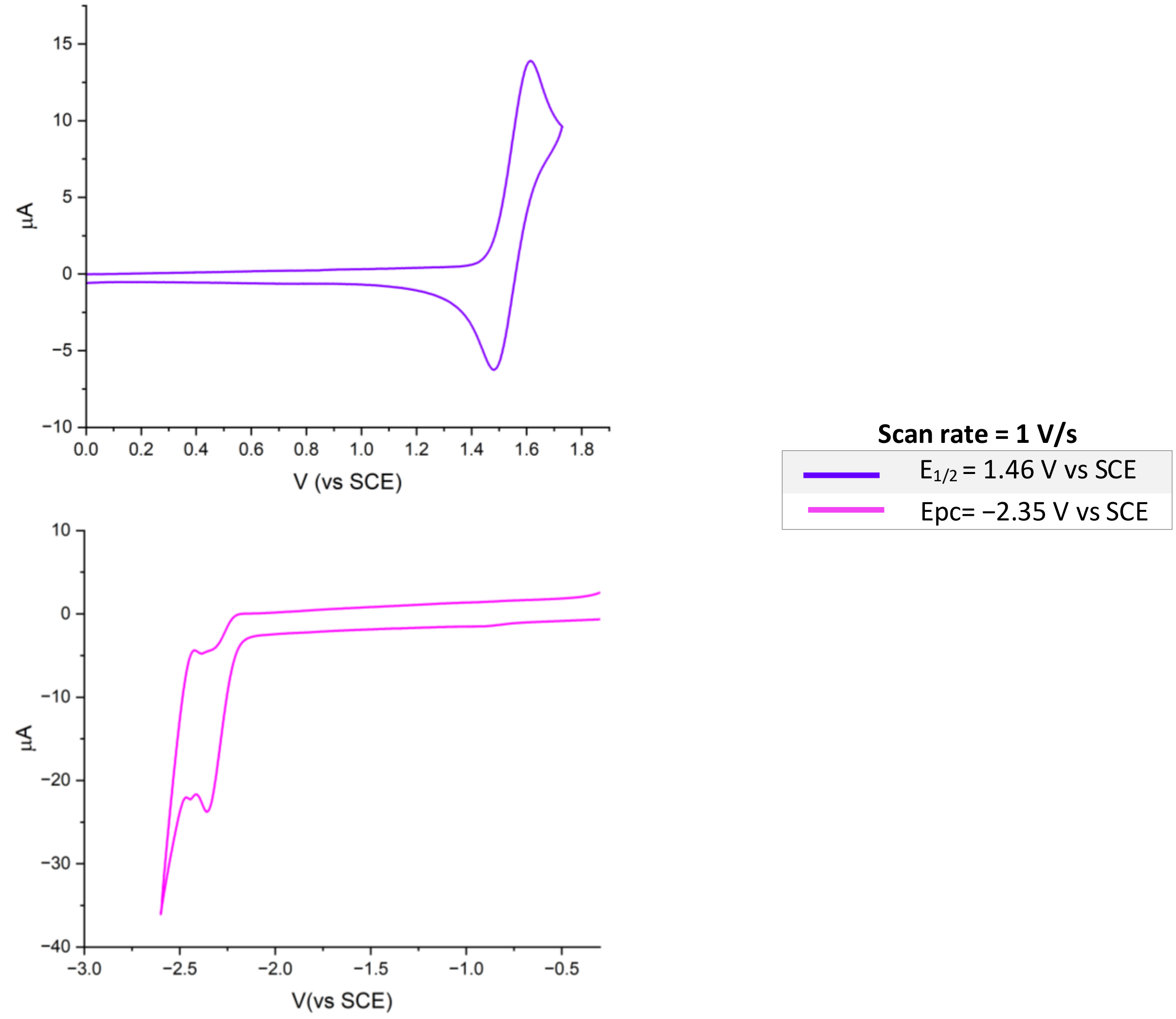

*Figure S6: Cyclic voltammograms of **4a** 1 × 10⁻³ M. Recorded with GC as the working electrode in MeCN with 0.1 M [Bu_4N][PF_6] as the supporting electrolyte at 1 V/s scan rate. Reversibility and irreversibility were determined using different scan rates.*

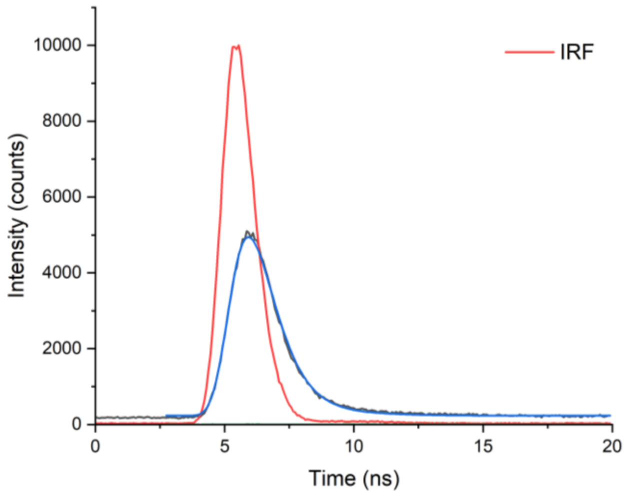

*Figure S7: Time-resolved emission decay (excitation at 340 nm) of **4a** $1{\cdot}10^{-5}$ M in MeCN solution measured by TC-SPC (τ = 0.9 ns), from deconvolution and single-exponential fitting, X2 = 3.88).*

2-(9*H*-Tribenzo[*b*,*d*,*f*]azepin-9-yl)dibenzo[*b*,*d*]thiophene 5,5-dioxide (**5a**)

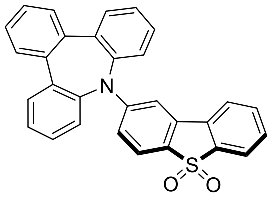

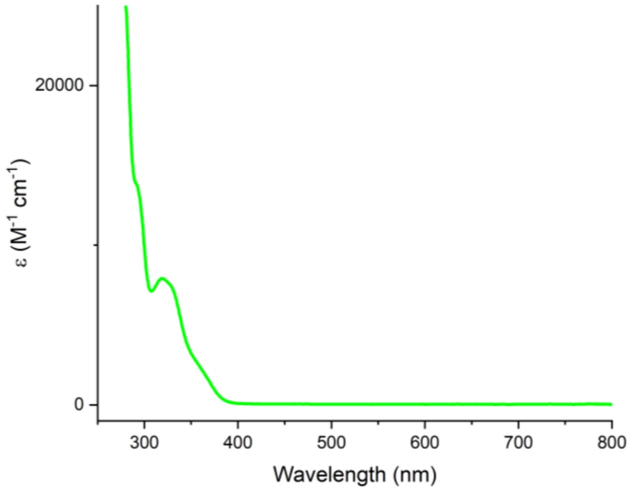

Concentration (mmol/mL)	
(green line)	*1·10⁻⁴*
λ peak 320 nm	

*Figure S8: UV–vis absorption of **5a** at $1{\cdot}10^{-4}$ M. Recorded in MeCN in quartz cuvettes (1 cm optical path).*

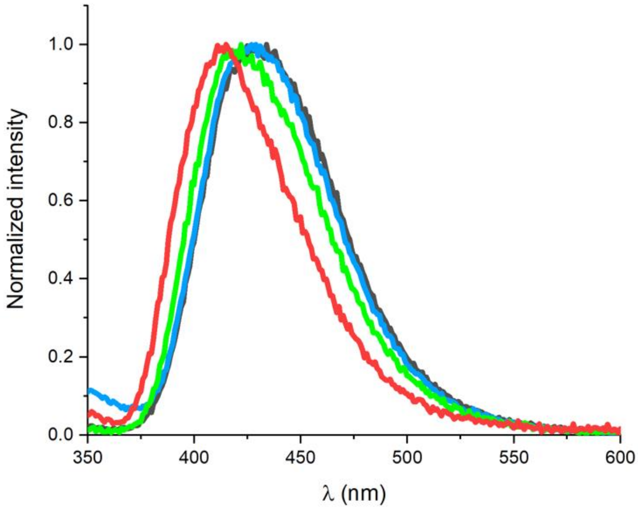

$\lambda_{excitation}$ = 320 nm

	Solvent	Peak (nm)
(black line)	MeCN	430
(blue line)	DMF	430
(green line)	DCM	420
(red line)	THF	413

*Figure S9: Normalized fluorescence emission of **5a** $1{\cdot}10^{-5}$ M in different solvents. MeCN (black trace); DMF (blue trace); DCM (green trace); THF (red trace).* $\lambda_{excitation}$ = 320 nm.

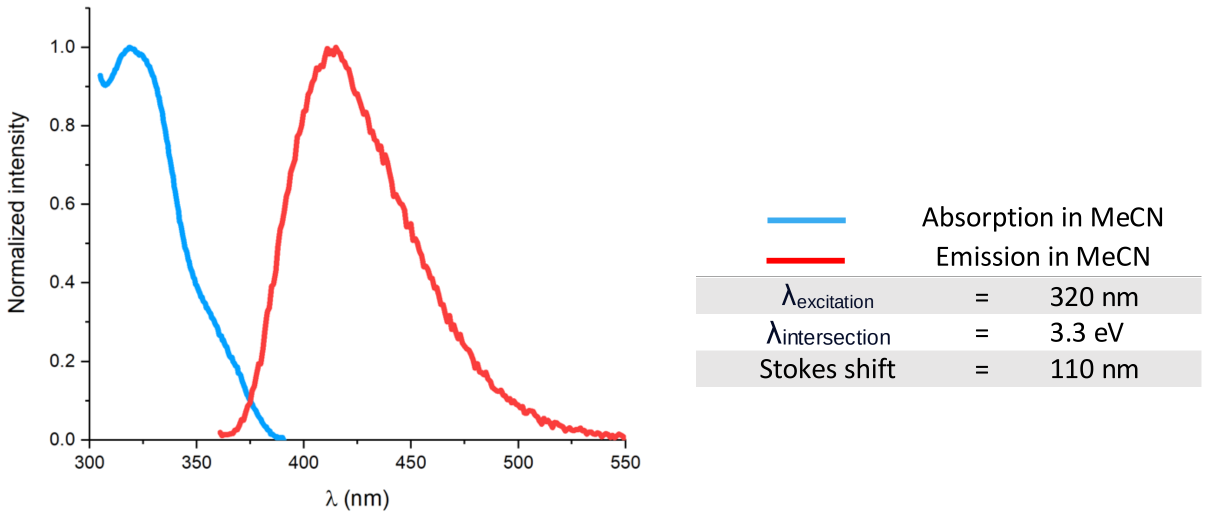

Figure S10: Normalized optical absorption spectrum (blue trace) and emission spectra (red trace) of ***5a****.* $\lambda_{excitation}$ = 320 nm

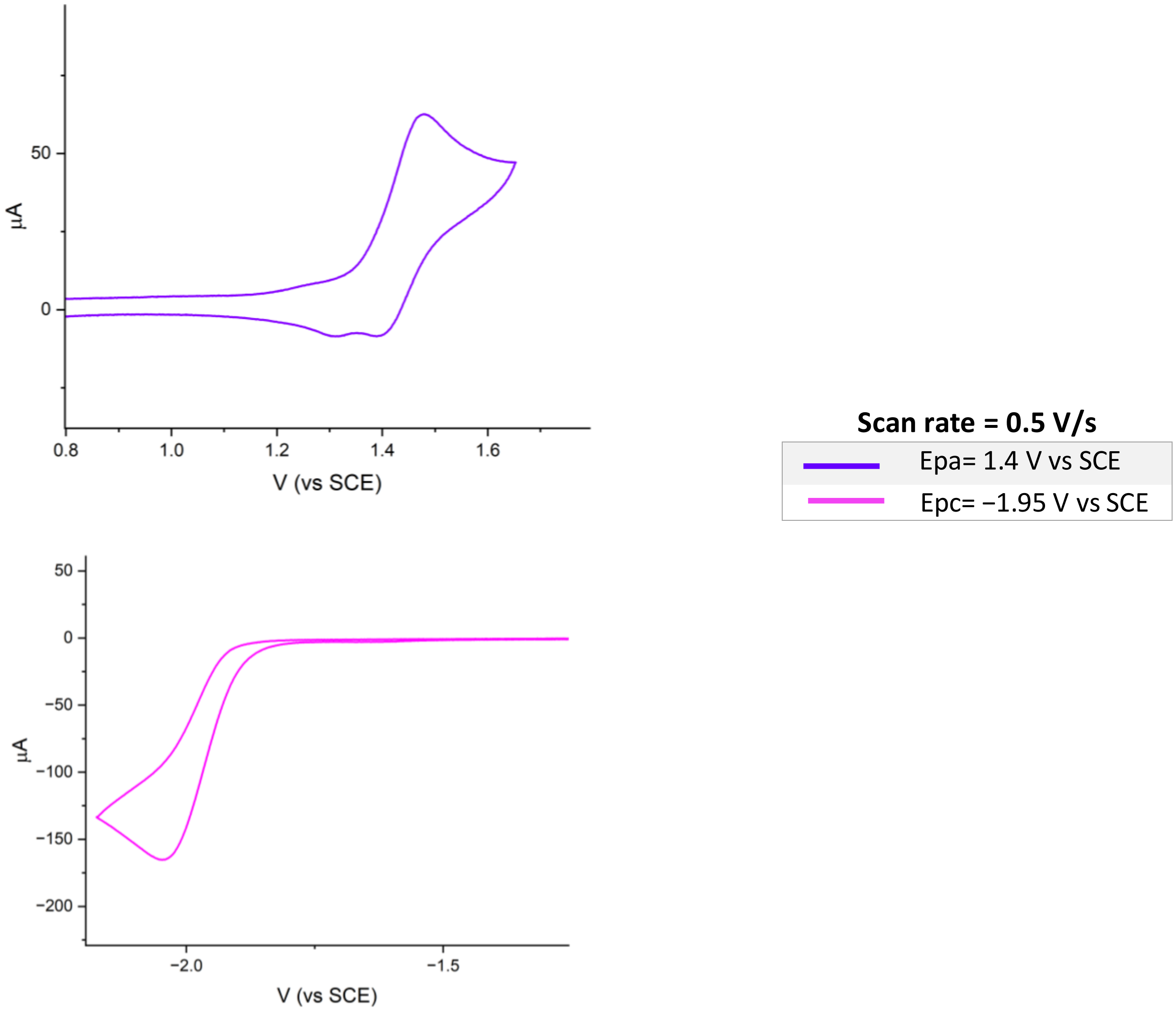

Figure S11: Cyclic voltammograms of ***5a*** *10^{-3} M. Recorded with GC as the working electrode in MeCN with 0.1 M [Bu_4N][PF_6] as the supporting electrolyte at 0.5 V/s scan rate. Irreversibility were determined by using different scan rates.*

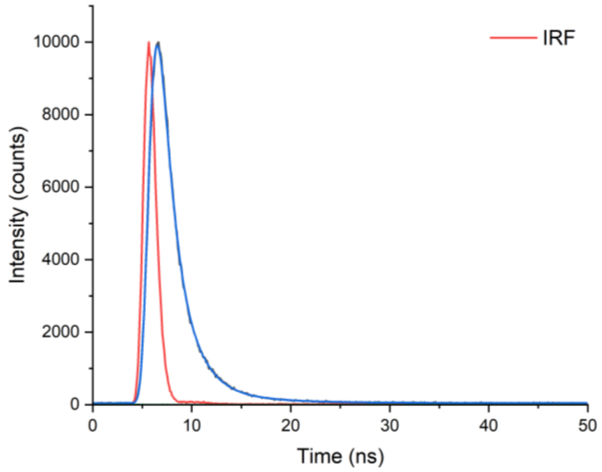

*Figure S12: Time-resolved emission decay (excitation at 340 nm) of **5a** $1 \cdot 10^{-5}$ M in MeCN solution measured by TC-SPC (τAvInt= 2.22 ns), from deconvolution and single-exponential fitting, X2 = 1.23).*

9,9-Dimethyl-2-(9*H*-tribenzo[*b*,*d*,*f*]azepin-9-yl)-9*H*-thioxanthene 10,10-dioxide (**6a**)

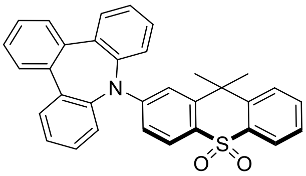

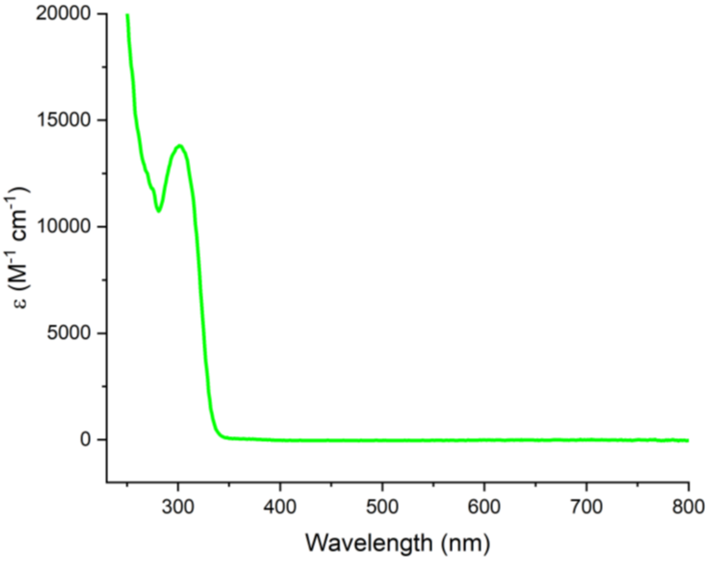

Concentration (mmol/mL)	
(green line)	*1·10⁻⁴*
λ peak	292 nm

Figure S13: UV–vis absorption of ***6a*** *at 1·10^{-4} M. Recorded in MeCN in quartz cuvettes (1 cm optical path).*

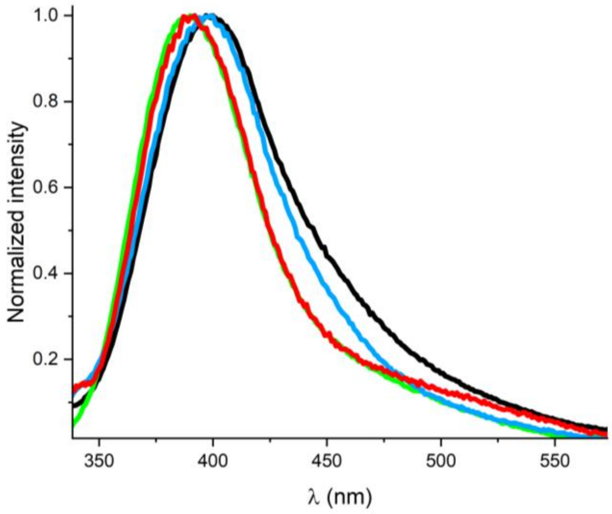

$\lambda_{excitation}$ = 310 nm

	Solvent	Peak (nm)
(black line)	MeCN	398
(blue line)	DMF	396
(green line)	DCM	389
(red line)	THF	389

Figure S14: Normalized fluorescence emission of ***6a*** *1·10^{-5} M in different solvents. MeCN (black trace); DMF (blue trace); DCM (green trace); THF (red trace).* $\lambda_{excitation}$ = 310 nm.

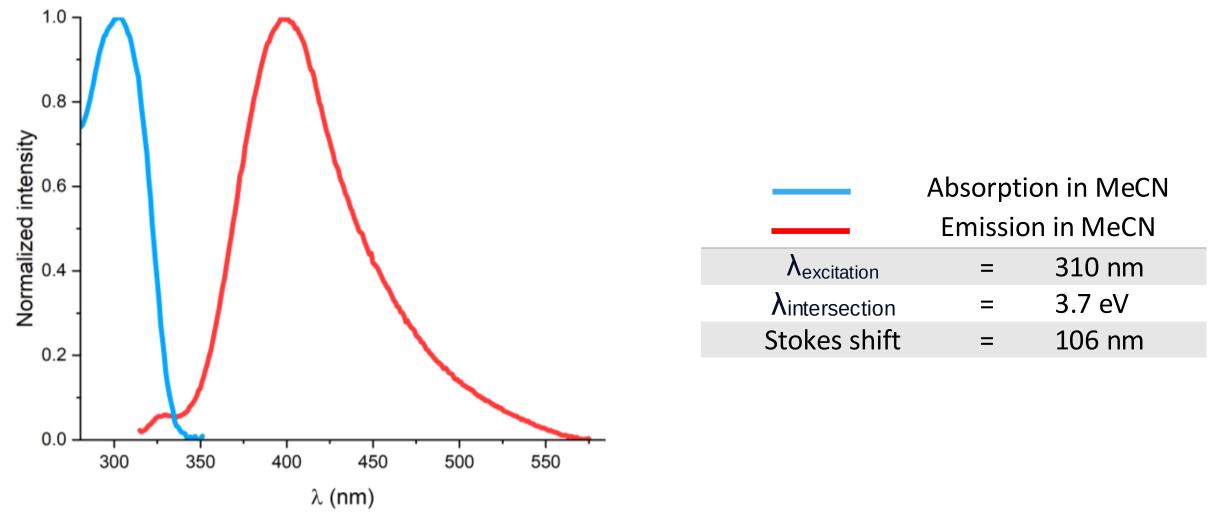

*Figure S15: Normalized optical absorption spectrum (blue trace) and emission spectra (red trace) of **6a**.* $\lambda_{excitation}$ = 310 nm.

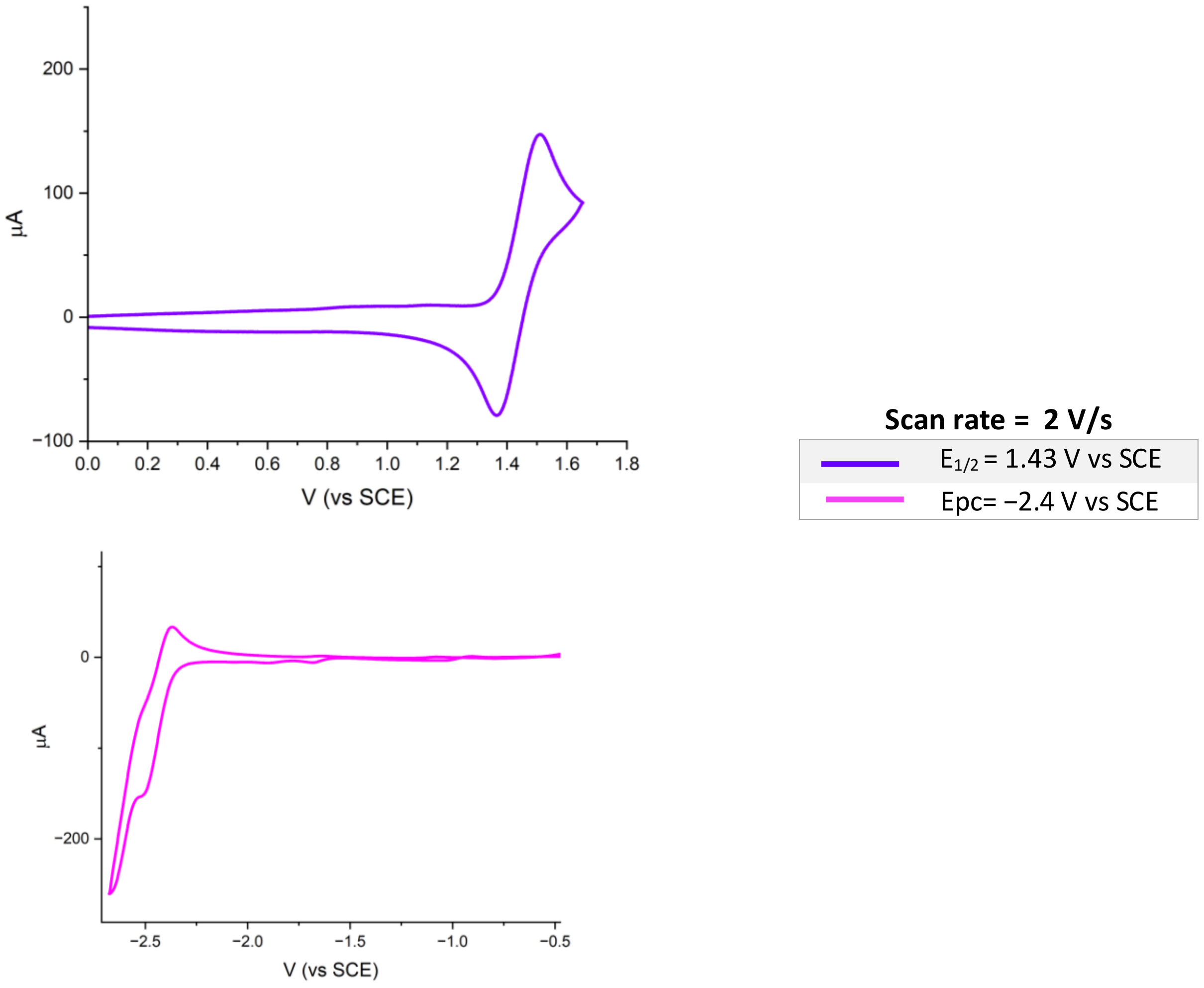

*Figure S16: Cyclic voltammograms of **6a** 10^{-3} M. Recorded with GC as the working electrode in MeCN with 0.1 M [Bu_4N][PF_6] as the supporting electrolyte at 2 V/s scan rate. Reversibility and irreversibility were determined by using different scan rates.*

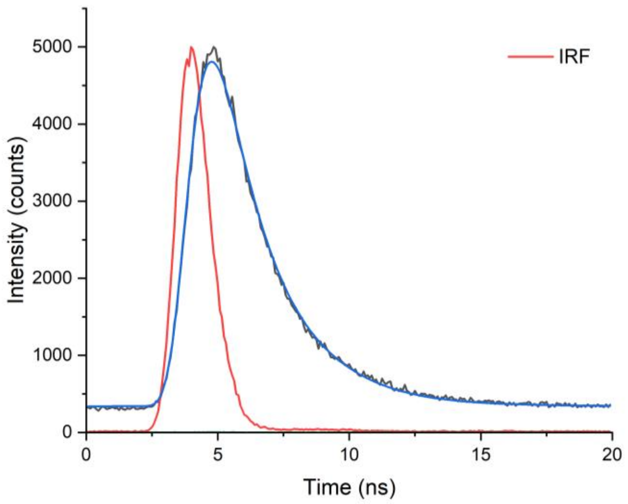

*Figure S17: Time-resolved emission decay (excitation at 340 nm) of **6a** $1{\cdot}10^{-5}$ M in MeCN solution measured by TC-SPC (τ=2.03), from deconvolution and single-exponential fitting, X2 = 1.69).*

2-(5*H*-Dibenzo[*b*,*f*]azepin-5-yl)dibenzo[b,d]thiophene 5,5-dioxide (**5b**)

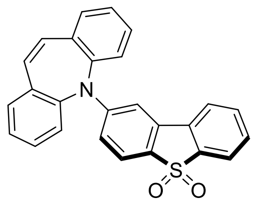

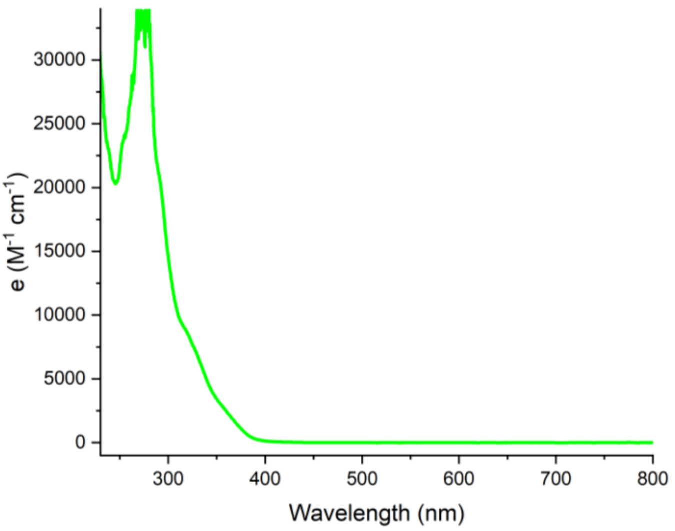

Concentration (mmol/mL)	
(green trace)	*1·10⁻⁴*
λ peak	312 nm

*Figure S18: UV–vis absorption of **5b** at different concentrations 1·10^{-4} M (green trace). Recorded in MeCN in quartz cuvettes (1 cm optical path).*

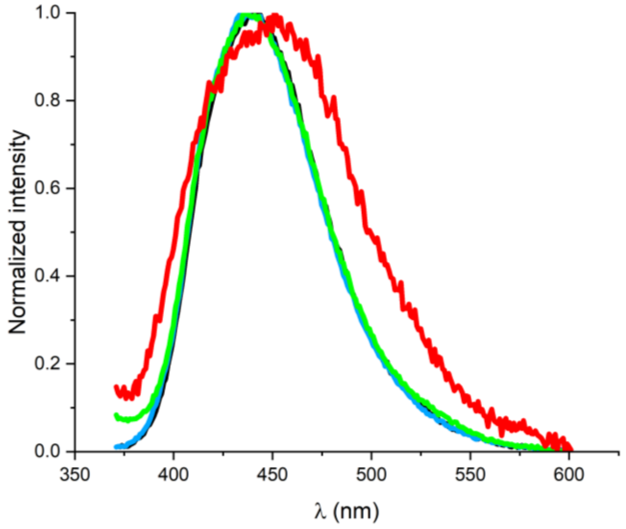

$\lambda_{excitation}$ = 312 nm

	Solvent	Peak (nm)
(black trace)	MeCN	441
(blue trace)	DMF	438
(green trace)	DCM	438
(red trace)	THF	450

*Figure S19: Normalized fluorescence emission of **5b** 1·10^{-5} M in different solvents. MeCN (black trace); DMF (blue trace); DCM (green trace); THF (red trace).* $\lambda_{excitation}$ = 312 nm

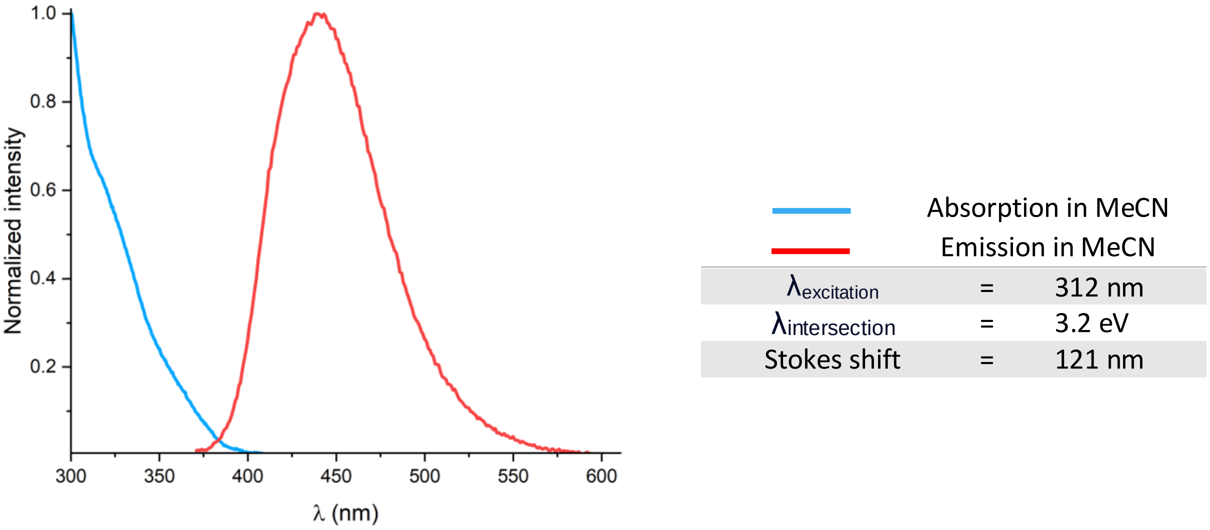

*Figure S20: Normalized optical absorption spectrum (blue trace) and emission spectra (red trace) of **5b**.* $\lambda_{excitation}$ = 312 nm.

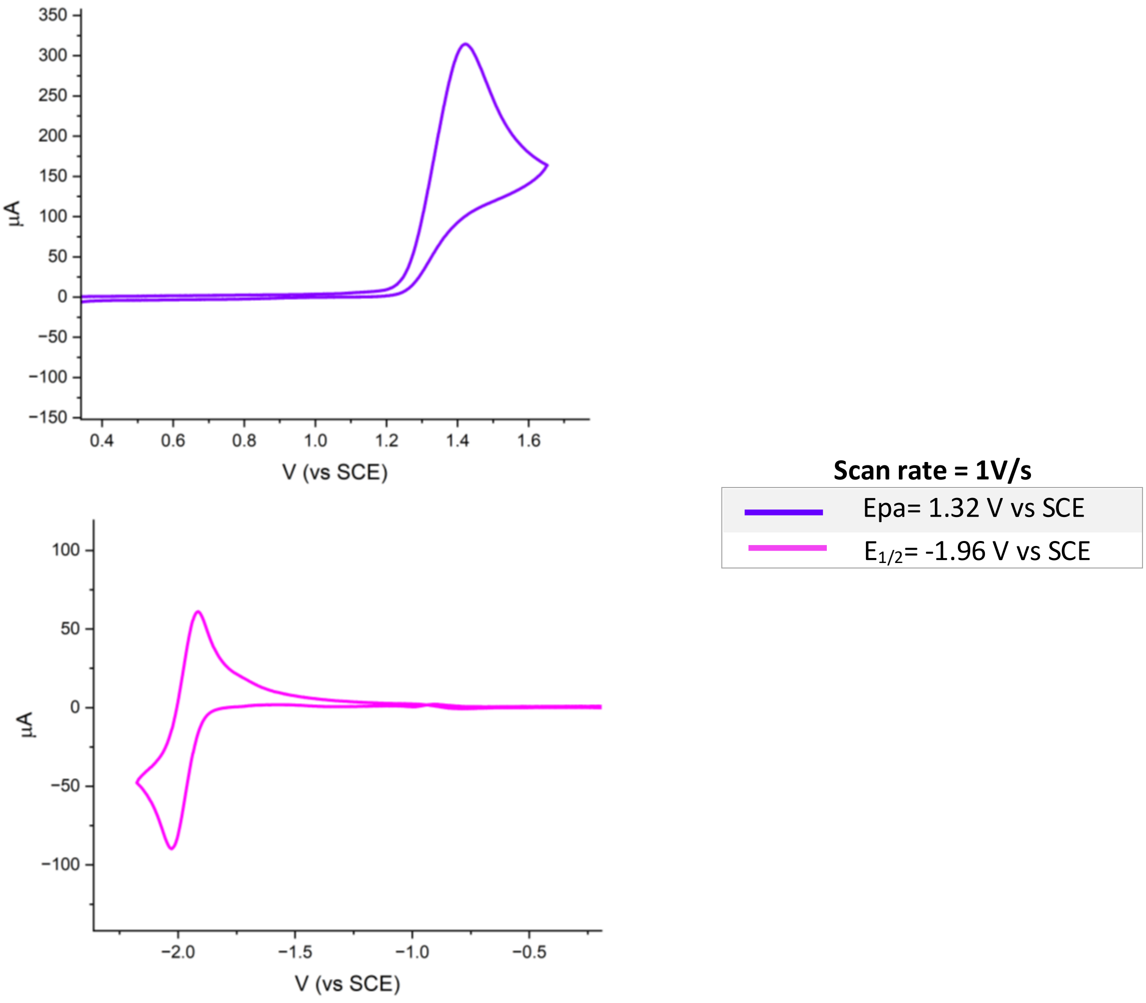

*Figure S21: Cyclic voltammograms of **5b** 10^{-3} M. Recorded with GC as the working electrode in MeCN with 0.1 M $[Bu_4N][PF_6]$ as the supporting electrolyte at 1 V/s scan rate. Reversibility and irreversibility were determined by using different scan rates.*

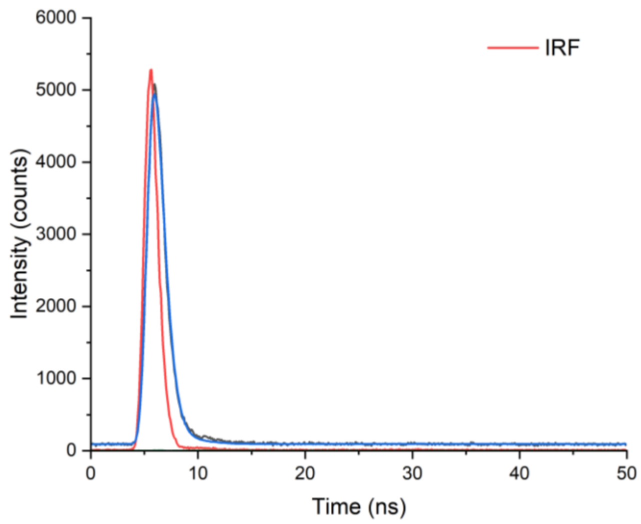

Figure S22: Time-resolved emission decay (excitation at 340 nm) of ***5b*** *1·10^{-5} M in MeCN solution measured by TC-SPC (τ = 0.7 ns), from deconvolution and single-exponential fitting, X2 = 1.96).*

2-(9*H*-Carbazol-9-yl)dibenzo[*b*,*d*]thiophene 5,5-dioxide (**5c**)

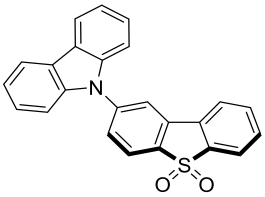

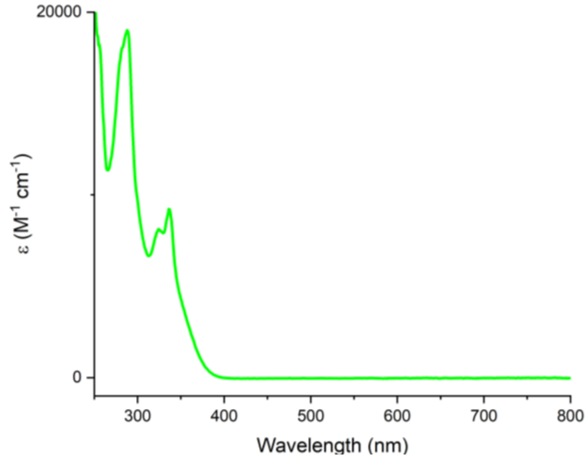

Concentration (mmol/mL)	
(green line)	*1·10^–4*
λ peak	336 nm

Figure S23: UV–vis absorption of ***5c*** *at 1·10^–4^ M (green trace). Recorded in MeCN in quartz cuvettes (1 cm optical path).*

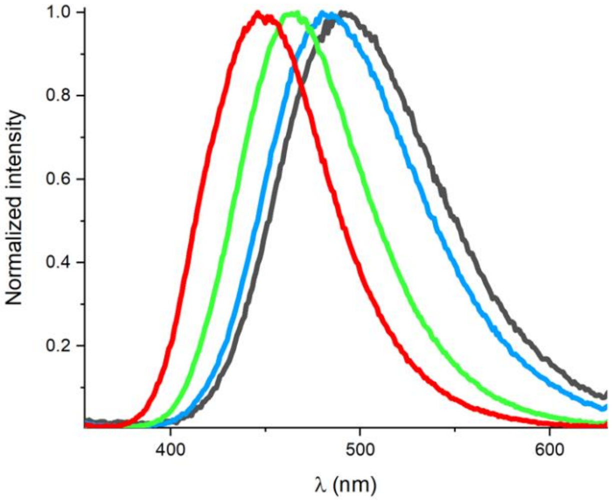

$\lambda_{excitation}$ = 336 nm

	Solvent	Peak (nm)
(black line)	MeCN	493
(blue line)	DMF	483
(green line)	DCM	464
(red line)	THF	448

Figure S24: Normalized fluorescence emission of ***5c*** *1·10^–5^ M in different solvents. MeCN (black trace); DMF (blue trace); DCM (green trace); THF (red trace).* $\lambda_{excitation}$ = 336 nm.

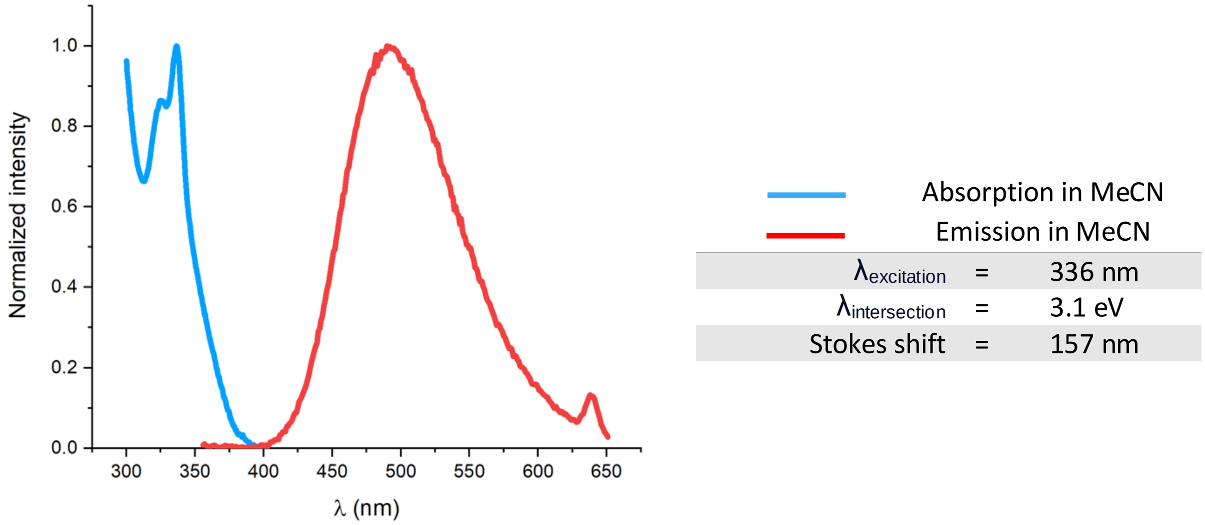

*Figure S25: Normalized optical absorption spectrum (blue trace) and emission spectra (red trace) of **5c**.* $\lambda_{excitation}$ = 336 nm.

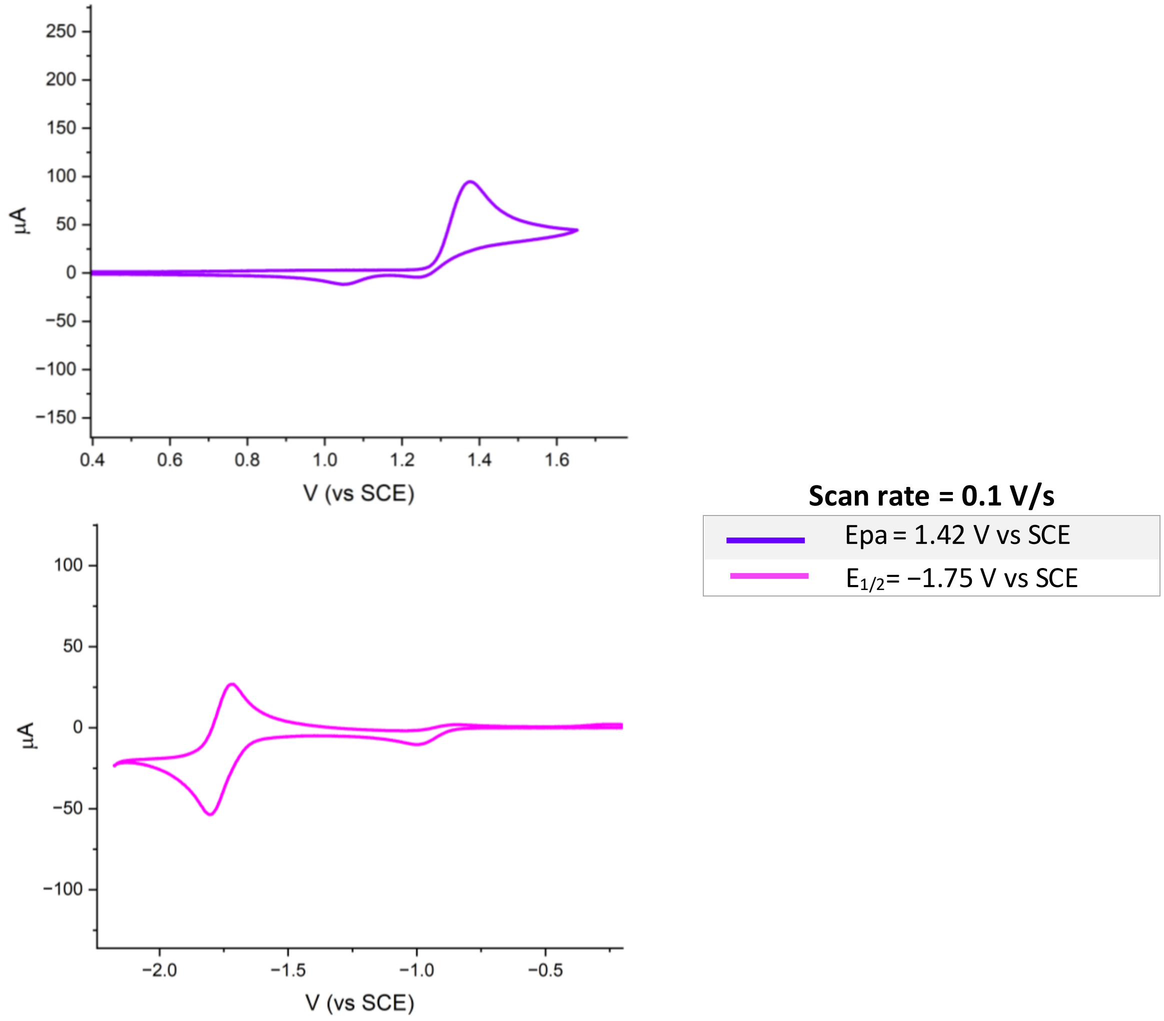

*Figure S26: Cyclic voltammograms of **5c** 10^{-3} M. Recorded with GC as the working electrode in MeCN with 0.1 M [Bu_4N][PF_6] as the supporting electrolyte at 0.1 V/s scan rate. Reversibility and irreversibility were determined by using different scan rates.*

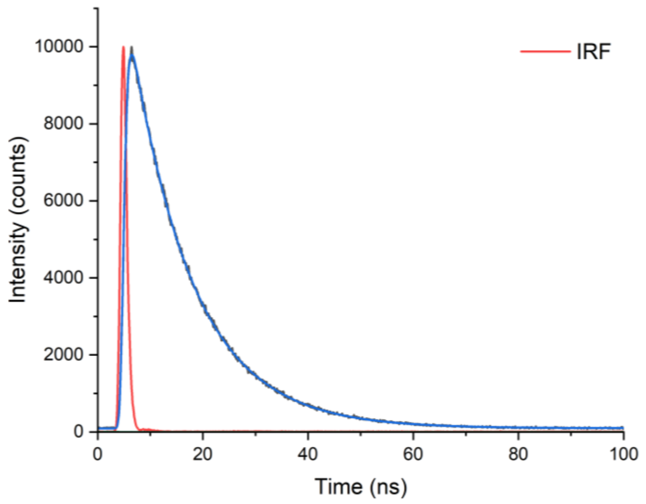

*Figure S27: Time-resolved emission decay (excitation at 340 nm) of **5c** $1{\cdot}10^{-5}$ M in MeCN solution measured by TC-SPC (τ = 11.6 ns), from deconvolution and single-exponential fitting, X2 = 1.19).*

2-(Diphenylamino)dibenzo[*b*,*d*]thiophene 5,5-dioxide (**5d**)

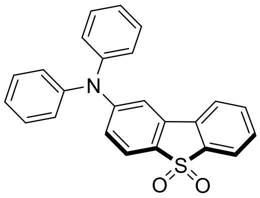

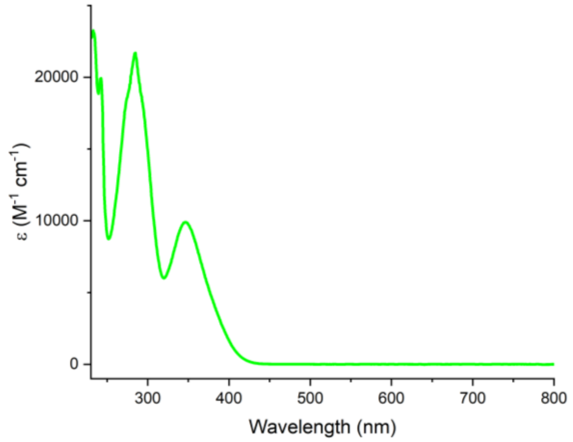

Concentration (mmol/mL)	
(green line)	*1·10⁻⁴*
λ peak	346 nm

*Figure S28: V UV–vis absorption of **5d** $1{\cdot}10^{-4}$ M (green trace). Recorded in MeCN in quartz cuvettes (1 cm optical path).*

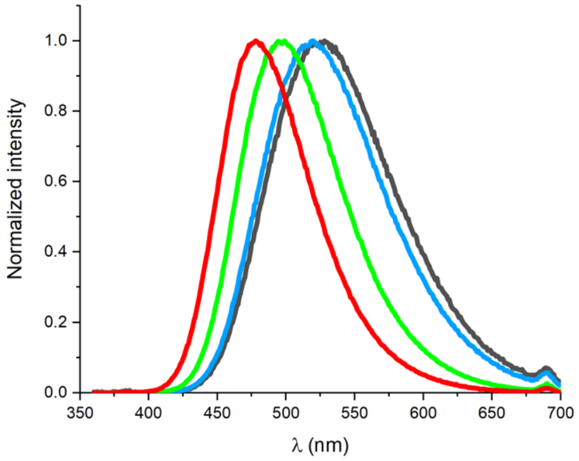

$\lambda_{excitation}$ = 345 nm

	Solvent	Peak (nm)
(black line)	MeCN	525
(blue line)	DMF	519
(green line)	DCM	497
(red line)	THF	477

*Figure S29: Normalized optical absorption spectrum (blue trace) and emission spectra (red trace) of **5d**.* $\lambda_{excitation}$ = 345 nm.

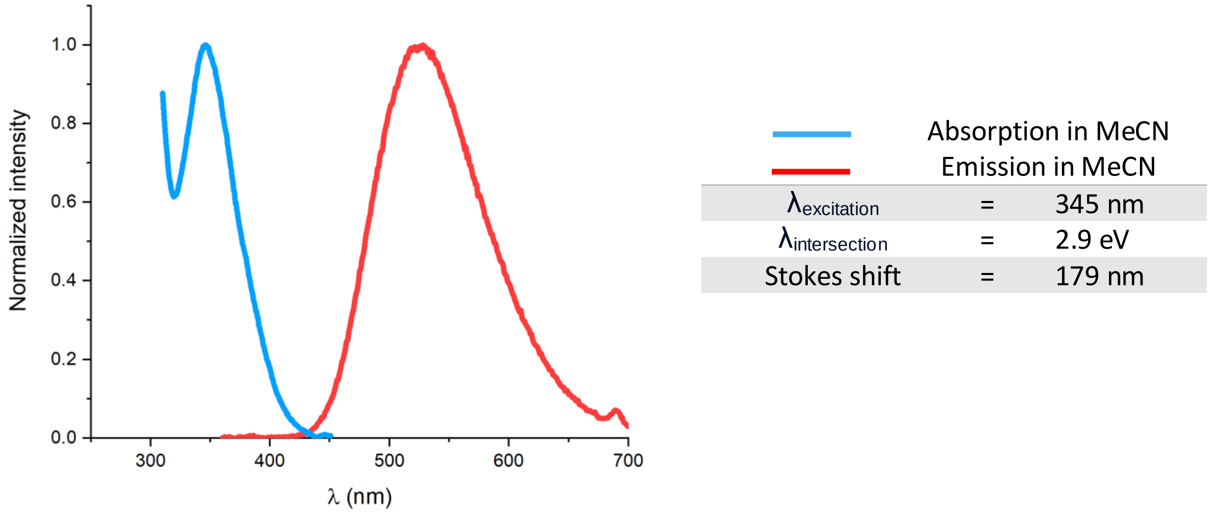

Figure S30: Normalized optical absorption spectrum (blue trace) and emission spectra (red trace) of ***5d****.* $\lambda_{excitation}$ = 345 nm

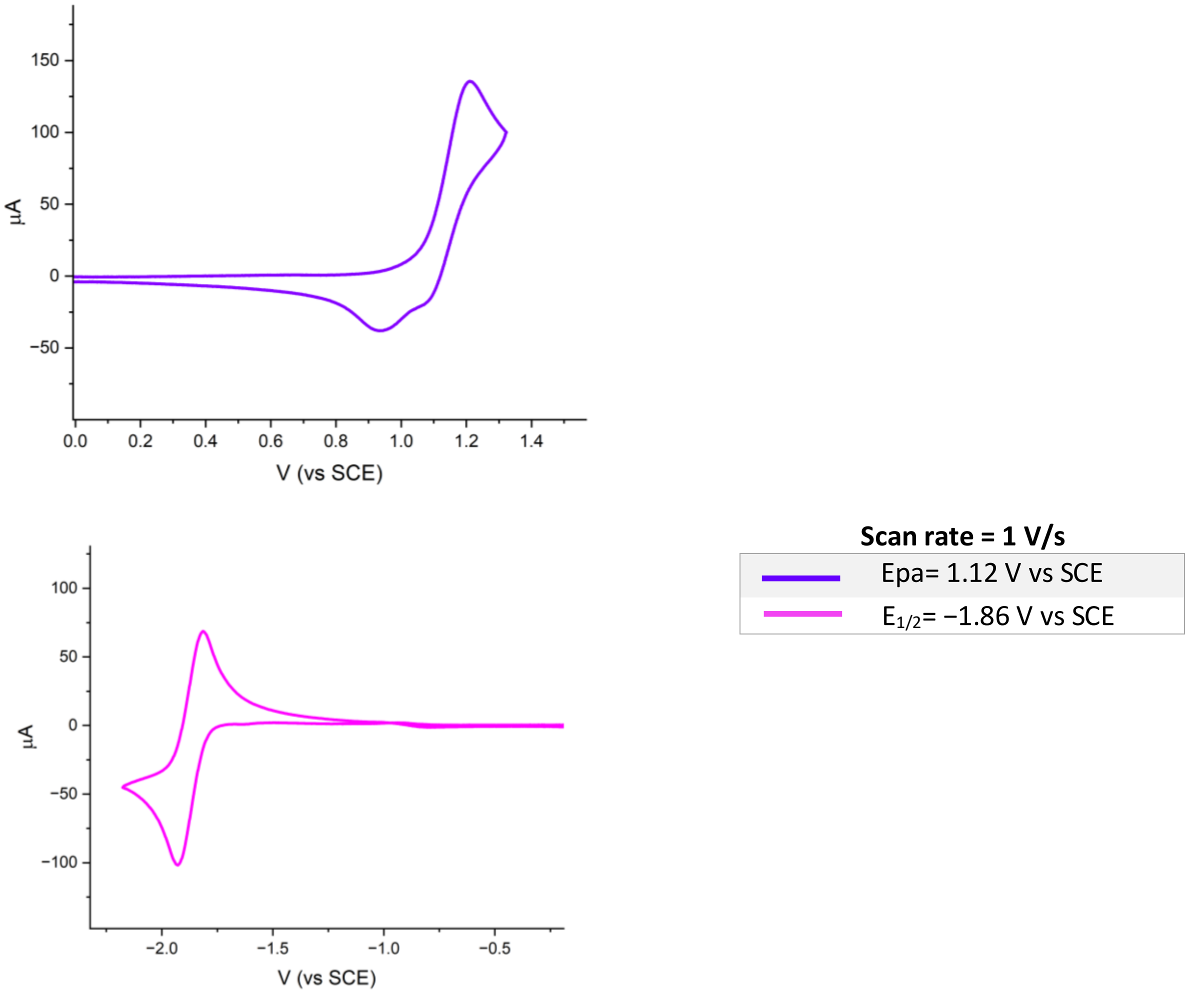

Figure S31. Cyclic voltammograms of ***5d*** *10^{-3} M. Recorded with GC as the working electrode in MeCN with 0.1 M $[Bu_4N][PF_6]$ as the supporting electrolyte at 1 V/s scan rate. Reversibility and irreversibility were determined by using different scan rates.*

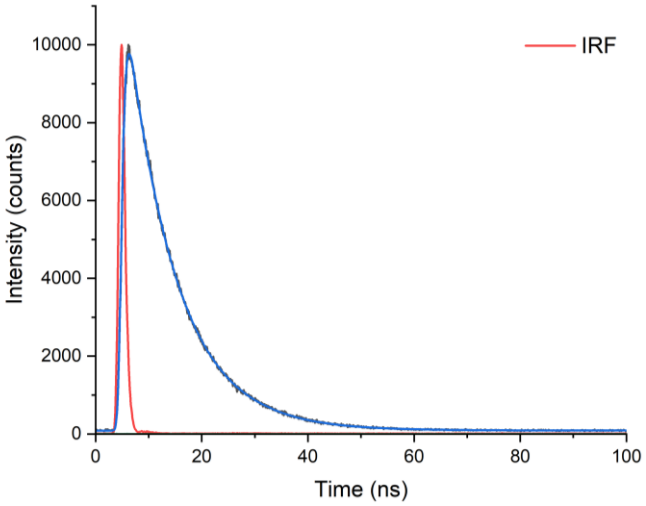

*Figure S32: Time-resolved emission decay (excitation at 340 nm) of **5d** $1{\cdot}10^{-5}$ M in MeCN solution measured by TC-SPC (τ = 9.1 ns), from deconvolution and single-exponential fitting, X2 = 1.19).*

2-(10*H*-Phenoxazin-10-yl)dibenzo[*b*,*d*]thiophene 5,5-dioxide (**5e**)

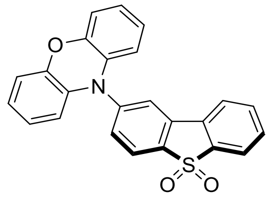

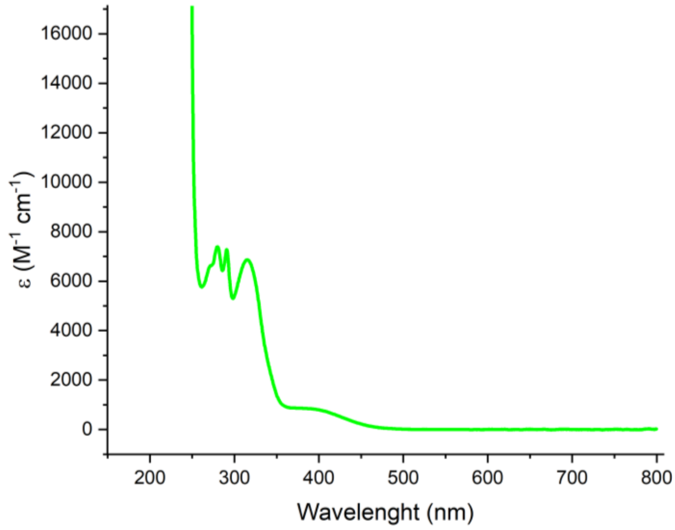

Concentration (mmol/mL)	
(green line)	*$1 \cdot 10^{-4}$*
λ peak	393 nm

*Figure S33: UV–vis absorption of **5e** at $1 \cdot 10^{-4}$ M. Recorded in MeCN in quartz cuvettes (1 cm optical path).*

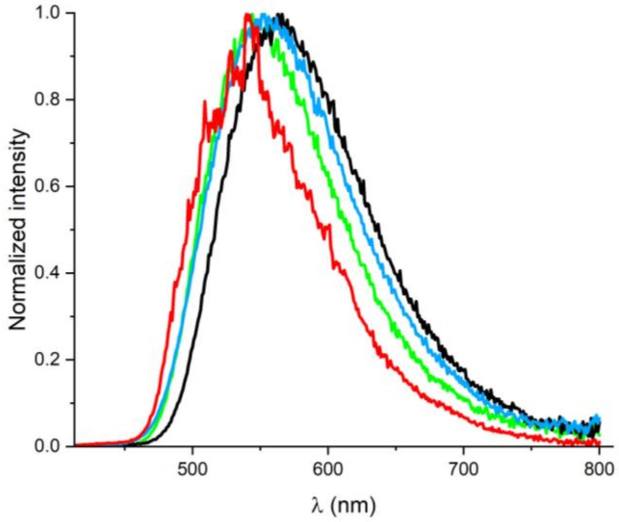

$\lambda_{excitation}$ = 400 nm

	Solvent	Peak (nm)
(black line)	MeCN	564
(blue line)	DMF	551
(green line)	DCM	542
(red line)	THF	537

*Figure S34: Normalized fluorescence emission of **5e** $1 \cdot 10^{-5}$ M in different solvents at 77 K. MeCN (black trace); DMF (blue trace); DCM (green trace); THF (red trace).* $\lambda_{excitation}$ = 400 nm.

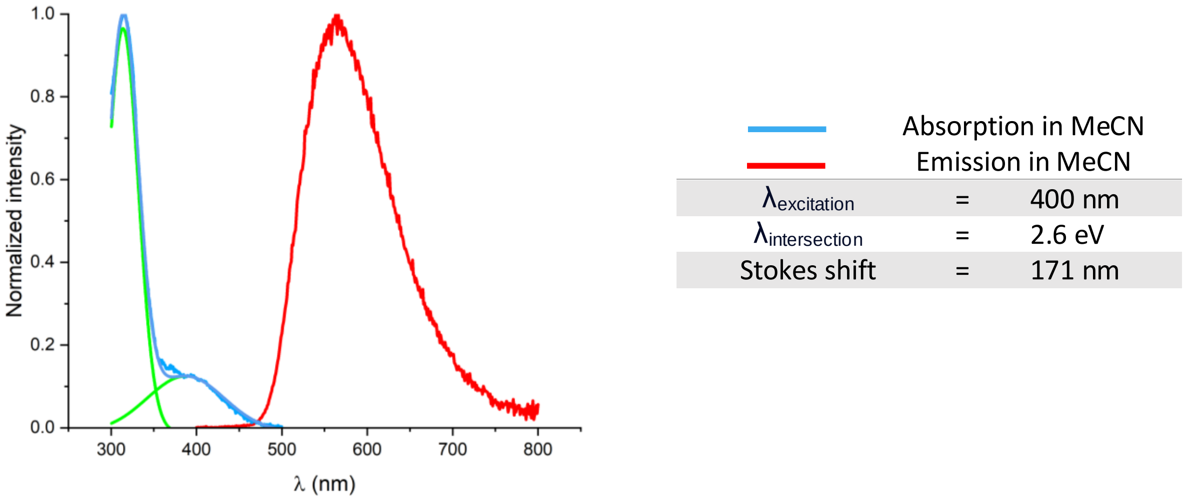

*Figure S35: Normalized optical absorption spectrum (blue trace) and emission spectra (red trace) of **5e**. Deconvoluted peaks in green.* $\lambda_{excitation}$ = 400 nm.

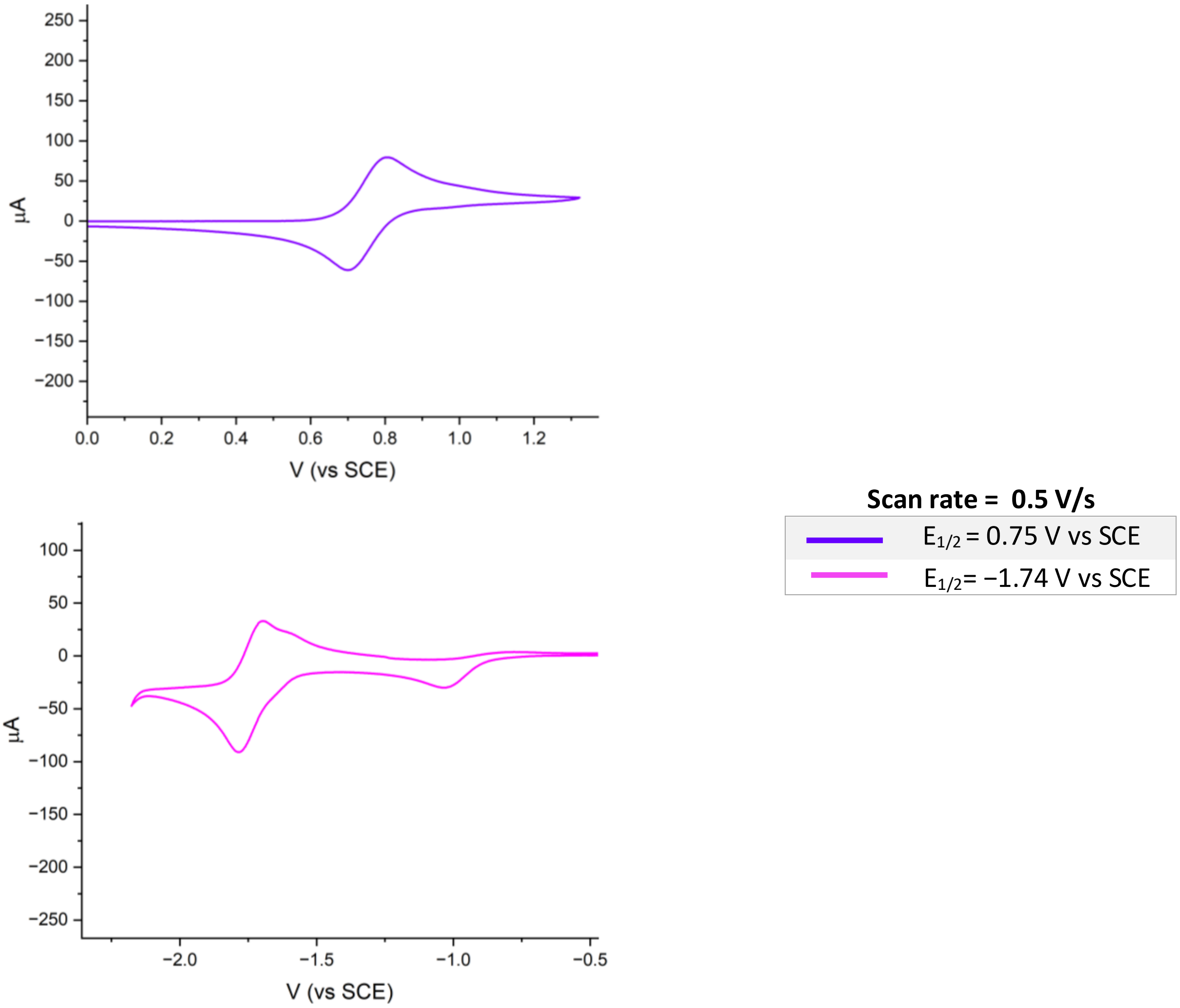

*Figure S36. Cyclic voltammograms of **5e** 10^{-3} M. Recorded with GC as the working electrode in MeCN with 0.1 M $[Bu_4N][PF_6]$ as the supporting electrolyte at 0.5 V/s scan rate. Reversibility was determined by using different scan rates.*

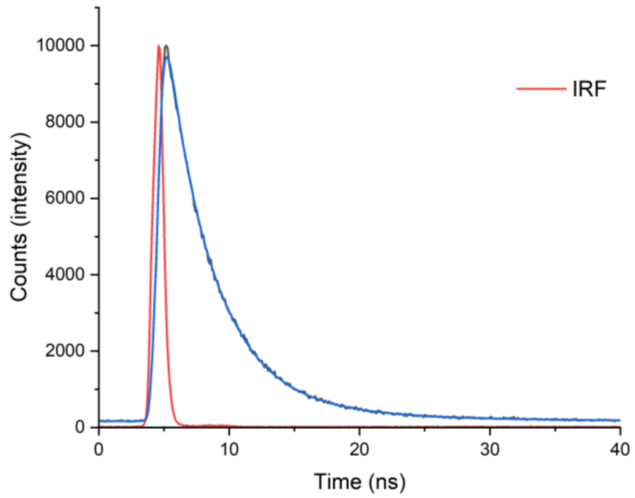

Figure S37.Time-resolved emission decay (excitation at 402.6 nm) of ***5e*** *1·10^{-5} M in MeCN solution measured by TC-(τAvInt= 4.45 ns), from deconvolution and single-exponential fitting, X2 = 1.16).*

F. Cyclic voltammetries of donors and acceptors

Donors analysis

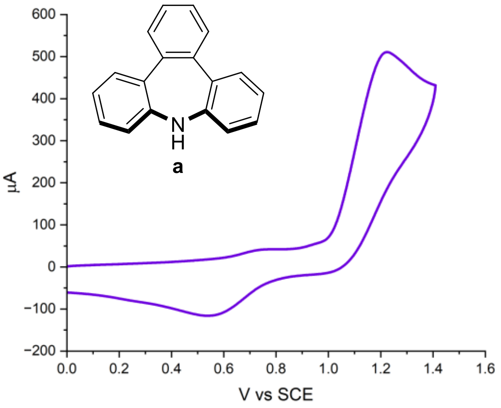

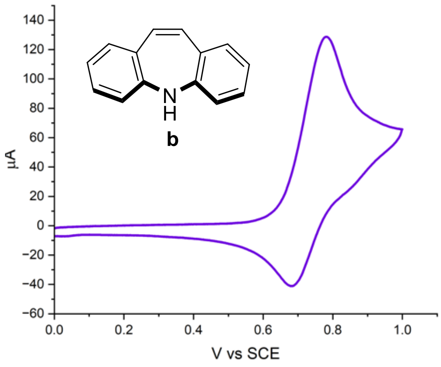

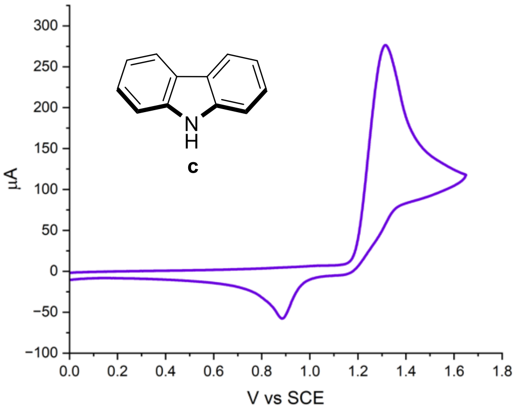

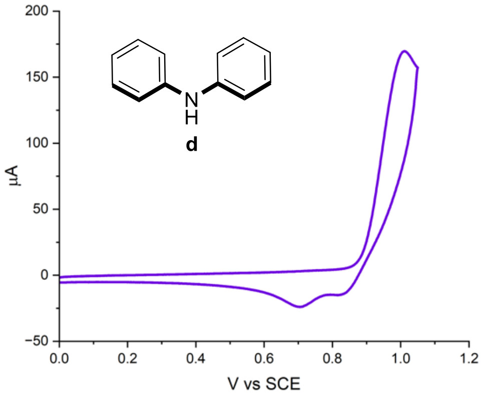

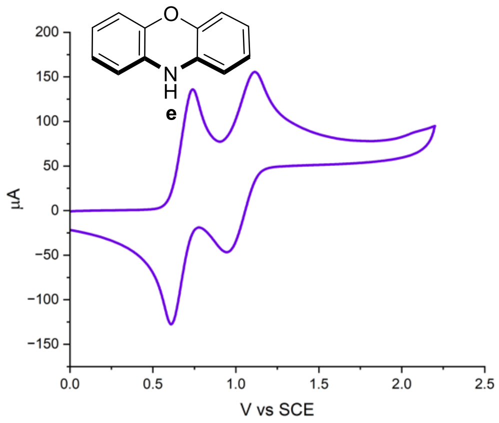

Acceptors analysis

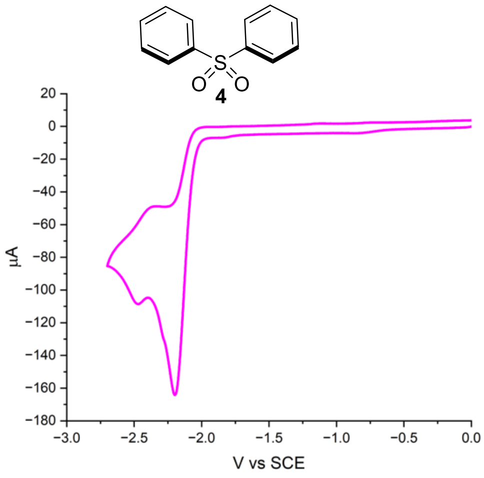

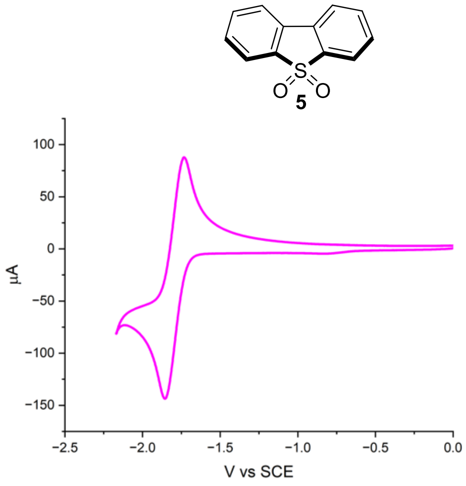

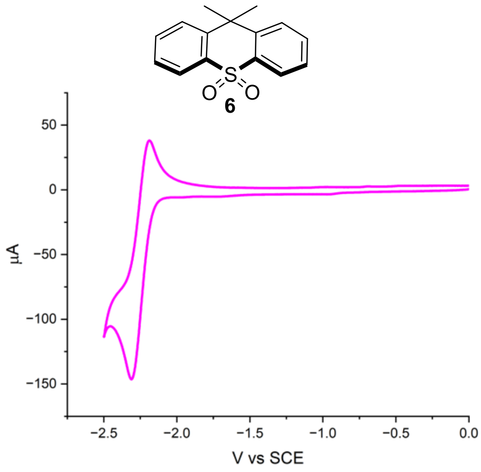

H. Evaluation of the photoredox performances

Oxidative quenching cycle study

General Procedure B

Dehalogenation of *p*-bromobenzonitrile

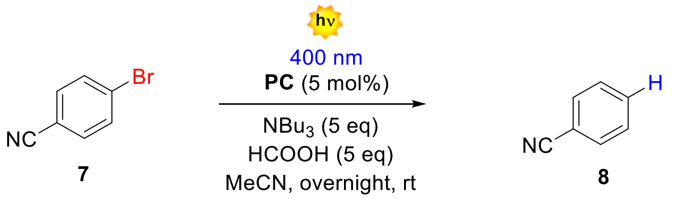

The procedure was followed according to the literature.[14] A 4 mL vial equipped with a septum was charged with a magnetic stirbar, 4-bromobenzonitrile **7** (18.2 mg, 0.1 mmol) and PC (5 mol %) and degassed with Ar. 1 mL of already degassed MeCN was added. Bu_3N (0.12 mL, 5 equiv) and HCOOH (19 µL, 5 equiv) were added via syringe and the reaction mixture was purged with Ar for 1 minute. The vial was irradiated for 12 hours with a 400 nm Kessil lamp set at 100% of its maximum output power.

PC	NMR Yield (%)
**4a**	N. R.
**5a**	63%
**6a**	N.R.
**5b**	58
**5c**	56
**5d**	44
**5e**	Traces

*Table S3: Results of the screening of different PCs in the reductive debromination of 4-bromobenzonitrile **7** according to General procedure B. CH_2Br_2 was used as an internal standard for the NMR yield calculation.*

General Procedure C

ATRA reaction of toluenesulfonyl chloride and styrene

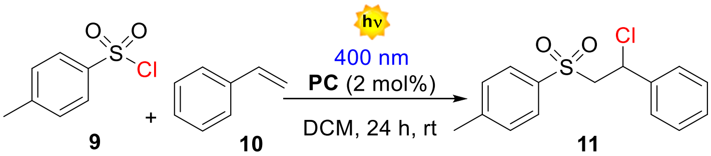

The procedure was followed according to the literature.[15] To an oven-dried vial was added *p*-toluenesulfonyl chloride (48 mg, 0.25 mmol, 1 equiv) styrene (0.017 mL, 0.25 mmol, 1 equiv.) and photocatalyst (2 mol %, 0.005 mmol). The vial was purged with a argon for 5 min and dry DCM (1.0 mL) was added before further argon purging for 10 min. The solution was stirred at room temperature while being irradiated a 400 nm Kessil lamp set at 100% of its maximum output power for 24 hours. After removal of solvent, the crude product was purified by flash column chromatography to afford the final product . **^{1}H NMR (400 MHz, $CDCl_3$)** δ 7.62 (d, *J* = 7.8 Hz, 2H), 7.28-7.21 (7H), 5.32 (t, *J* = 6.9 Hz, 1H), 3.93 (dd, *J* = 14.7, 6.9 Hz, 1H), 3.82 (dd, *J* = 14.8, 6.8 Hz, 1H), 2.40 (s, 3H). The ^{1}H NMR spectrum is consistent with that in the literature.[15]

PC	Yield (%)
**5a**	13%
**5b**	8%
**5c**	27 (20)
**5d**	12
**5e**	21

Table S4: Results of the screening of different PCs in the reductive ATRA reaction to obtain ***11*** *according to General procedure C. Isolated yield in parentheses.* CH_2Br_2 *was used as an internal standard for the NMR yield calculation.*

Reductive quenching cycle study

General Procedure D

Giese addition

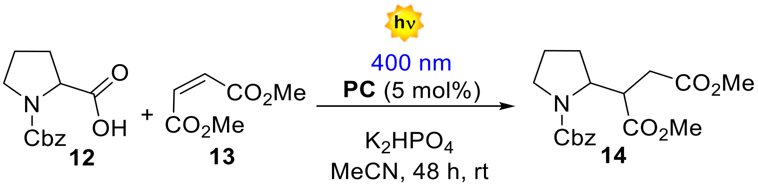

The procedure was followed according to the literature.[16] To an oven dried vial a magnetic stir bar was charged with the selected photocatalyst (0.005 mmol, 5 mol % cat. loading), Cbz-Pro-OH **12** (0.2 mmol, 1.0 equiv), methyl maleate **13** (0.2 mmol, 1.0 equiv), K_2HPO_4 (0.24 mmol, 1.2 equiv), and 0.5 mL of DMF. The reaction mixture was degassed using argon, then irradiated a 400 nm Kessil lamp set at 100% of its maximum output power for 48 h. Then, the reaction mixture was diluted with saturated aqueous $NaHCO_3$ solution and extracted with Et_2O (3 × 5 mL). The combined organic extracts were washed with water and brine, dried over $MgSO_4$ and concentrated in vacuo. Purification of the crude product by flash chromatography on silica gel afforded the desired product. **^{1}H NMR (400 MHz, $CDCl_3$)** δ 7.47-7.28 (m, 5H), 5.23 – 5.08 (m, 2H), 4.31 (dt, *J* = 8.1, 4.9 Hz, 1H), 4.20 – 4.09 (m, 1H), 3.73 – 3.44 (m, 2H), 3.46 – 3.21 (m, 1H), 2.78 (ddd, *J* = 29.0, 16.6, 10.1 Hz, 1H), 2.62 – 2.30 (m, 1H), 2.07 – 1.72 (m, 4H). The ^{1}H NMR spectrum is consistent with that in the literature.[17]

PC	Yield (%)
**5a**	76 (65)
**5b**	59
**5c**	65
**5d**	43
**5e**	5

Table S5: Results of the screening of different PCs in the Giese reaction to obtain ***14*** *according to General procedure D. Isolated yield in parentheses. CH_2Br_2 was used as an internal standard for the NMR yield calculation.*

General Procedure E

Pinacol coupling

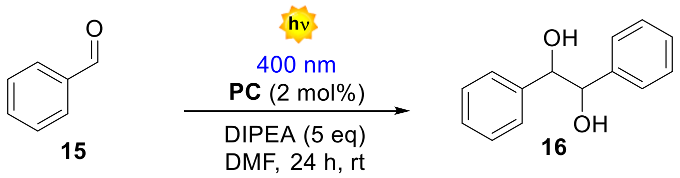

The procedure was followed according to the literature.[15] To an oven-dried vial was added benzaldehyde (0.020 mL, 0.2 mmol, 1 equiv), DIPEA (0.174 mL, 1 mmol, 5 equiv) and photocatalyst (2 mol %, 0.004 mmol). The vial was purged with argon for 5 min and dry DMF (2.0 mL) was added before further argon purging for 10 min. The reaction mixture was stirred at room temperature while being irradiated 400 nm Kessil lamp set at 100% of its maximum output power for 24 h. After removal of solvent, the crude product was purified by flash column to afford the product **18**. **^{1}H NMR (400 MHz, $CDCl_3$)** δ 7.33 (m, 7H), 7.31 – 7.21 (m, 11H), 7.15 (dd, J = 6.6, 3.0 Hz, 4H), 4.86 (s, 2H), 4.74 (s, 2H), 2.87 (s, 2H), 2.24 (s, 2H)**.** The ^{1}H NMR spectrum is consistent with that in the literature.[15]

PC	Yield (%)
**5a**	69 (60)
**5b**	41
**5c**	55
**5d**	51
**5e**	51

Table S6: Results of the screening of different PCs in the pinacol coupling to obtain ***16*** *according to General procedure E. Isolated yield in parentheses. CH_2Br_2 was used as an internal standard for the NMR yield calculation.*

I. Theoretical studies

DFT calculations

All the calculations were performed with the GAUSSIAN16 suite of programs (Revision C.01).[18] Each geometry was optimized with default convergence thresholds (RMS values of 3x10-4 hartree/bohr and 1.2 × 10^{-3} bohr and maximum values of 4.5x10-4 hartree/bohr and 1.8x10-3 bohr on forces and displacements, respectively) at WB97XD/Def2TZVP level of theory. Polarization effects were taken into account with the polarizable continuum model (PCM).

The energy gap between HOMO-LUMO was calculated using the ΔSCF method (Delta Self-Consistent Field).[19] This method estimates the HOMO and LUMO energies (and gap) by means of the energy of the potential of ionization (IP) and electron affinity (EA). These last values are calculated by the difference between the neutral form and the cation and anion species. All these energies are summarized in table S7.

IP = energy (cation) - energy (neutral)
EA = energy (neutral) – energy (anion)
HOMO-LUMO gap = IP-EA

The analysis of the excited state for each molecule was performed using TDDFT at WB97XD/Def2TZVP level of theory. Polarization effects were taken into account with the polarizable continuum model (PCM). Further analysis of the charge-transfer character via Δr,[20] NTOs and UV-vis simulated spectra was performed using multiwfn.[21,22] The major contributions and pair of orbitals involved for the first excited state, Δr, and excitation energies for the transition are summarized in table S8.

<u>Optimized geometries and SCF energies</u>

4a
SCF energy (neutral): -1759.30028 Hartree
SCF energy (cation): -1759.088569 Hartree
SCF energy (anion): -1759.35073 Hartree
Singlet, total number of atoms: 55
C 4.97154500 -0.01497700 0.18085200
C 5.36805800 -1.17939800 -0.47160800
C 1.62060300 1.15448300 0.61254300
C 2.24345300 -0.07322100 0.84052500
S 3.84823600 -0.12762200 1.56364400
O 4.02840300 1.06406100 2.37163900
O 4.03206400 -1.43226900 2.17170700
C 5.41406900 1.23609200 -0.23972500
C 6.26958900 1.31829600 -1.33195300
C 6.67156000 0.16111000 -1.99340600
C 6.22307500 -1.08488300 -1.56279200
C 0.37641300 1.20287600 0.01608900
C -0.27919000 0.01774900 -0.37859600
C 0.36251600 -1.21447100 -0.13536400
C 1.60704300 -1.25536500 0.46110100
N -1.51712500 0.06210300 -0.98115900
C -2.27400200 1.26937900 -1.10208700
C -3.26760600 1.54257000 -0.15192000

C -3.48539800 0.65291600 1.01314900
C -3.49339400 -0.75923100 0.92253000
C -3.28255300 -1.49499300 -0.34645700
C -2.28517900 -1.11291500 -1.25417700
C -2.03984400 2.12797200 -2.17118800
C -2.79391800 3.28485600 -2.31151900
C -3.79510900 3.56821200 -1.38572900
C -4.02885900 2.70708400 -0.32281700
C -3.71545000 1.24693400 2.26210400
C -3.94599000 0.49039100 3.39999400
C -3.95518500 -0.89782300 3.31069900
C -3.73271800 -1.50535500 2.08525200
C -4.05497200 -2.62060100 -0.66430400
C -3.82871300 -3.34178400 -1.82813000
C -2.82370000 -2.95287900 -2.71039700
C -2.05847000 -1.83083000 -2.42397000
H 5.01940300 -2.14307600 -0.12605400
H 2.10574100 2.07341800 0.91336600
H 5.10043100 2.12857500 0.28430700
H 6.62336400 2.28602400 -1.66412800
H 7.33813400 0.22977800 -2.84398200
H 6.54063400 -1.98442000 -2.07464200
H -0.09798800 2.16062700 -0.14084400
H -0.12321300 -2.13944000 -0.40984900
H 2.08167500 -2.20993500 0.64431900
H -1.26116600 1.87982300 -2.88104400
H -2.60943700 3.95483900 -3.14164600
H -4.40040000 4.45928300 -1.49612700
H -4.81976600 2.93149400 0.38120000
H -3.69264700 2.32616500 2.34002800
H -4.10577700 0.98134100 4.35184600
H -4.12271800 -1.50434100 4.19196000
H -3.72373600 -2.58596400 2.02462000
H -4.84927500 -2.92444500 0.00514400
H -4.44289700 -4.20524500 -2.05133100
H -2.64497300 -3.51314300 -3.61937700
H -1.27704600 -1.50162500 -3.09692200

5a
SCF energy (neutral): -1758.097921 Hartree
SCF energy (cation): -1757.885708 Hartree
SCF energy (anion): -1758.167476 Hartree
Singlet, total number of atoms: 53
C 4.42932700 0.07155200 0.08961500
C 3.28391000 0.81821400 -0.19679400
C 2.08325500 -0.01986200 -0.37607200
C 2.34553500 -1.38444700 -0.21757900
S 4.04774600 -1.66748600 0.14366900
O 4.71619400 -2.37783400 -0.93255000
O 4.23349400 -2.19750100 1.48308000
C 5.66992800 0.64328800 0.29709400
C 5.76481900 2.03144100 0.21424200
C 4.63559000 2.79819600 -0.06940300
C 3.39348400 2.20232300 -0.27635200
C 0.79952600 0.40464100 -0.66397000

C -0.23802300 -0.54113200 -0.79672900
C 0.05521400 -1.91181900 -0.62457900
C 1.34286800 -2.33229800 -0.33691000
N -1.52172100 -0.12981200 -1.09213600
C -1.89956300 1.25012800 -1.08539800
C -2.43186600 1.80094100 0.08871000
C -2.53046800 1.00671300 1.33600500
C -2.97151100 -0.33765600 1.36900900
C -3.38124500 -1.09064000 0.15999500
C -2.64284000 -1.01648100 -1.02938700
C -1.76288600 2.00841900 -2.24312100
C -2.15476100 3.34021400 -2.25190100
C -2.69894600 3.90300500 -1.10012200
C -2.83667400 3.14222600 0.05250600
C -2.18462300 1.62979200 2.54355200
C -2.25984800 0.96468600 3.75693500
C -2.69324300 -0.35676000 3.78935400
C -3.04384300 -0.99022800 2.60777100
C -4.50351100 -1.92996000 0.17784500
C -4.86157300 -2.67363800 -0.93800700
C -4.10696300 -2.59495800 -2.10583300
C -3.00070500 -1.75738600 -2.15048900
H 6.53878600 0.03616600 0.51689600
H 6.72065600 2.51410300 0.37152900
H 4.72453900 3.87549400 -0.13016900
H 2.52480700 2.80932300 -0.49552800
H 0.59193600 1.45800200 -0.78184500
H -0.73332300 -2.64443100 -0.71376800
H 1.55111800 -3.38668000 -0.20605500
H -1.34376500 1.54374900 -3.12645600
H -2.04620100 3.93136900 -3.15232600
H -3.02403100 4.93589200 -1.10188900
H -3.27531000 3.58766600 0.93603100
H -1.83022100 2.65220500 2.52261400
H -1.97089400 1.47126800 4.66932500
H -2.74662200 -0.89522900 4.72728900
H -3.36414800 -2.02359800 2.63655100
H -5.10746600 -1.98872200 1.07413800
H -5.73617200 -3.31095600 -0.89884100
H -4.38400000 -3.17403000 -2.97760300
H -2.40127000 -1.67155500 -3.04778100

5b
SCF energy (neutral): -1604.441612 Hartree
SCF energy (cation): -1604.229944 Hartree
SCF energy (anion): -1604.506716 Hartree
Singlet, total number of atoms: 47
C -0.63238400 -0.46483600 -0.34459400
C -0.35129000 -1.84667700 -0.25558300
C 0.94906900 -2.30286100 -0.11678500
C 1.98059500 -1.38087000 -0.06327500
C 1.73304100 -0.00688500 -0.13809400
C 0.43675600 0.45318800 -0.27596900
S 3.70454100 -1.70856800 0.10925200
C 4.11547700 0.02476700 0.08934400

C 2.96308900 0.80221800 -0.04859100
C 5.38239700 0.56637300 0.19265000
C 5.49772200 1.95494800 0.15604400
C 4.36219100 2.75191100 0.01855800
C 3.09324700 2.18633700 -0.08470800
N -1.93244000 -0.01638100 -0.49763400
C -2.24722700 1.37552300 -0.41330100
C -2.65766500 1.91435700 0.81820800
C -2.91735600 1.08879300 1.99226400
C -3.36134000 -0.18355400 2.01622600
C -3.68659200 -1.02975800 0.87329900
C -3.04418400 -0.90608600 -0.37071800
C -4.70450400 -1.99011900 0.99201400
C -5.09367500 -2.76114000 -0.09185100
C -4.47142900 -2.59455000 -1.32839200
C -3.44707800 -1.66725400 -1.46337900
C -2.09664500 2.18534200 -1.53456500
C -2.31451400 3.55331500 -1.44181900
C -2.68306600 4.11258900 -0.21890200
C -2.85032500 3.30343700 0.89373800
O 4.01408300 -2.30043200 1.39893500
O 4.24453100 -2.38166800 -1.05905600
H -1.15723300 -2.56387500 -0.29248700
H 1.14404400 -3.36567500 -0.04917200
H 0.24759800 1.51476500 -0.32602100
H 6.25603900 -0.06384000 0.29864600
H 6.47456100 2.41408800 0.23500700
H 4.46741000 3.82909400 -0.00872200
H 2.22029100 2.81686900 -0.19198000
H -2.83962200 1.60403400 2.94394900
H -3.60972900 -0.60284900 2.98565600
H -5.19797800 -2.11359200 1.94869800
H -5.88504900 -3.49122900 0.02338500
H -4.77381900 -3.19358400 -2.17795800
H -2.93065800 -1.54221300 -2.40658000
H -1.78044400 1.73427600 -2.46654400
H -2.18476100 4.18184600 -2.31367100
H -2.84307600 5.18040800 -0.13694700
H -3.14793300 3.73896800 1.84018500

5c
SCF energy (neutral): -1527.051144 Hartree
SCF energy (cation): -1526.83989 Hartree
SCF energy (anion): -1527.128023 Hartree
Singlet, total number of atoms: 43
C 5.26186600 1.94775200 -1.41640500
C 4.05517600 2.54970100 -1.77016700
C 2.83522100 2.00272200 -1.37960200
C 2.82802300 0.83583600 -0.62368000
C 4.04972700 0.25158400 -0.28078300
C 5.26999000 0.77842400 -0.65828100
C 1.66739000 0.09107000 -0.10443800
C 2.02567000 -1.04617000 0.62286600
S 3.79296500 -1.21948800 0.68627200
C 0.32219500 0.38611300 -0.27025000

C -0.63394500 -0.45070700 0.30932100
C -0.25183900 -1.57945900 1.04035800
C 1.09384400 -1.89070800 1.19509600
O 4.22764200 -2.40118100 -0.03270300
O 4.28688600 -1.04717900 2.03855800
N -2.00614600 -0.14958700 0.15412500
C -2.61137200 1.07158600 0.45590600
C -3.99187300 0.98240600 0.16336000
C -4.22847000 -0.34949100 -0.34156200
C -2.98362600 -1.02012200 -0.33118400
C -2.05578900 2.22912900 0.99684000
C -2.90312600 3.30610600 1.22473400
C -4.27173000 3.23777100 0.92720300
C -4.82266300 2.07801500 0.40052300
C -5.36697100 -1.00861000 -0.80647600
C -5.24988200 -2.31604100 -1.25676600
C -4.00581600 -2.96296500 -1.25304800
C -2.85840800 -2.32805700 -0.79468200
H 6.19869900 2.38889400 -1.73075700
H 4.06530300 3.45792800 -2.35919300
H 1.90506100 2.47914700 -1.66057000
H 6.19969300 0.30095100 -0.37672000
H 0.01043800 1.24664800 -0.84558500
H -1.00901700 -2.20422600 1.49288300
H 1.39502800 -2.76279500 1.76065700
H -1.00343600 2.29213100 1.23667000
H -2.49410500 4.21733400 1.64338900
H -4.90242400 4.09729400 1.11625400
H -5.88129300 2.01815000 0.17945300
H -6.32424200 -0.50205600 -0.81778900
H -6.12396900 -2.84227700 -1.61921200
H -3.93387800 -3.98067400 -1.61640300
H -1.90405900 -2.83594600 -0.80245000

5d
SCF energy (neutral): -1528.233265 Hartree
SCF energy (cation): -1528.033547 Hartree
SCF energy (anion): -1528.30514 Hartree
Singlet, total number of atoms: 45
C -5.29661900 1.92331000 -0.87118200
C -4.13601200 2.66311400 -1.09299300
C -2.87689700 2.11728500 -0.85370500
C -2.78254700 0.81086100 -0.38669800
C -3.95943600 0.08960200 -0.17170400
C -5.21723700 0.61331300 -0.40194900
C -1.56885700 0.03796200 -0.06484100
C -1.85020200 -1.25522000 0.38599900
S -3.59235400 -1.54655800 0.42814100
C -0.25360800 0.45149000 -0.17555300
C 0.78832700 -0.42494700 0.17767900
C 0.47314400 -1.72017000 0.63273100
C -0.84387800 -2.14000300 0.73308300
O -4.07376000 -1.72051600 1.78666800
O -3.98725900 -2.55582800 -0.53779900
N 2.11869100 -0.01270500 0.07939400

C 2.47151900 1.36017100 0.21726600
C 3.32494700 1.95809400 -0.71156900
C 4.31587900 -0.93258300 0.61629100
C 3.16062800 -0.95206000 -0.16688600
C 1.98678500 2.11217900 1.28963700
C 2.34224800 3.44851800 1.42033400
C 3.19670200 4.04444400 0.49609200
C 3.68881900 3.29145700 -0.56624600
C 5.34399900 -1.83281800 0.36375800
C 5.22619600 -2.76711600 -0.66147500
C 4.07209900 -2.78794800 -1.44056500
C 3.04692600 -1.88128100 -1.20293200
H -6.26524600 2.36632800 -1.06294600
H -4.21353600 3.67975100 -1.45706200
H -1.98372000 2.70267400 -1.02921600
H -6.11088600 0.02889600 -0.22426700
H -0.02722100 1.44351700 -0.53832700
H 1.26746700 -2.39513700 0.91640700
H -1.07084100 -3.13614000 1.09111000
H 1.33259400 1.64722500 2.01561700
H 1.96070700 4.02169900 2.25639500
H 3.47759500 5.08445500 0.60437200
H 4.35150300 3.74487400 -1.29311600
H 6.23562500 -1.80948100 0.97820500
H 6.02646600 -3.47075300 -0.85308200
H 3.97348000 -3.50404800 -2.24716200
H 2.15582300 -1.89018000 -1.81702900
H 3.70232200 1.37626700 -1.54249300
H 4.40527900 -0.21125700 1.41818400

5e
SCF energy (neutral): -1602.252821 Hartree
SCF energy (cation): -1602.068807 Hartree
SCF energy (anion): -1602.330657 Hartree
Singlet, total number of atoms: 44
C 5.21709100 0.00044700 2.52906400
C 3.99008200 0.00050300 3.19108600
C 2.78993900 0.00034400 2.48435700
C 2.82393700 0.00012200 1.09435700
C 4.06516800 0.00004900 0.45331400
C 5.26633000 0.00021400 1.13639200
C 1.68934200 -0.00001800 0.15517500
C 2.08287300 -0.00019800 -1.18499800
S 3.85895200 -0.00025900 -1.31369900
C 0.33276600 0.00003300 0.45087600
C -0.58586500 -0.00009600 -0.59587900
C -0.17193500 -0.00026400 -1.92614700
C 1.18550700 -0.00032000 -2.23490700
O 4.33658300 1.23790300 -1.89637500
O 4.33646500 -1.23870000 -1.89590100
N -1.98343500 -0.00004000 -0.29956300
C -2.65600600 -1.21070100 -0.08778600
C -4.01980600 -1.18503400 0.24028500
O -4.71672300 0.00011200 0.34507100
C -4.01972500 1.18519200 0.24006600

C -2.65592500 1.21070900 -0.08801200
C -2.02906300 -2.45214700 -0.19314900
C -2.74102600 -3.63135200 0.02237600
C -4.08800800 -3.58847200 0.34931700
C -4.72574500 -2.35258300 0.45889300
C -4.72558800 2.35282800 0.45845300
C -4.08777500 3.58865400 0.34863200
C -2.74079200 3.63138500 0.02167100
C -2.02890600 2.45209400 -0.19363000
H 6.13791100 0.00058300 3.09748200
H 3.96874200 0.00067900 4.27345000
H 1.84343700 0.00039100 3.00902800
H 6.21236600 0.00016500 0.61069300
H -0.01736800 0.00016200 1.47441700
H -0.91491800 -0.00036400 -2.71240900
H 1.51981600 -0.00044900 -3.26410900
H -0.97946600 -2.49844900 -0.44705800
H -2.22866800 -4.58062500 -0.06859700
H -4.64649900 -4.49963300 0.51912800
H -5.77630100 -2.28113700 0.71105900
H -5.77614900 2.28150000 0.71063300
H -4.64620800 4.49988100 0.51827100
H -2.22837200 4.58060600 -0.06949400
H -0.97931100 2.49827700 -0.44756900

6a
SCF energy (neutral): -1876.045154 Hartree
SCF energy (cation): -1875.832528 Hartree
SCF energy (anion): -1876.095826 Hartree
Singlet, total number of atoms: 62
C 5.98717600 0.52600400 1.80866200
C 5.53384900 1.80422300 1.50824100
C 4.46221900 1.99249700 0.64232500
C 3.80497400 0.91842200 0.03492100
C 4.28846200 -0.35757400 0.35990300
C 5.35256900 -0.56346600 1.23330000
C 2.65621000 1.13440000 -0.96764300
C 1.57800400 0.04347700 -0.82493900
C 1.90170000 -1.29839800 -0.56246900
S 3.56717300 -1.80571200 -0.36100500
C 0.23220800 0.34476200 -0.98515000
C -0.77797900 -0.62978700 -0.89973600
C -0.41508500 -1.96036000 -0.63136800
C 0.91526700 -2.27822300 -0.46022900
N -2.10406900 -0.27451000 -1.07043700
C -2.51335700 1.09631500 -1.12327600
C -2.82459100 1.75928900 0.07230800
C -2.65945400 1.09312200 1.38561100
C -3.05123500 -0.24400000 1.63392000
C -3.66493300 -1.11772000 0.60603300
C -3.17041000 -1.15724900 -0.70566700
C -2.61821300 1.73643300 -2.35311900
C -3.03769400 3.05855500 -2.41531000
C -3.36459300 3.73077000 -1.24038200
C -3.26036500 3.08827100 -0.01441900

C -2.09844100 1.83462300 2.43499100
C -1.91816400 1.29303800 3.69771200
C -2.30513300 -0.02058400 3.94240000
C -2.86447300 -0.77027700 2.91991800
C -4.73830500 -1.96095700 0.92278500
C -5.28350500 -2.81591400 -0.02517200
C -4.76987600 -2.84842500 -1.31906900
C -3.71571200 -2.01058800 -1.65809500
O 4.18977200 -2.04990600 -1.65441400
O 3.62775900 -2.89838400 0.59399400
C 2.04839700 2.53760300 -0.81275700
C 3.24311600 1.05359100 -2.40591900
H 6.81611500 0.37702100 2.48828700
H 6.00937800 2.66797900 1.95601300
H 4.14021600 3.00365700 0.44825800
H 5.66954300 -1.57188600 1.46404400
H -0.06914500 1.36177500 -1.16679000
H -1.16841900 -2.72942100 -0.54251700
H 1.19617200 -3.29791900 -0.23176500
H -2.36274700 1.18832700 -3.25103100
H -3.11726400 3.55787800 -3.37258500
H -3.70833900 4.75687200 -1.27960600
H -3.53088900 3.61790200 0.88992200
H -1.77925100 2.85118800 2.24514900
H -1.46725600 1.88850600 4.48170000
H -2.16064800 -0.46334400 4.91998100
H -3.14598300 -1.79771600 3.11094900
H -5.15562300 -1.93541800 1.92117100
H -6.11607300 -3.45361700 0.24467300
H -5.19264700 -3.51508000 -2.05996200
H -3.30158300 -2.01066000 -2.65826800
H 1.60526900 2.68613300 0.17303200
H 2.80690700 3.30089100 -0.97392400
H 1.28392400 2.70756200 -1.56847600
H 3.99922200 1.83012600 -2.53524800
H 3.70061100 0.08787200 -2.60730900
H 2.44617400 1.21942200 -3.13308500

Additional data: HOMO and LUMO energies and MOs primarily involved in the formation of the lowest excited states

PC	IP (eV)	EA (eV)	Gap (HOMO-LUMO) (eV)
**4a**	5.8	1.4	4.4
**5a**	5.8	1.9	3.9
**5b**	5.8	1.8	4.0
**5c**	5.8	2.1	3.7
**5d**	5.4	2.0	3.5
**5e**	5.0	2.1	2.9
**6a**	5.8	1.4	4.4

Table S7. Energy gaps.

The most relevant transition involved in the formation of the lowest excited state is provided for each compound; the corresponding coefficients in the CI expansions are also reported; calculations were performed with Time-Dependent DFT, with the employment of the SMD.

PC	Transition Energy (eV)	Major MO pair involved	First Eigenvalue (Sum of eigenvalues)	Contribution to the transition	Δr (Å)
**4a**	4.5	HOMO -> LUMO	0.932714 (0.987747)	94.4%	3.318262
**5a**	4.0	HOMO -> LUMO	0.969287 (0.991630)	97.7%	2.621680
**5b**	4.0	HOMO -> LUMO	0.894748 (0.992469)	90.1%	2.403746
**5c**	4.2	HOMO -> LUMO	0.911335 (0.990330)	92.0%	3.573332
**5d**	3.8	HOMO -> LUMO	0.963456 (0.992229)	97.1%	3.526836
**5e**	3.7	HOMO -> LUMO	0.991643 (0.996231)	99.5%	4.977297
**6a**	4.6	HOMO -> LUMO	0.909678 (0.989574)	91.9%	2.829092

Table S8. Analysis for the first excited state.

J. References

1. Ward, J. S.; Nobuyasu, R. S.; Batsanov, A. S.; Data, P.; Monkman, A. P.; Dias, F. B.; Bryce, M. R. *Chem. Commun.,* **2016**, *52*, 2612–2615.
2. Hu, M.; Liu, D.; Liu, Y.; Luo, F.; Chen, X.; Yin, Y.; Zhang, S.; Hu, Y. *Adv. Synth. Catal.,* **2024**, *366*, 1538–1544.
3. Braveenth, R.; Bae, I. J.; Raagulan, K.; Kim, S.; Kim, S.; Kim, M.; Chai, K. Y.. *Dyes and Pigments,* **2019**, *162*, 466–474.
4. Jang, H. J.; Braveenth, R.; Raagulan, K.; Choi, S. Y.; Park, Y. H.; Oh, S. Bin; Bae, I. J.; Kim, B. M.; Wu, Q.; Kim, M.; Chai, K. Y. *Dyes and Pigments,* **2020**, *182*, 108697.
5. Sun, N.; Zheng, K.; Zhang, M.; Zheng, G.; Jin, L.; Hu, B.; Shen, Z.; Hu, X. *Green Chemistry,* **2023**, *25*, 2782–2789.
6. Zhang, Y.; Yang, Z.; Yang, H.; Li, X.; Yang, L. *Org. Biomol. Chem.,* **2024**, *22*, 3381–3385.
7. Emslie, D. J. H.; Blackwell, J. M.; Britten, J. F.; Harrington, L. E. *Organometallics.,* **2006**, *25*, 2412–2414.
8. Fu, C.; Luo, S.; Li, Z.; Ai, X.; Pang, Z.; Li, C.; Chen, K.; Zhou, L.; Li, F.; Huang, Y.; Lu, Z. *Chem. Commun.,* **2019**, *55*, 6317–6320
9. Fang, M. Y.; Chen, L. P.; Huang, L.; Fang, D. M.; Chen, X. Z.; Wang, B. Q.; Feng, C.; Xiang, S. K. *J. Org. Chem.,* **2021**, *86*, 9096–9106.
10. Gan, S.; Luo, W.; He, B.; Chen, L.; Nie, H.; Hu, R.; Qin, A.; Zhao, Z.; Tang, B. Z. *J. Mater. Chem. C. Mater.,* **2016**, *4*, 3705–3708.
11. Higginbotham, H. F.; Yi, C. L.; Monkman, A. P.; Wong, K. T. *J. Phys. Chem. C.,* **2018**, *122*, 7627–7634.
12. Würth, C.; Grabolle, M.; Pauli, J.; Spieles, M.; Resch-Genger, U. Nat. Prot., **2013** , 8, 1535–1550.
13. Romero, N. A.; Nicewicz, D. A. Chem. Rev., **2016**, 116, 10075– 10166.
14. Bortolato, T.; Simionato, G.; Vayer, M.; Rosso, C.; Paoloni, L.; Benetti, E. M.; Sartorel, A.; Lebœuf, D.; Dell'Amico, L. *J. Am. Chem. Soc.,* **2023**, *145*, 1835–1846.
15. Bryden, M. A.; Millward, F.; Matulaitis, T.; Chen, D.; Villa, M.; Fermi, A.; Cetin, S.; Ceroni, P.; Zysman-Colman, E. *J. Org. Chem.,* **2023**, *88*, 6364–6373.
16. Mateos, J.; Rigodanza, F.; Vega-Peñaloza, A.; Sartorel, A.; Natali, M.; Bortolato, T.; Pelosi, G.; Companyó, X.; Bonchio, M.; Dell'Amico, L. *Angew. Chem. Int. Ed.,* **2020**, *59*, 1302–1312.
17. Vijayakrishnan, S.; Ward, J. W.; Cooper, A. I. *ACS Catal.,* **2022**, *12*, 10057–10064.
18. Frisch, M. J.; Trucks, G. W.; Schlegel, H. B.; Scuseria, G. E.; Robb, M. A.; Cheeseman, J. R.; Scalmani, G.; Barone, V.; Petersson, G. A.; Nakatsuji, H.; Li, X.; Caricato, M.; Marenich, A. V.; Bloino, J.; Janesko, B. G.; Gomperts, R.; Mennucci, B.; Hratchian, H. P.; Ortiz, J. V.; Izmaylov, A. F.; Sonnenberg, J. L.; Williams-Young, D.; Ding, F.; Lipparini, F.; Egidi, F.; Goings, J.; Peng, B.; Petrone, A.; Henderson, T.; Ranasinghe, D.; Zakrzewski, V. G.; Gao, J.; Rega, N.; Zheng, G.; Liang, W.; Hada, M.; Ehara, M.; Toyota, K.; Fukuda, R.; Hasegawa, J.; Ishida, M.; Nakajima, T.; Honda, Y.; Kitao, O.; Nakai, H.; Vreven, T.; Throssell, K.; Montgomery, J. A., Jr.; Peralta, J. E.; Ogliaro, F.; Bearpark, M. J.; Heyd, J. J.; Brothers, E. N.; Kudin, K. N.; Staroverov, V. N.; Keith, T. A.; Kobayashi, R.; Normand, J.; Raghavachari, K.; Rendell, A. P.; Burant, J. C.;

Iyengar, S. S.; Tomasi, J.; Cossi, M.; Millam, J. M.; Klene, M.; Adamo, C.; Cammi, R.; Ochterski, J. W.; Martin, R. L.; Morokuma, K.; Farkas, O.; Foresman, J. B.; Fox, D. J. *Gaussian 16*, Revision B.01, Gaussian, Inc., Wallingford CT, **2016**.

19. E. J. Baerends, E. J.; Gritsenko, O. V.; van Meer, R. *Phys. Chem. Chem. Phys.*, **2013**, *15*, 16408-16425.
20. Baik, M.-H.; Friesner, R. A. *J. Chem. Theory Comput.* **2013**, *9*, 381–393.
21. Lu, T.; Chen, F. *J. Comput. Chem.*, **2012**, *33*, 580-592.
22. Lu, T. *J. Chem. Phys.*, **2024**, *161*, 082503.

K. NMR spectra

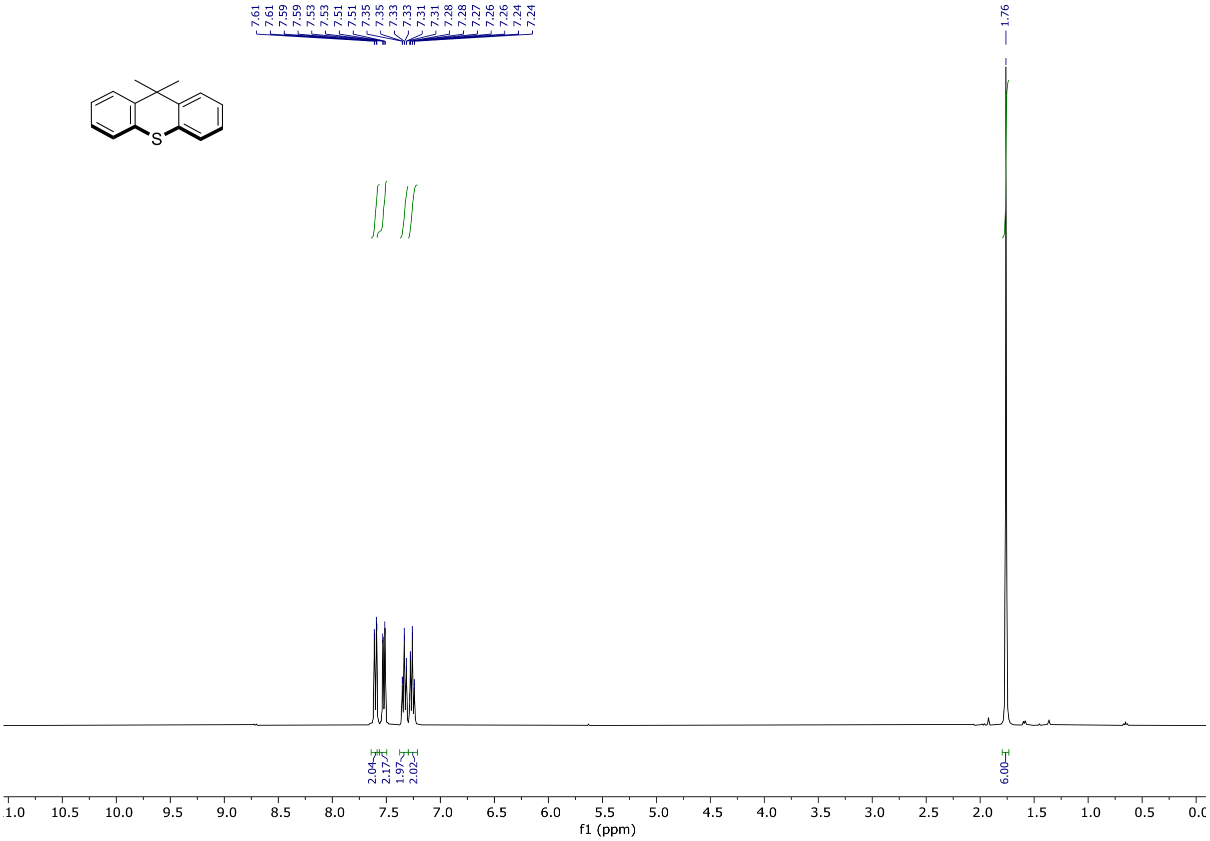

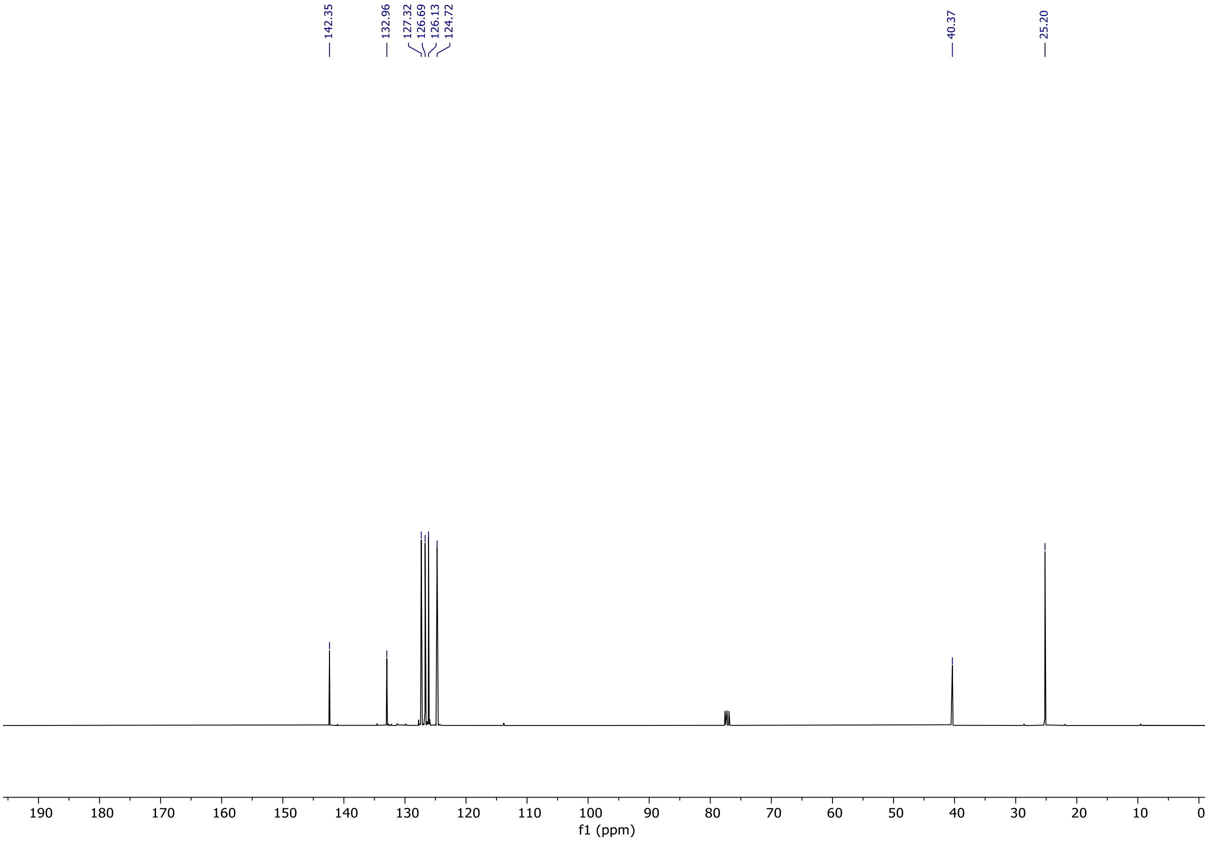

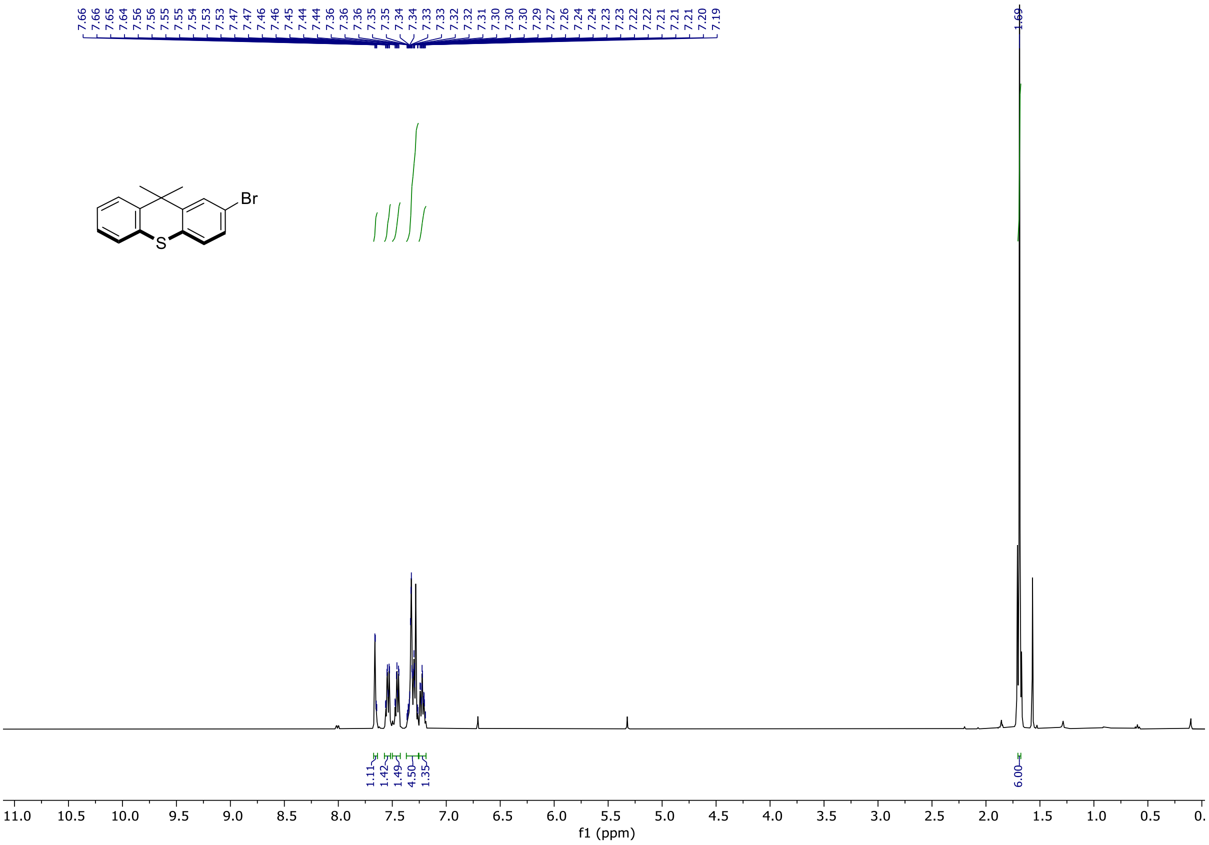
Br
S
6.00
f1 (ppm)

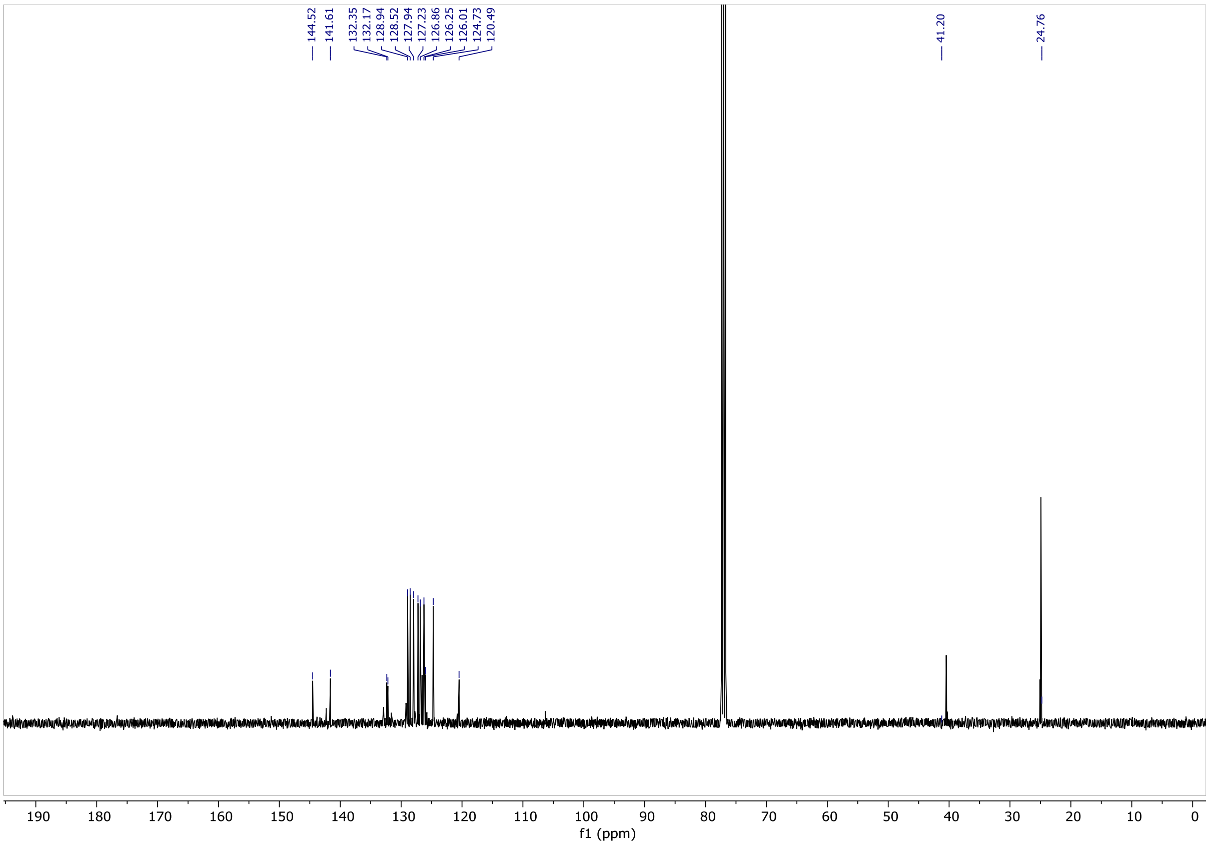
144.52
141.61
132.35
132.17
128.94
128.52
127.94
127.23
126.86
126.25
126.01
124.73
120.49
41.20
24.76
f1 (ppm)

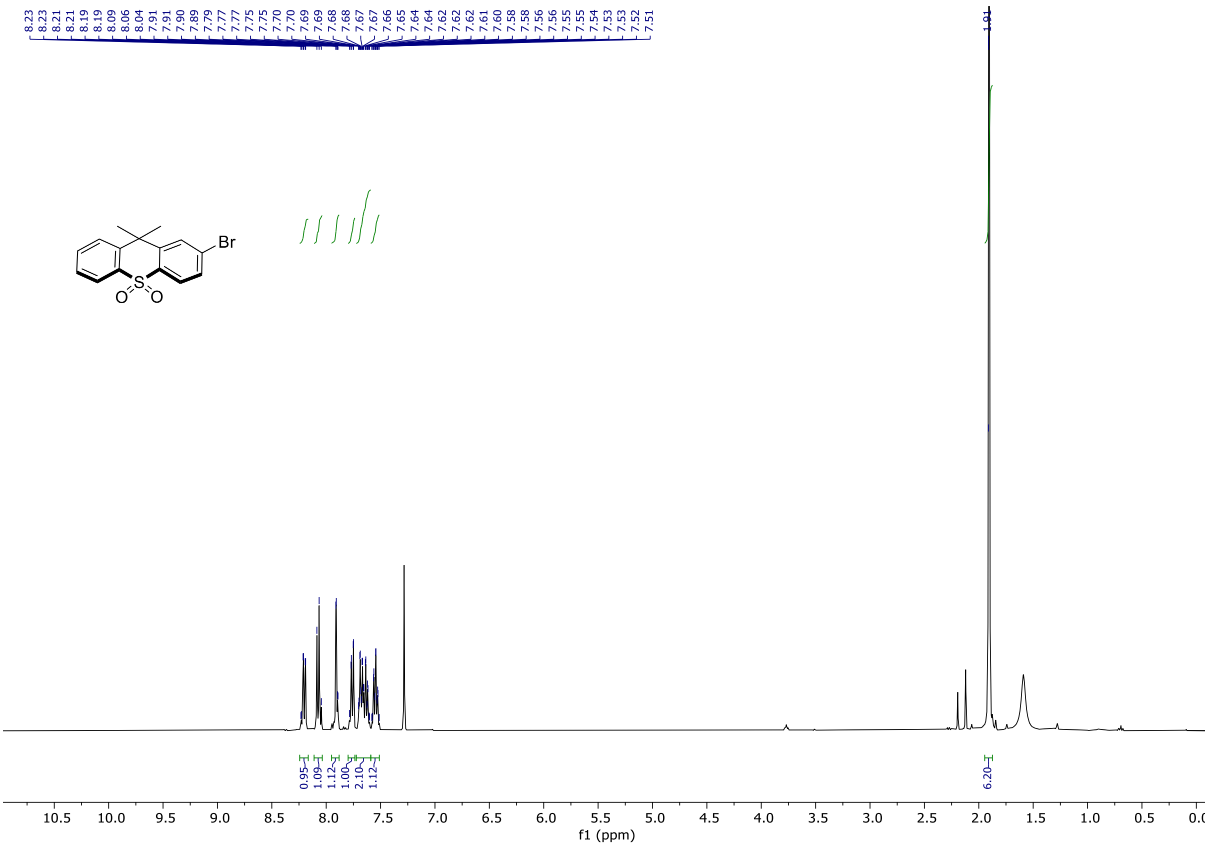
8.23
8.23
8.21
8.21
8.19
8.19
8.09
8.06
8.04
7.91
7.91
7.90
7.89
7.79
7.77
7.77
7.75
7.75
7.70
7.69
7.69
7.68
7.68
7.67
7.67
7.66
7.65
7.64
7.64
7.62
7.62
7.61
7.60
7.58
7.58
7.56
7.56
7.55
7.55
7.54
7.53
7.53
7.52
7.51
1.91
Br
S
O
O
0.95
1.09
1.12
1.00
2.10
1.12
6.20
f1 (ppm)

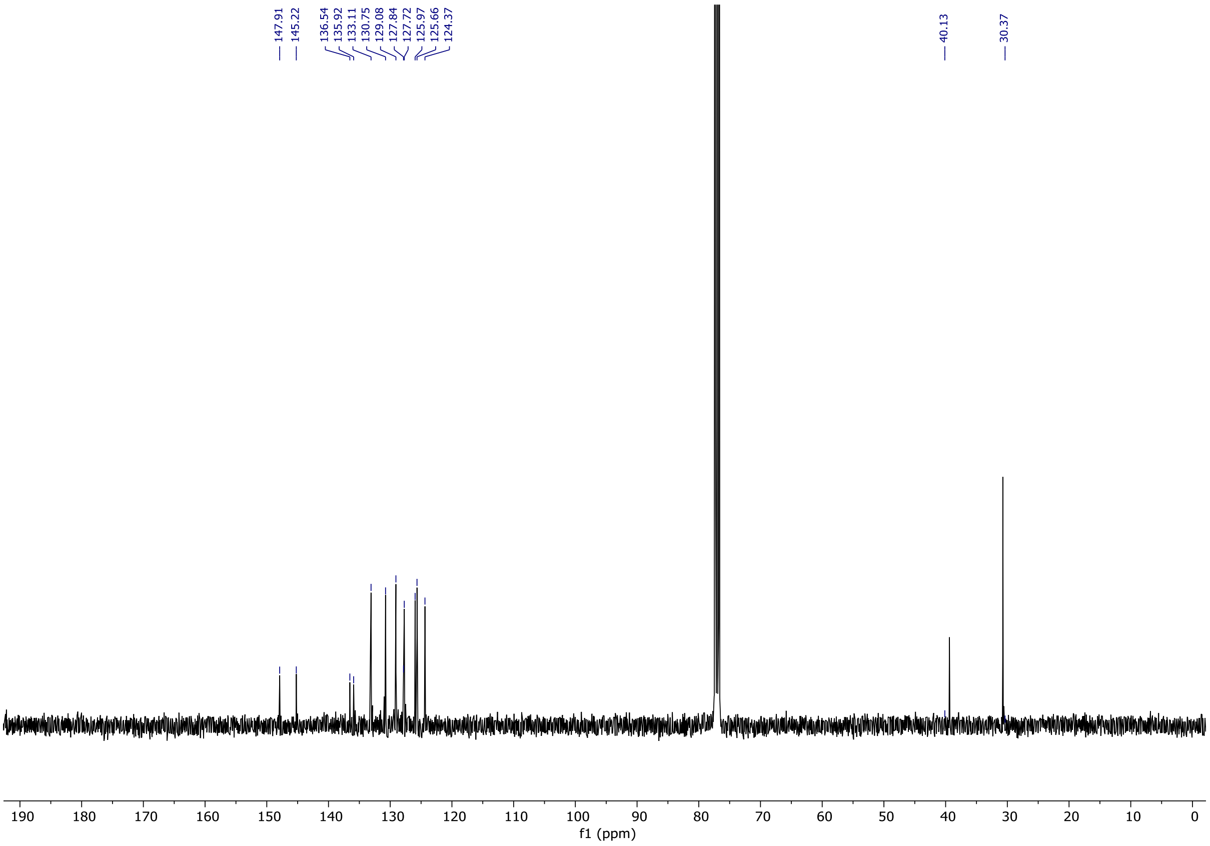
147.91
145.22
136.54
135.92
133.11
130.75
129.08
127.84
127.72
125.97
125.66
124.37
40.13
30.37
f1 (ppm)

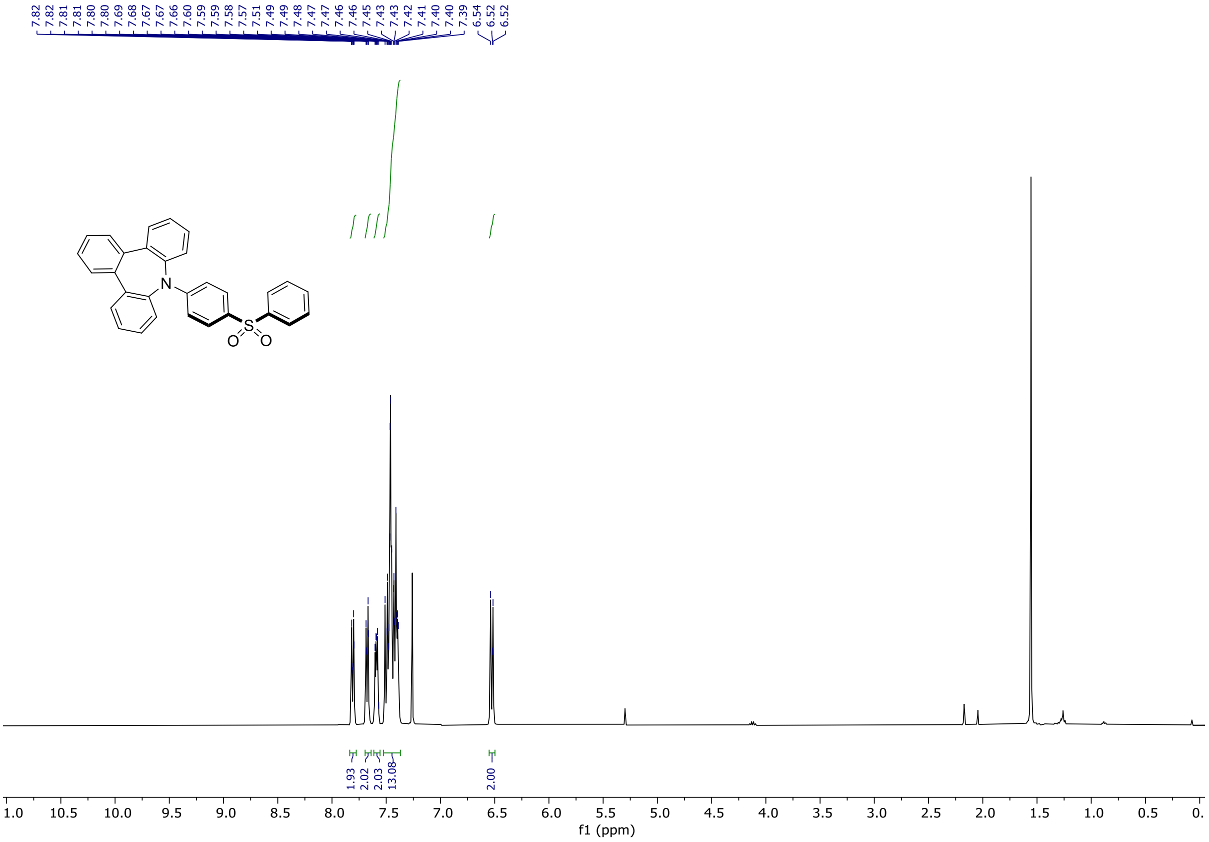
7.82 7.82 7.81 7.81 7.80 7.69 7.68 7.67 7.66 7.60 7.59 7.59 7.58 7.57 7.51 7.49 7.49 7.48 7.47 7.47 7.46 7.45 7.43 7.43 7.42 7.41 7.40 7.39 6.54 6.52 6.52
N
S
O
O
1.93
2.02
2.03
13.08
2.00
f1 (ppm)

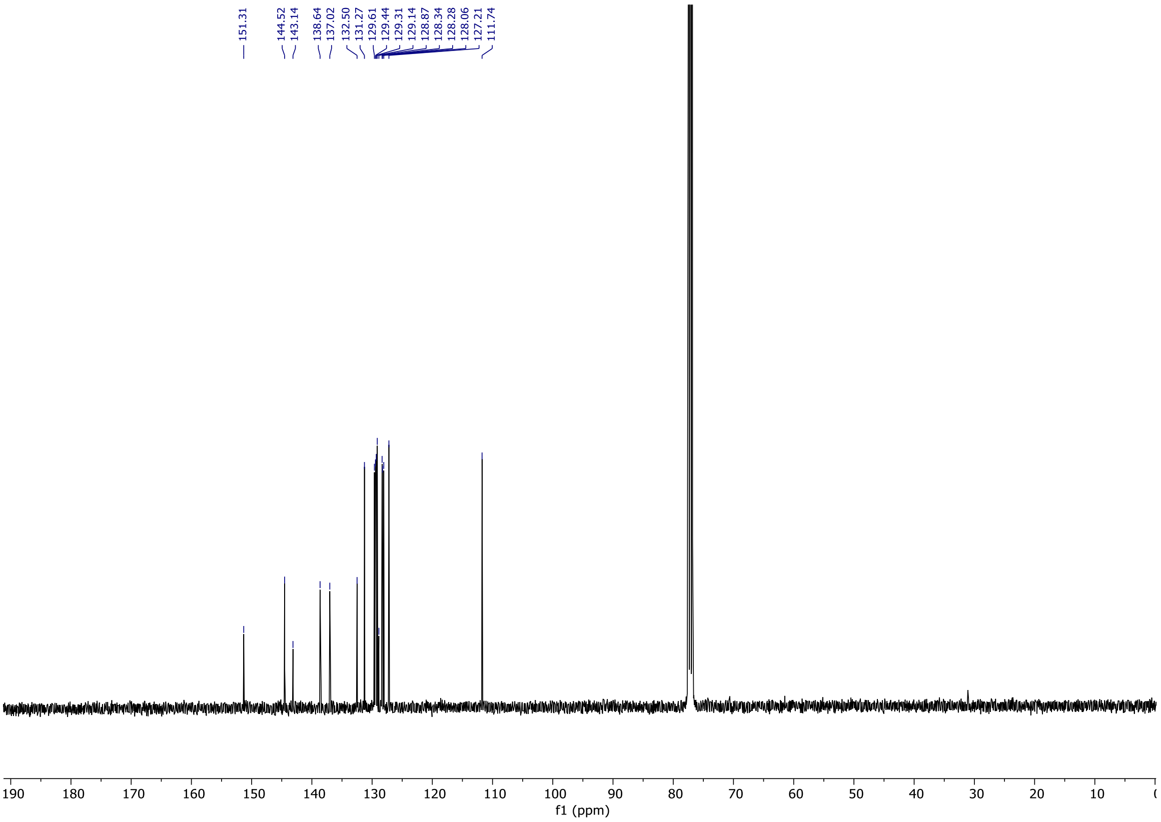
151.31
144.52 143.14
138.64 137.02
132.50 131.27 129.61 129.44 129.31 129.14 128.87 128.34 128.28 128.06 127.21
111.74
f1 (ppm)

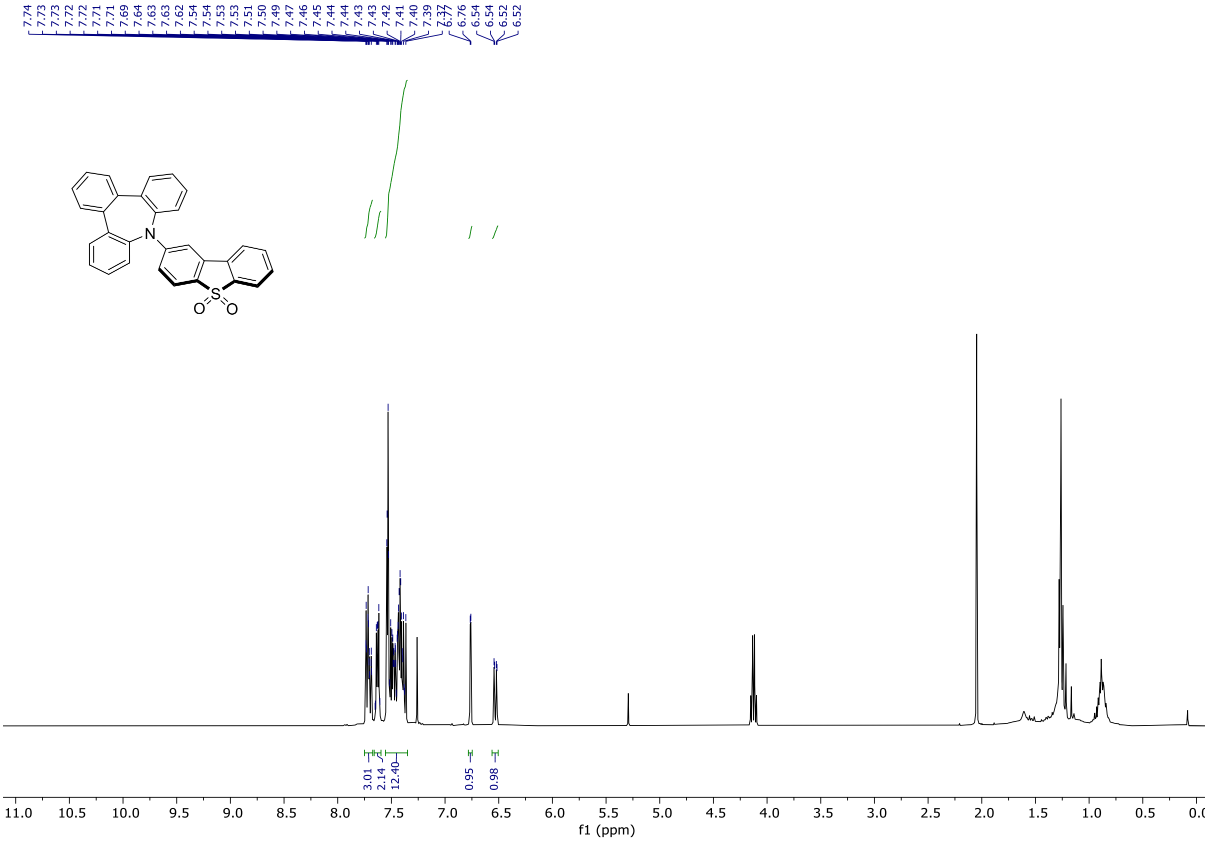
7.74
7.73
7.73
7.72
7.72
7.71
7.71
7.69
7.64
7.63
7.63
7.62
7.54
7.54
7.53
7.53
7.51
7.50
7.49
7.47
7.46
7.45
7.44
7.44
7.43
7.43
7.42
7.41
7.40
7.39
7.37
6.77
6.76
6.54
6.54
6.52
6.52
N
S
O
O
3.01
2.14
12.40
0.95
0.98
11.0 10.5 10.0 9.5 9.0 8.5 8.0 7.5 7.0 6.5 6.0 5.5 5.0 4.5 4.0 3.5 3.0 2.5 2.0 1.5 1.0 0.5 0.0
f1 (ppm)

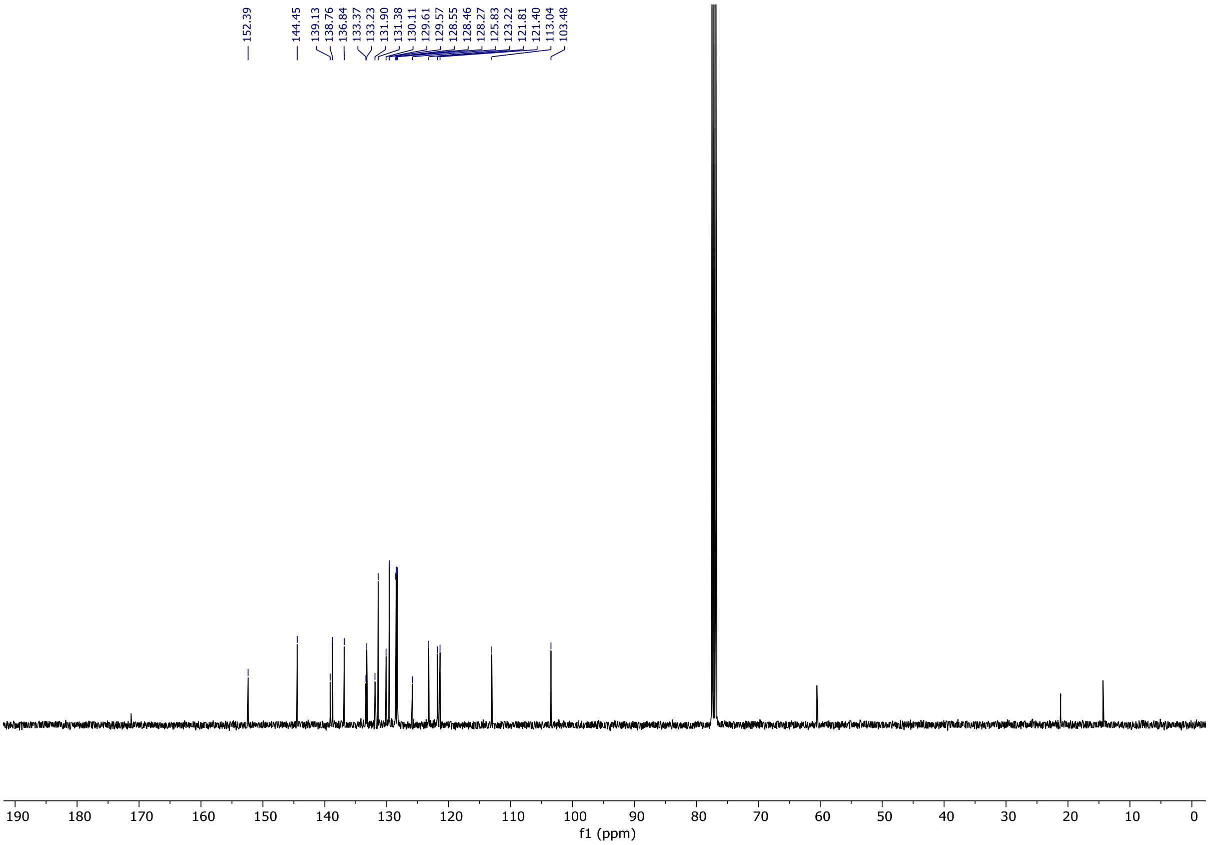
152.39
144.45
139.13
138.76
136.84
133.37
133.23
131.90
131.38
130.11
129.61
129.57
128.55
128.46
128.27
125.83
123.22
121.81
121.40
113.04
103.48
190 180 170 160 150 140 130 120 110 100 90 80 70 60 50 40 30 20 10 0
f1 (ppm)

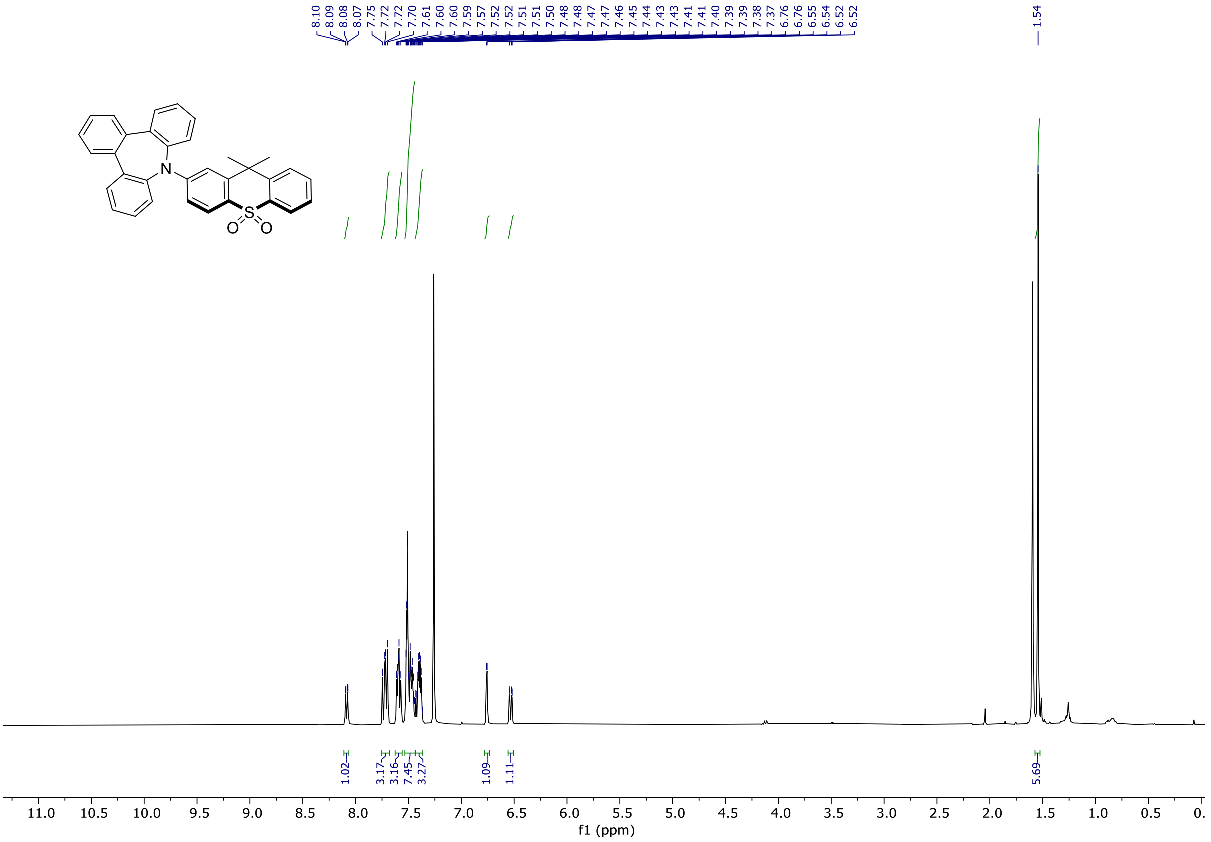
8.10
8.09
8.08
8.07
7.75
7.72
7.72
7.70
7.61
7.60
7.60
7.59
7.57
7.52
7.52
7.51
7.51
7.50
7.48
7.48
7.47
7.46
7.45
7.44
7.43
7.43
7.41
7.41
7.40
7.39
7.39
7.38
7.37
6.76
6.76
6.55
6.54
6.52
6.52
1.54
N
S
O
O
1.02
3.17
3.16
7.45
3.27
1.09
1.11
5.69
11.0 10.5 10.0 9.5 9.0 8.5 8.0 7.5 7.0 6.5 6.0 5.5 5.0 4.5 4.0 3.5 3.0 2.5 2.0 1.5 1.0 0.5 0.
f1 (ppm)

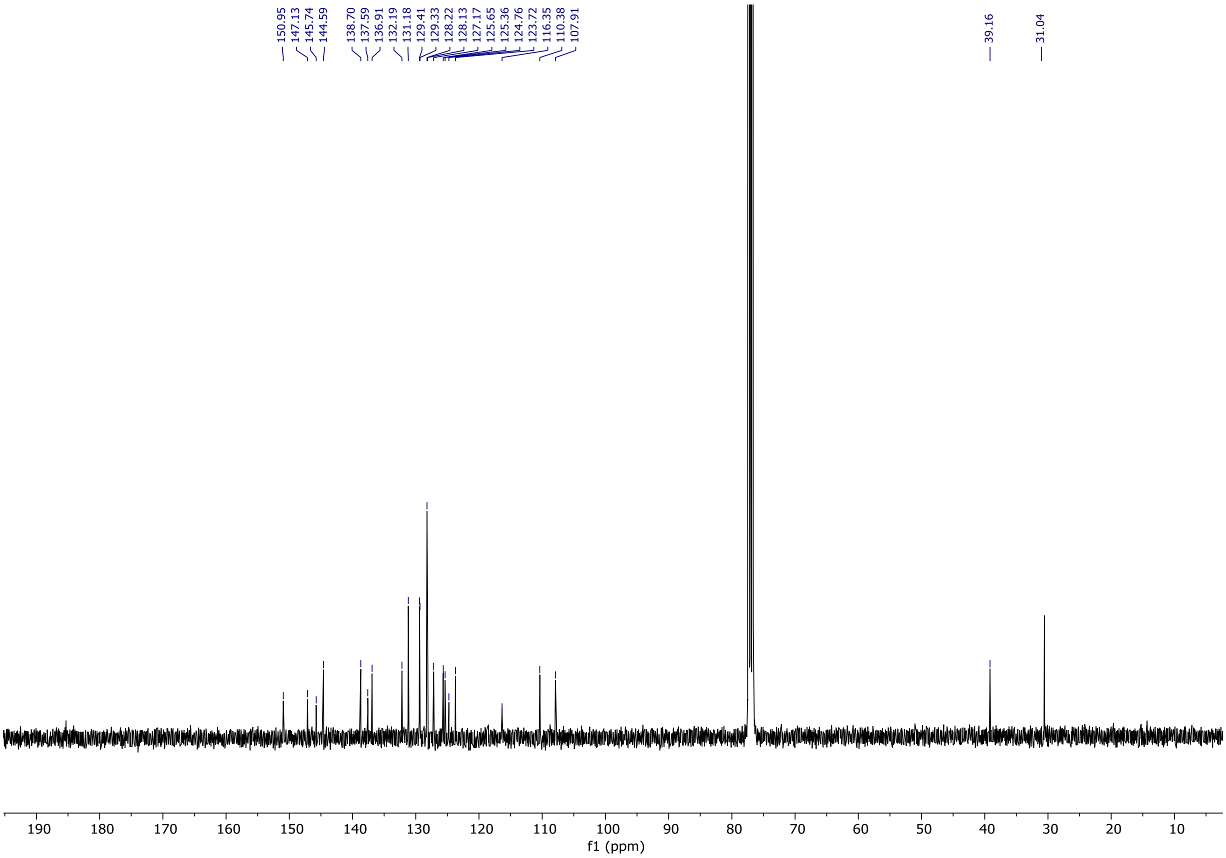
150.95
147.13
145.74
144.59
138.70
137.59
136.91
132.19
131.18
129.41
129.33
128.22
128.13
127.17
125.65
125.36
124.76
123.72
116.35
110.38
107.91
39.16
31.04
190 180 170 160 150 140 130 120 110 100 90 80 70 60 50 40 30 20 10
f1 (ppm)

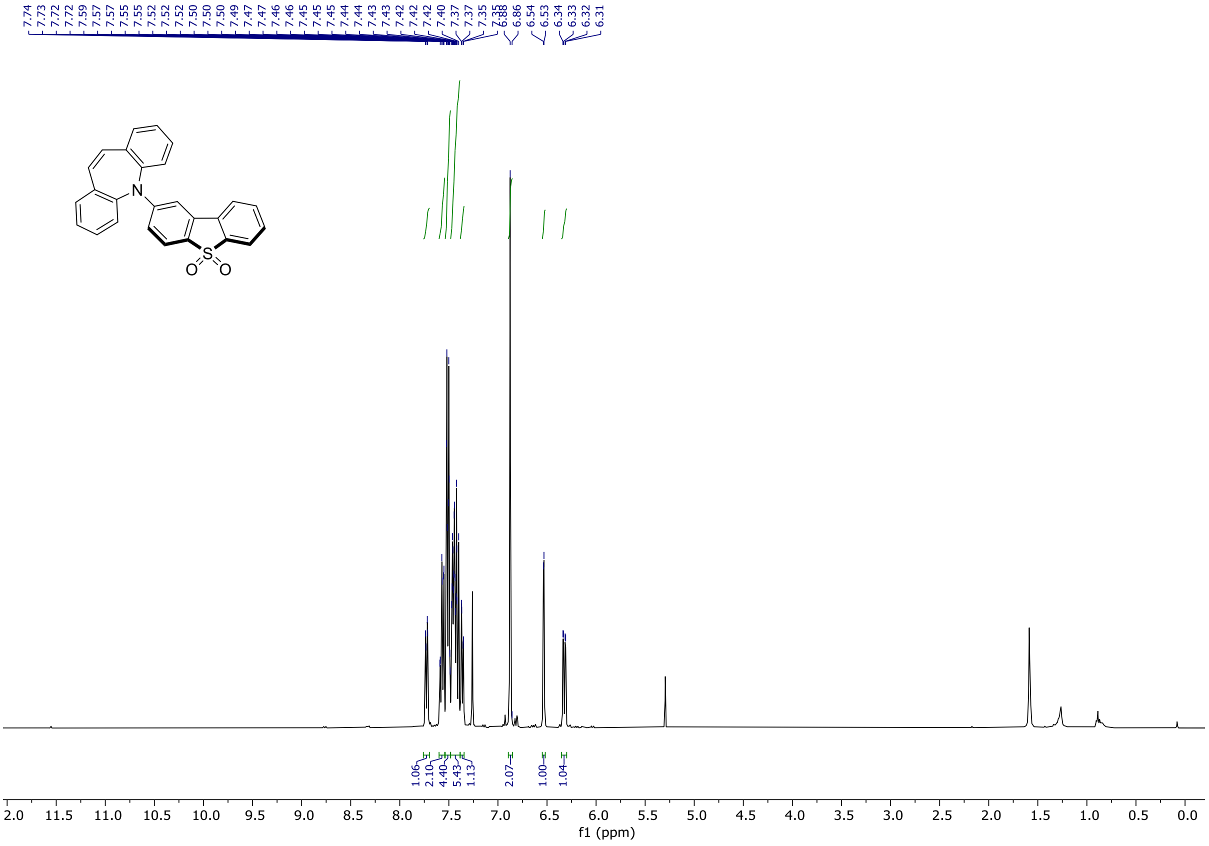
7.74
7.73
7.72
7.72
7.59
7.57
7.57
7.55
7.55
7.52
7.52
7.52
7.50
7.50
7.50
7.49
7.47
7.47
7.46
7.46
7.45
7.45
7.45
7.44
7.44
7.43
7.43
7.42
7.42
7.42
7.40
7.37
7.37
7.35
6.88
6.86
6.54
6.53
6.34
6.33
6.32
6.31
N
S
O
O
1.06
2.10
4.40
5.43
1.13
2.07
1.00
1.04
f1 (ppm)

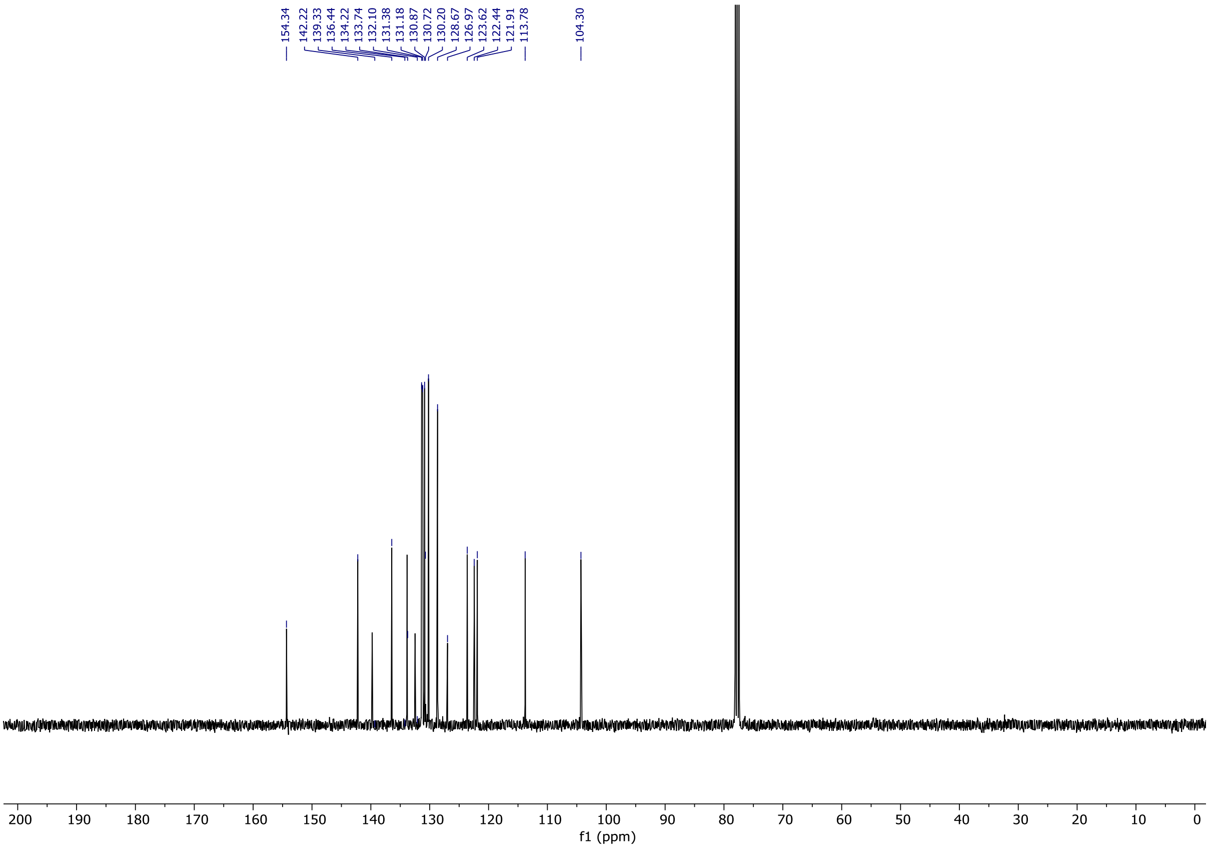
154.34
142.22
139.33
136.44
134.22
133.74
132.10
131.38
131.18
130.87
130.72
130.20
128.67
126.97
123.62
122.44
121.91
113.78
104.30
f1 (ppm)

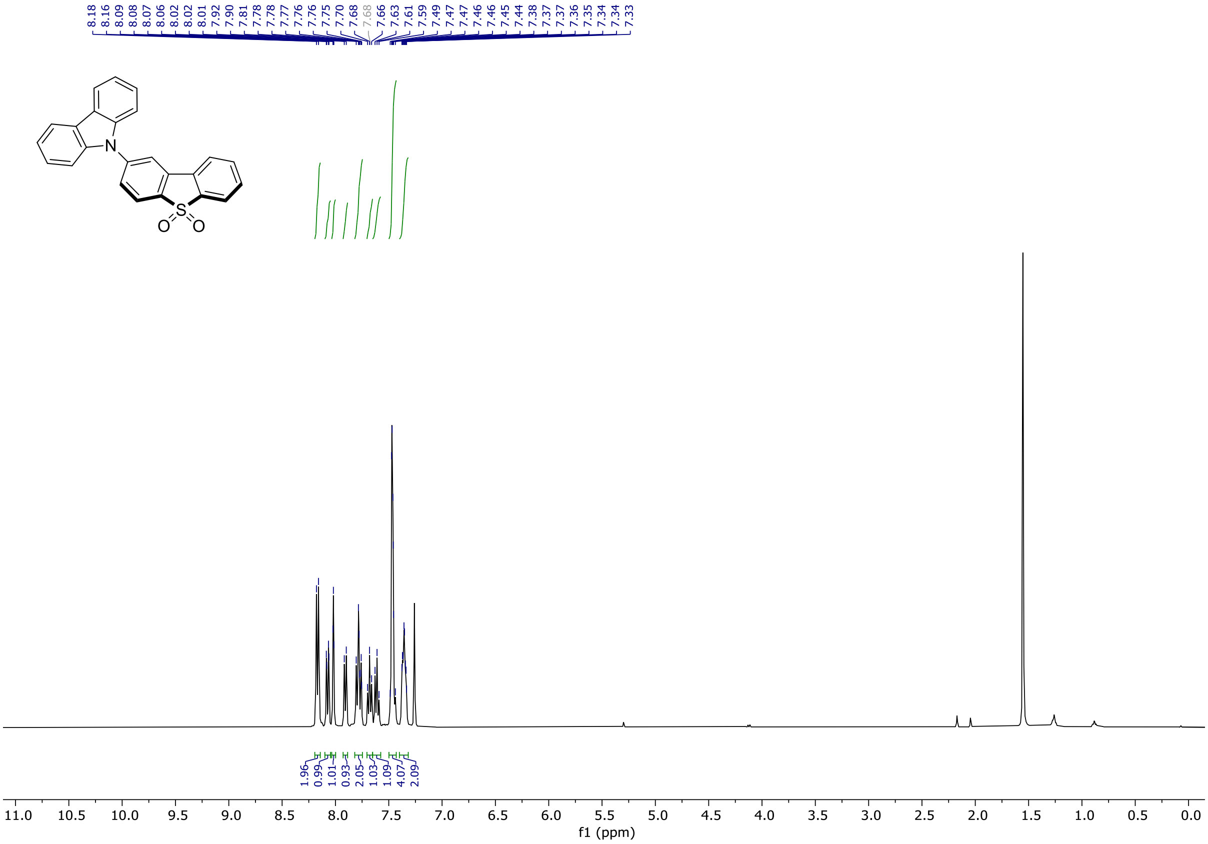
8.18 8.16 8.09 8.08 8.07 8.06 8.02 8.02 8.01 7.92 7.90 7.81 7.78 7.78 7.77 7.76 7.76 7.75 7.70 7.68 7.68 7.66 7.63 7.61 7.59 7.49 7.47 7.47 7.46 7.46 7.45 7.44 7.38 7.37 7.37 7.36 7.35 7.34 7.34 7.33
N
S
O O
1.96 0.99 1.01 0.93 2.05 1.03 1.09 4.07 2.09
11.0 10.5 10.0 9.5 9.0 8.5 8.0 7.5 7.0 6.5 6.0 5.5 5.0 4.5 4.0 3.5 3.0 2.5 2.0 1.5 1.0 0.5 0.0
f1 (ppm)

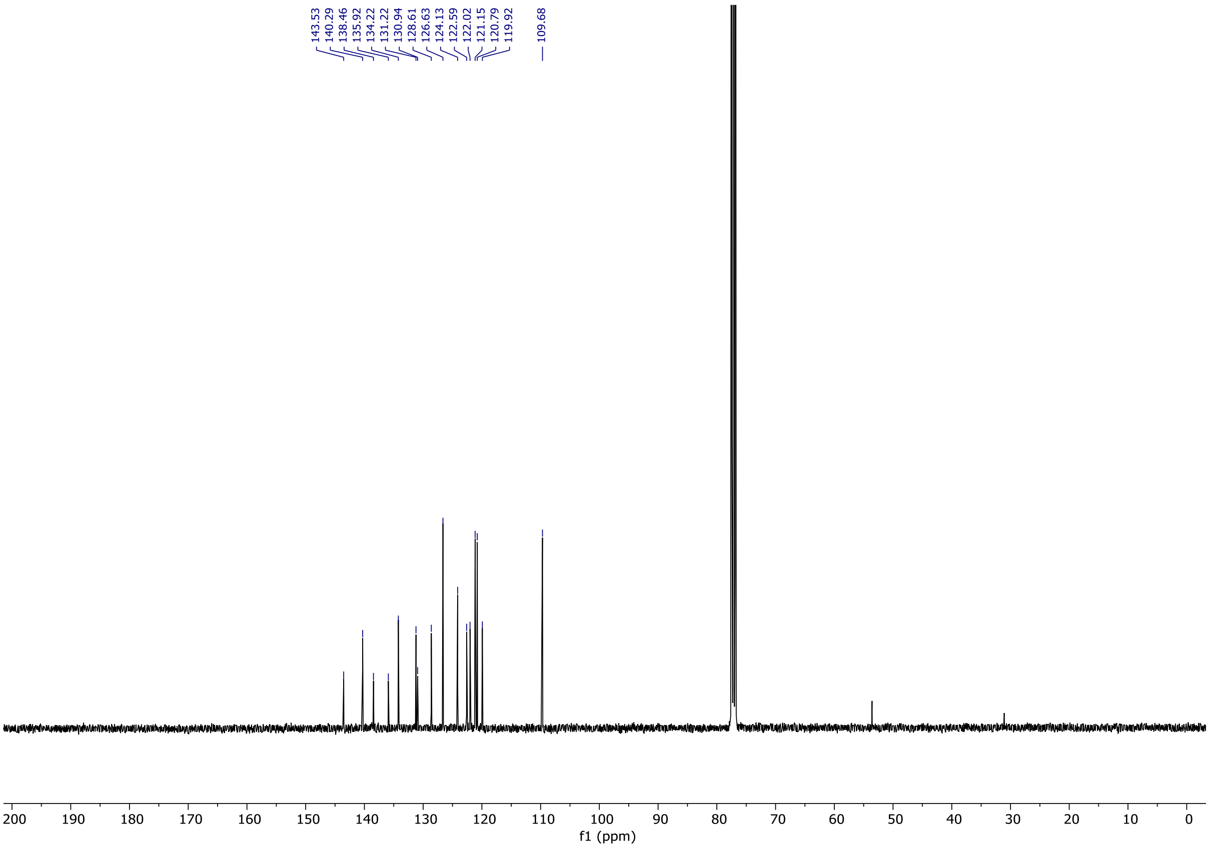
143.53 140.29 138.46 135.92 134.22 131.22 130.94 128.61 126.63 124.13 122.59 122.02 121.15 120.79 119.92
109.68
200 190 180 170 160 150 140 130 120 110 100 90 80 70 60 50 40 30 20 10 0
f1 (ppm)

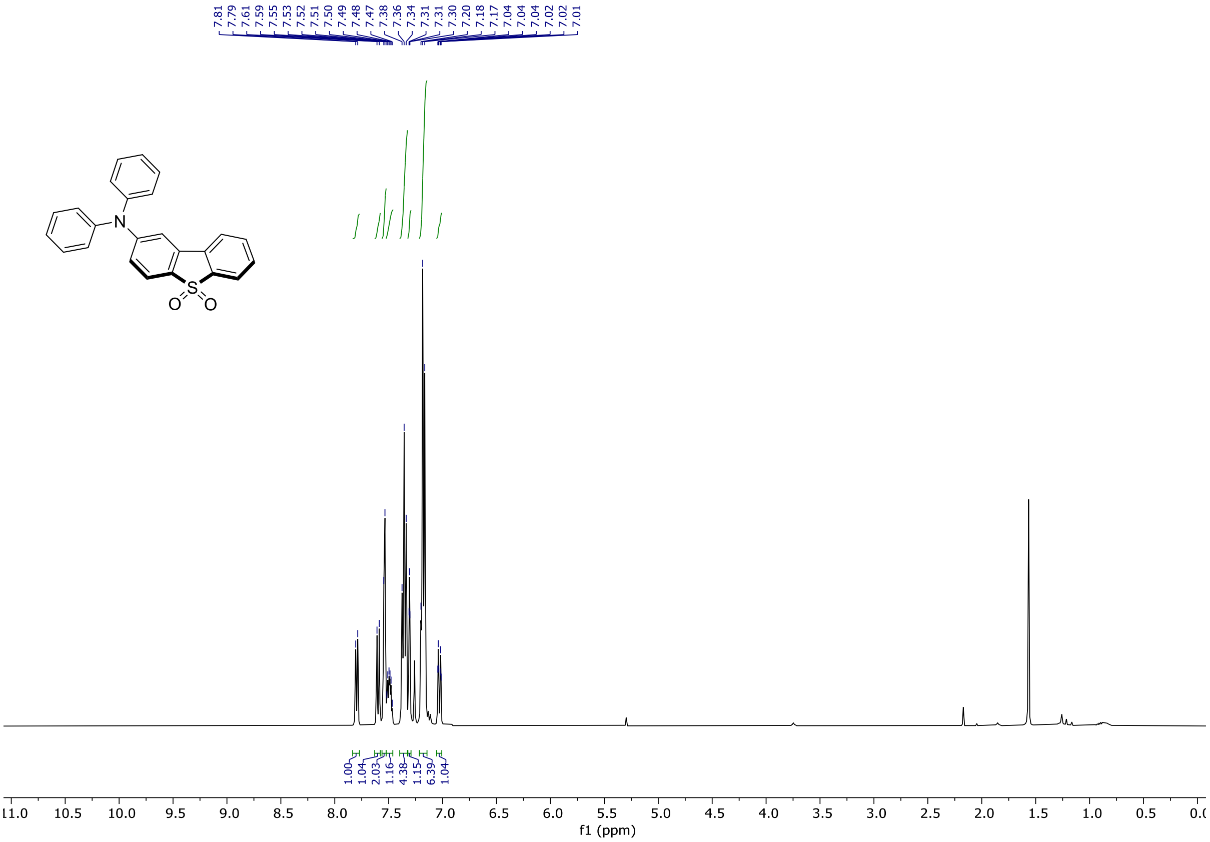
7.81
7.79
7.61
7.59
7.55
7.53
7.52
7.51
7.50
7.49
7.48
7.47
7.38
7.36
7.34
7.31
7.31
7.30
7.20
7.18
7.17
7.04
7.04
7.04
7.02
7.01
N
S
O
O
1.00
1.04
2.03
1.16
4.38
1.15
6.39
1.04
11.0 10.5 10.0 9.5 9.0 8.5 8.0 7.5 7.0 6.5 6.0 5.5 5.0 4.5 4.0 3.5 3.0 2.5 2.0 1.5 1.0 0.5 0.0
f1 (ppm)

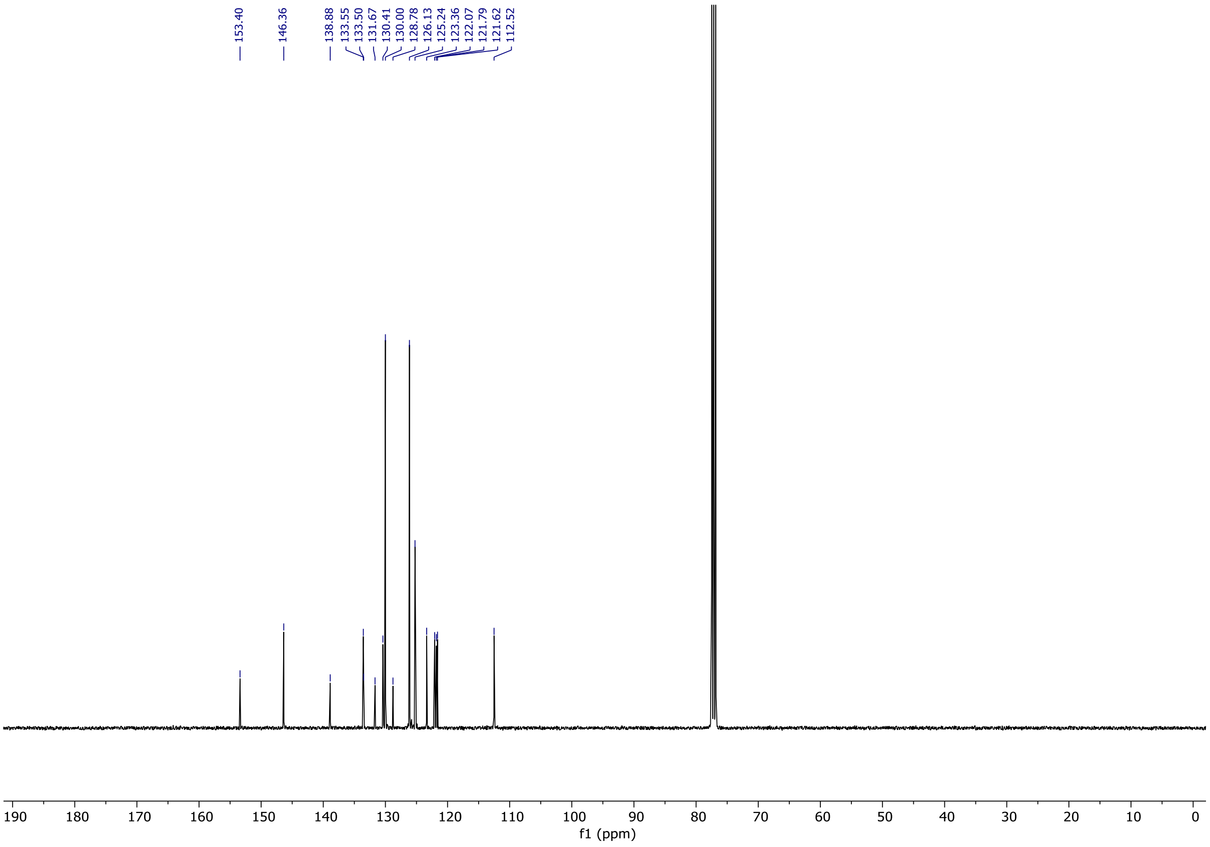
153.40
146.36
138.88
133.55
133.50
131.67
130.41
130.00
128.78
126.13
125.24
123.36
122.07
121.79
121.62
112.52
190 180 170 160 150 140 130 120 110 100 90 80 70 60 50 40 30 20 10 0
f1 (ppm)

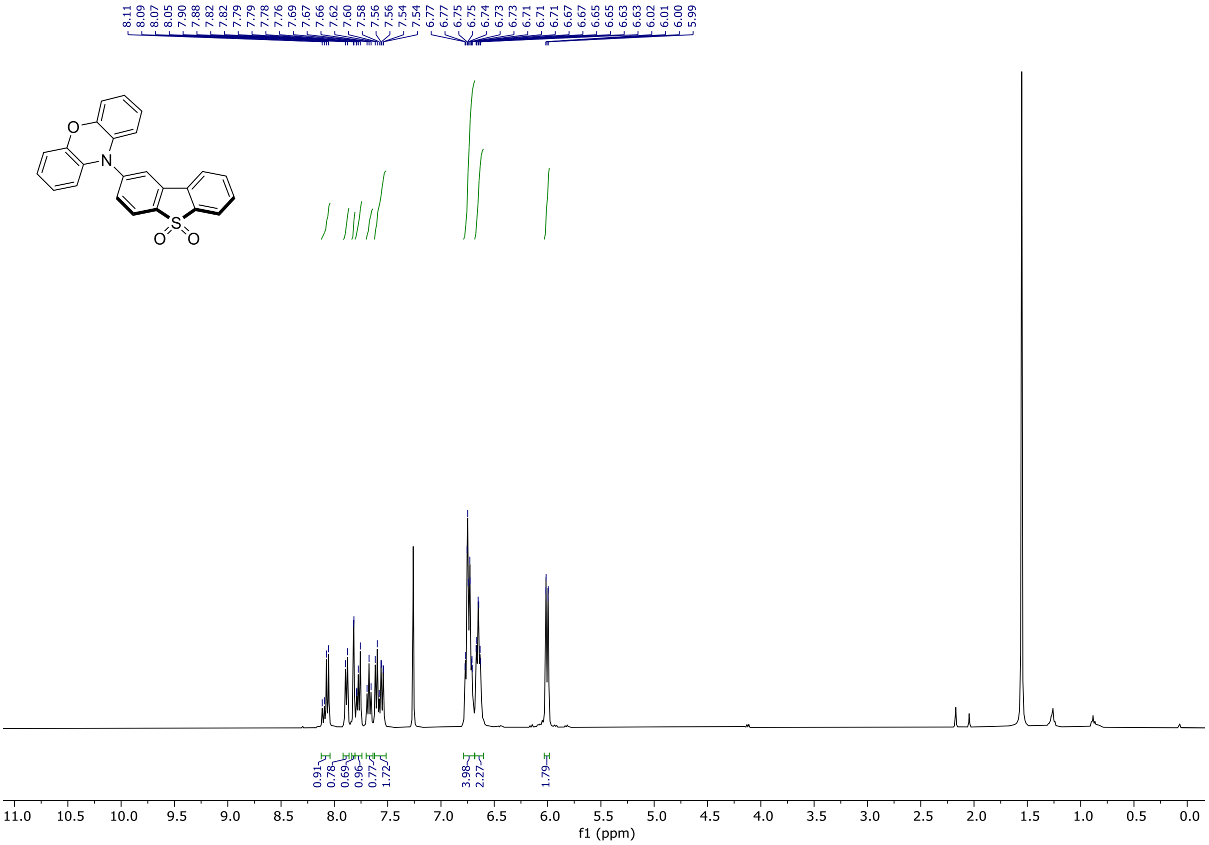
8.11 8.09 8.07 8.05 7.90 7.88 7.82 7.82 7.79 7.79 7.78 7.76 7.69 7.67 7.66 7.62 7.60 7.58 7.56 7.56 7.54 7.54 6.77 6.77 6.75 6.75 6.74 6.73 6.73 6.71 6.71 6.71 6.67 6.67 6.65 6.65 6.63 6.63 6.02 6.01 6.00 5.99
O
N
S
O O
0.91 0.78 0.69 0.96 0.77 1.72 3.98 2.27 1.79
11.0 10.5 10.0 9.5 9.0 8.5 8.0 7.5 7.0 6.5 6.0 5.5 5.0 4.5 4.0 3.5 3.0 2.5 2.0 1.5 1.0 0.5 0.0
f1 (ppm)

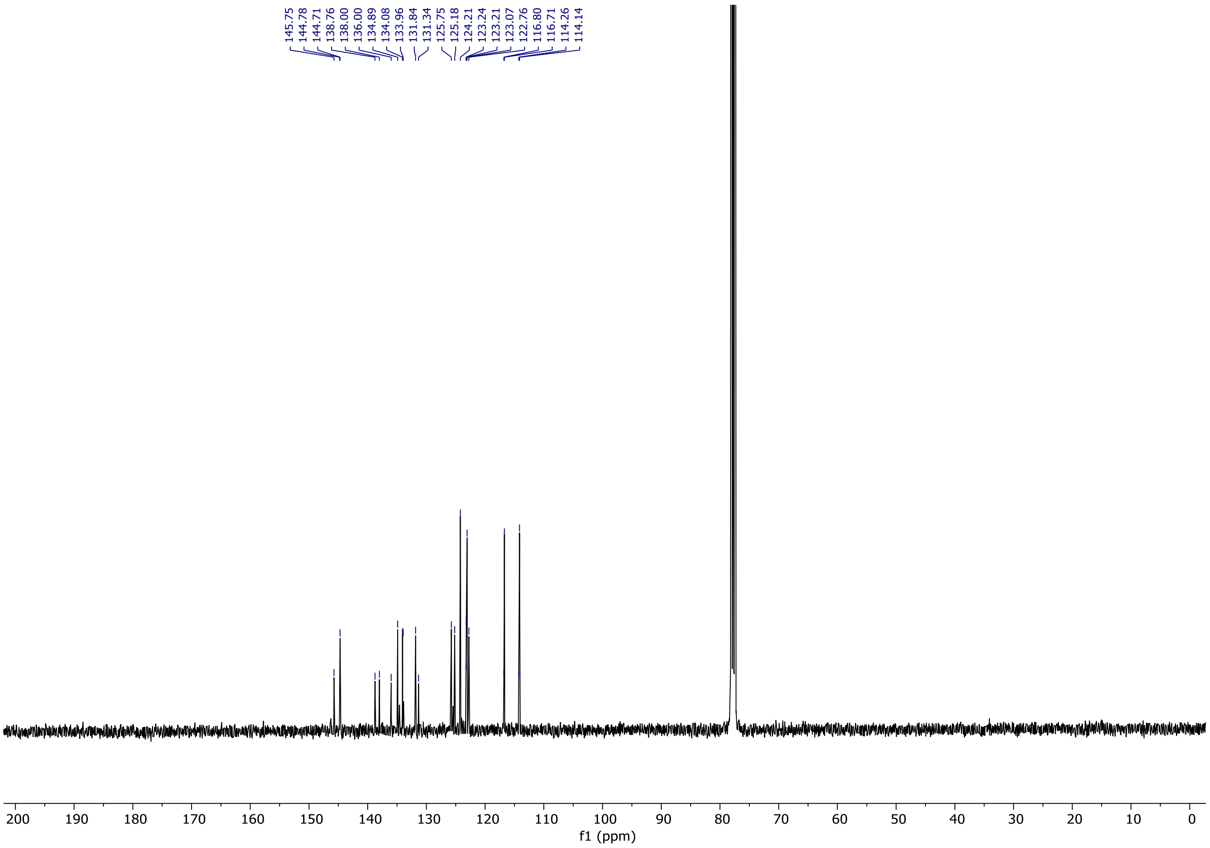
145.75 144.78 144.71 138.76 138.00 136.00 134.89 134.08 133.96 131.84 131.34 125.75 125.18 124.21 123.24 123.21 123.07 122.76 116.80 116.71 114.26 114.14
200 190 180 170 160 150 140 130 120 110 100 90 80 70 60 50 40 30 20 10 0
f1 (ppm)
